# Efficacy and safety of direct oral anticoagulants approved for cardiovascular indications: Systematic review and meta-analysis

**DOI:** 10.1371/journal.pone.0197583

**Published:** 2018-05-24

**Authors:** Raghavendra Charan P. Makam, David C. Hoaglin, David D. McManus, Victoria Wang, Joel M. Gore, Frederick A. Spencer, Richeek Pradhan, Hoang Tran, Hong Yu, Robert J. Goldberg

**Affiliations:** 1 Department of Quantitative Health Sciences, University of Massachusetts Medical School, Worcester, MA, United States of America; 2 Department of Medicine, University of Massachusetts Medical School, Worcester, MA, United States of America; 3 Division of Cardiac Surgery, Department of Surgery, Johns Hopkins University, Baltimore, MD, United States of America; 4 Department of Medicine, McMaster University, Hamilton, Canada; 5 Department of Computer Science, University of Massachusetts, Amherst, MA, United States of America; 6 Center for Healthcare Organization & Implementation Research, Edith Nourse Rogers Memorial Veterans Hospital, Bedford, MA, United States of America; 7 Department of Computer Science, University of Massachusetts, Lowell, MA, United States of America; University of Bologna, ITALY

## Abstract

**Background:**

Direct oral anticoagulants (DOACs) have emerged as promising alternatives to vitamin K antagonists (VKAs) for patients with non-valvular atrial fibrillation (NVAF) or venous thromboembolism (VTE). Few meta-analyses have included all DOACs that have received FDA approval for these cardiovascular indications, and their overall comparisons against VKAs have shortcomings in data and methods. We provide an updated overall assessment of the efficacy and safety of those DOACs at dosages currently approved for NVAF or VTE, in comparison with VKAs.

**Methods:**

We used data from Phase 3 randomized trials that compared an FDA-approved DOAC with VKA for primary prevention of stroke in patients with NVAF or for treatment of acute VTE.

**Results:**

Among trial participants with NVAF, DOAC recipients had a lower risk of stroke or systemic embolism [Pooled Odds Ratio (OR) 0.76, 95% Confidence Interval (CI) (0.68–0.84)], any stroke (0.80, 0.73–0.88), systemic embolism (0.56, 0.34–0.93), and total mortality (0.89, 0.84–0.95). Safety outcomes also showed a lower risk of fatal, major, and intracranial bleeding but higher risk for gastrointestinal bleeding (GIB). Patients with acute VTE randomized to DOACs had comparable risk of recurrent VTE and death (OR 0.88, 95% CI 0.75–1.03), recurrent DVT (0.83, 0.66–1.05), recurrent non-fatal PE (0.97, 0.75–1.25), and total mortality (0.94, 0.79–1.12). Safety outcomes for DOACs showed a lower risk of major, fatal, and intracranial bleeding, but similar risk of GIB.

**Conclusions:**

Patients receiving DOACs for NVAF had predominantly superior efficacy and safety. Patients who were treated with DOACs for acute VTE had non-inferior efficacy, but an overall superior safety profile.

## Introduction

Since the approval of dabigatran by regulatory agencies in Europe and Canada in 2008[1, 2], and in the United States in 2010[3], the use of direct oral anticoagulants (DOACs) has increased dramatically[4, 5]. Four DOACs, the direct thrombin inhibitor dabigatran and the Factor Xa inhibitors rivaroxaban, apixaban, and edoxaban, are currently approved for use in Europe. The U.S. guidelines recommend these agents as alternatives to vitamin K antagonists (VKAs) for prevention of thromboembolism in patients with non-valvular atrial fibrillation (NVAF) and for treatment of acute venous thromboembolism (VTE)[6–10].

The increase in prescriptions for DOACs in patients with these cardiovascular indications reflects several advantages for DOACs over VKAs, including fixed-dose administration, fewer drug-drug interactions, and limited dietary restrictions. Although clinical trials have demonstrated at least equivalent therapeutic efficacy of these newer agents[11–19], concerns about the safety profile and net clinical benefit of DOACs have remained, perhaps because of anecdotal reports of adverse outcomes and experience with some early DOACs, which were withdrawn from the market because of serious adverse events[20–22]. The uncertainty arising from conflicting results from clinical trials, post-market surveillance and observational studies, and systematic reviews[23–28], issues of long-term safety and higher cost, and the absence of approved reversal agents for Factor Xa antagonists[29] are of particular concern to patients, pharmacists, and clinicians, limiting the routine use of DOACs even among those with approved indications[30].

Most systematic reviews and meta-analyses that have examined the efficacy and safety of DOACs were conducted before the FDA approved edoxaban for use in patients with NVAF and VTE in 2015[31]. Many also included studies that used DOACs for multiple cardiac and non-cardiac conditions and at various dosages, many of which were eventually not approved for clinical use by the FDA. Although including such an expanded list of indications might be valuable for a researcher, the practicing cardiologist is often more interested in the expected outcomes associated with the use of a specific medication, when used for approved cardiovascular indications alone and at FDA-approved dosages, as relevant to their current clinical practice. Finally, several methodological shortcomings in prior meta-analyses (described in S10 File) raise doubts about applying their conclusions to the contemporary use of DOACs in patients with specific cardiovascular indications.

To address ongoing concerns about the efficacy, safety, and net clinical benefit of DOACs as a therapeutic class when used for on-label cardiovascular indications, we performed a systematic review and meta-analysis of important efficacy and safety outcomes. The data came from all high-quality Phase 3 randomized clinical trials of the 4 FDA-approved DOACs at currently approved dosages for prevention of thromboembolic stroke in patients with NVAF and for treatment of acute VTE.

## Methods

### Search strategy

We performed a contemporary systematic review of the published literature in accordance with the Preferred Reporting Items for Systematic Reviews and Meta-Analyses (PRISMA) guidelines (S1 File). We searched PubMed (including MEDLINE) and Scopus (including Embase) databases and Cochrane libraries for randomized trials published from inception of the databases through July 2016. We also searched on Google Scholar and reviewed citations of published review articles to find additional clinical trials. The search terms and protocol for this systematic review and meta-analysis are documented in S2 File.

### Study selection

To be included in this meta-analysis, a Phase 3 clinical trial must have been reported in English and have compared dabigatran, apixaban, rivaroxaban, or edoxaban at standard dosages (as described in S2 File) versus warfarin (dose-adjusted to achieve an international normalized ratio between 2.0 and 3.0) for prevention of thromboembolism in patients with NVAF or for treatment of acute VTE. We excluded studies of DOACs for indications other than these two conditions (e.g., DVT prophylaxis in patients undergoing hip and knee surgery) or only at dosages other than those approved by the FDA. In stages, we reviewed each article’s title, abstract, and full text to eliminate articles that did not meet our pre-specified inclusion/exclusion criteria.

### Data collection and abstraction

For randomized trials meeting our inclusion criteria, two researchers (HT and RP) independently extracted pertinent information onto a standard data collection form (S5 File). Information abstracted included: lead author’s name, year of publication, clinical trial name, treatment indication, DOAC studied, dosages, comparator, participants’ age and sex, follow-up duration, and all efficacy and safety outcomes. The two reviewers resolved discrepancies through discussions between themselves and senior study investigators.

### Study outcomes

The study outcomes considered were the occurrence of stroke and systemic embolism (primary efficacy outcome for NVAF), any stroke, fatal and non-fatal PE, myocardial infarction, death from vascular causes, recurrent DVT or PE, recurrent VTE and related death (primary efficacy outcome for VTE), all-cause mortality, bleeding (major bleeding, fatal bleeding, intracranial bleeding, gastrointestinal (GI) bleeding, blood loss >2g/dl, blood transfusion of more than 2 units, intramuscular bleeding, non-intracranial/non-GI bleeding, and any overt bleeding), and other adverse drug events (ADEs) such as purpura, dizziness, diarrhea, edema, fatigue, epistaxis, headache, and serum liver transaminase levels greater than three times the upper limit of normal. An additional secondary outcome in some VTE trials was net clinical benefit, defined as the composite endpoint of recurrent VTE or a major bleeding episode. Because the sample sizes for several outcomes were relatively small, our analysis used 7 efficacy and 4 safety outcomes for trials in patients with NVAF and 7 efficacy and 8 safety outcomes for trials in patients with VTE.

### Statistical analysis

Because direct thrombin inhibitors and factor Xa inhibitors all act on the coagulation cascade, we considered the 4 DOACs as a single drug class and did not analyze differences among them. We carried out separate analyses of studies in patients with NVAF and VTE for outcomes that were reported by at least 3 trials. Since the trials were conducted under varying circumstances, our meta-analyses used a random-effects model. We do not report the heterogeneity measure *I*^2^ because it has no useful interpretation (as explained in S7 File). The quantitative measure of effect was the odds ratio (the ratio of the odds of an event in the DOAC arm to the odds of an event in the comparator arm, further explained in S6 File). The number of events and the number of subjects in each trial arm served as the data for a mixed-effects logistic regression model, in which a random effect accounted for variation among the various studies’ true log-odds-ratios. We used the lme4 package in R to conduct our analyses[32] (We report details of the models in S9 File).

This study was not subject to IRB approval since it was a summary analysis of existing published data.

## Results

**Fig 1:**
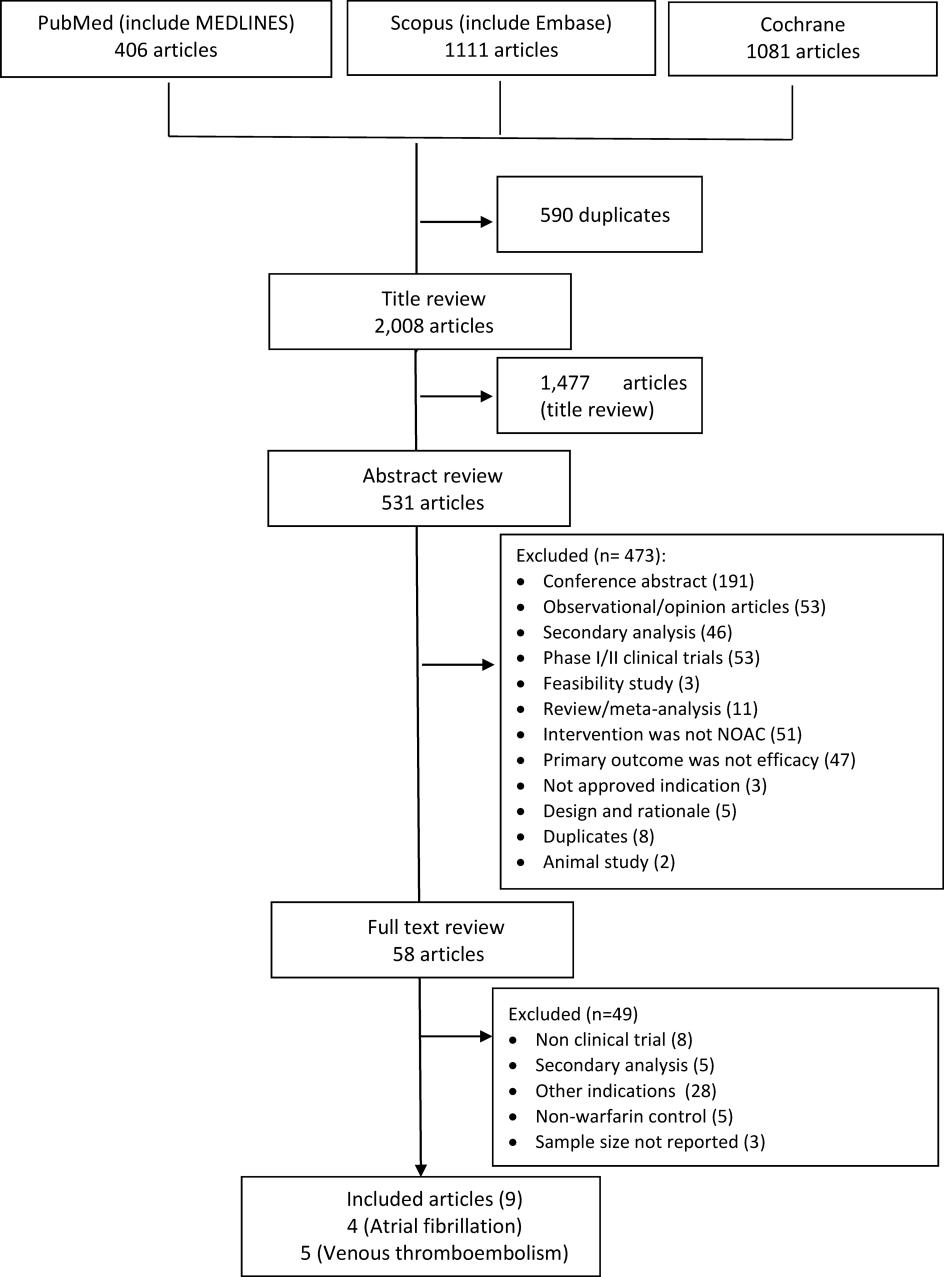
Study flow diagram.

Searches of PubMed, Scopus, and the Cochrane libraries yielded a total of 406, 1,111, and 1,081 articles, respectively (Fig 1). After excluding 590 duplicate articles and 1,477 by title review, we reviewed the abstracts of 531 articles for their possible relevance. Of the 58 articles retained for full-text review, 8 had a non-clinical trial design, 5 were a secondary analysis of a clinical trial, 28 studied patients with a non-approved or non-cardiac indication, 5 used non-warfarin agents as control, and 3 articles did not have information on the study’s sample size (S3 File). These exclusions left a total of 4 trials on stroke prevention in patients with NVAF[11–14] and 5 trials in patients with VTE[15–19] for data extraction.

### Study characteristics

The 9 randomized trials involved a total of 80,311 participants (58,271 in the NVAF trials and 22,040 in the VTE trials). The baseline characteristics of patients included in these trials are summarized in Table 1. In the trials of patients with NVAF, the average age of study participants was 71.6 years (unweighted average over trials), and 37.4% were women. Study sample sizes ranged from 12,037 to 18,201 patients, and the median duration of follow-up ranged from 1.8 to 2.8 years. In the trials of patients with VTE, the average age of study participants was 56.2 years, 41.6% were women, and study sample sizes ranged from 2,539 to 8,240 patients. None of the trials of patients with VTE reported median duration of follow-up; follow-up was longer than 1 year in one trial and longer than 6 months in the other four trials.

**Table 1:** Baseline characteristics and corresponding primary efficacy outcomes of the Phase 3 trials included.

NVAF Studies							
Study	Publication Year	DOAC and dosing regimen	Primary Events /Total N	Comparator	Primary Events /Total N	Age Years	Women %
RE-LY	2009	Dabigatran 150mg twice daily	182/6015	Warfarin	199/6022	71.6	36.8
ARISTOTLE	2011	Apixaban 5mg twice daily	212/9120	Warfarin	265/9081	70.0	35.2
ROCKET AF	2011	Rivaroxaban 20mg daily	188/6958	Warfarin	241/7004	73.0	39.7
ENGAGE AF- TIMI 48	2013	Edoxaban 60mg daily	296/7035	Warfarin	337/7036	72.0	37.7
**VTE Studies**							
RE-COVER	2009	Dabigatran 150mg twice daily	30/1274	Warfarin	27/1265	55.5	41.6
EINSTEIN-DVT	2010	Rivaroxaban 15mg twice daily for 3 weeks, followed by 20mg daily	36/1731	Enoxaparin followed by VKA	51/1718	56.1	43.2
AMPLIFY	2013	Apixaban 10mg twice daily for 7 days, followed by 5mg twice daily	59/2609	Subcu. enoxaparin followed by warfarin	71/2635	57.0	41.3
Hokusai-VTE	2013	Edoxaban 60mg daily	130/4118	Warfarin	146/4122	55.8	42.7
RE-COVER II	2014	Dabigatran 150mg twice daily	30/1279	Warfarin	28/1289	56.5	39.4

### Publication bias and quality of included studies

To assess potential sources of bias, we used the Cochrane Collaboration’s risk-of-bias tool[33]. We determined that the overall risk of bias in each study was low (S4 File). The risk of selective outcome reporting was determined to be minimal because included trials were Phase 3 randomized trials and included outcomes adjudicated in a blinded fashion.

### Reporting of trial events

Reporting on trial-related efficacy and safety outcomes varied widely among the studies included. All 9 trials provided data on all-cause mortality and the main outcomes for their respective indication, including stroke or systemic embolism, any stroke, hemorrhagic stroke, myocardial infarction, major bleeding, and intracranial bleeding in the NVAF trials and recurrent VTE and related death, recurrent DVT, major bleeding, and fatal bleeding in the VTE trials. Fewer studies reported other efficacy and safety outcomes, especially ADEs such as elevated serum transaminase levels, myocardial infarction, and miscellaneous symptoms such as arthralgia, peripheral edema, or dyspepsia. S5 File lists the data extracted from the 9 included studies.

### Efficacy and safety outcomes in trials of DOACs for prevention of thromboembolism in patients with non-valvular atrial fibrillation

**Fig 2:**
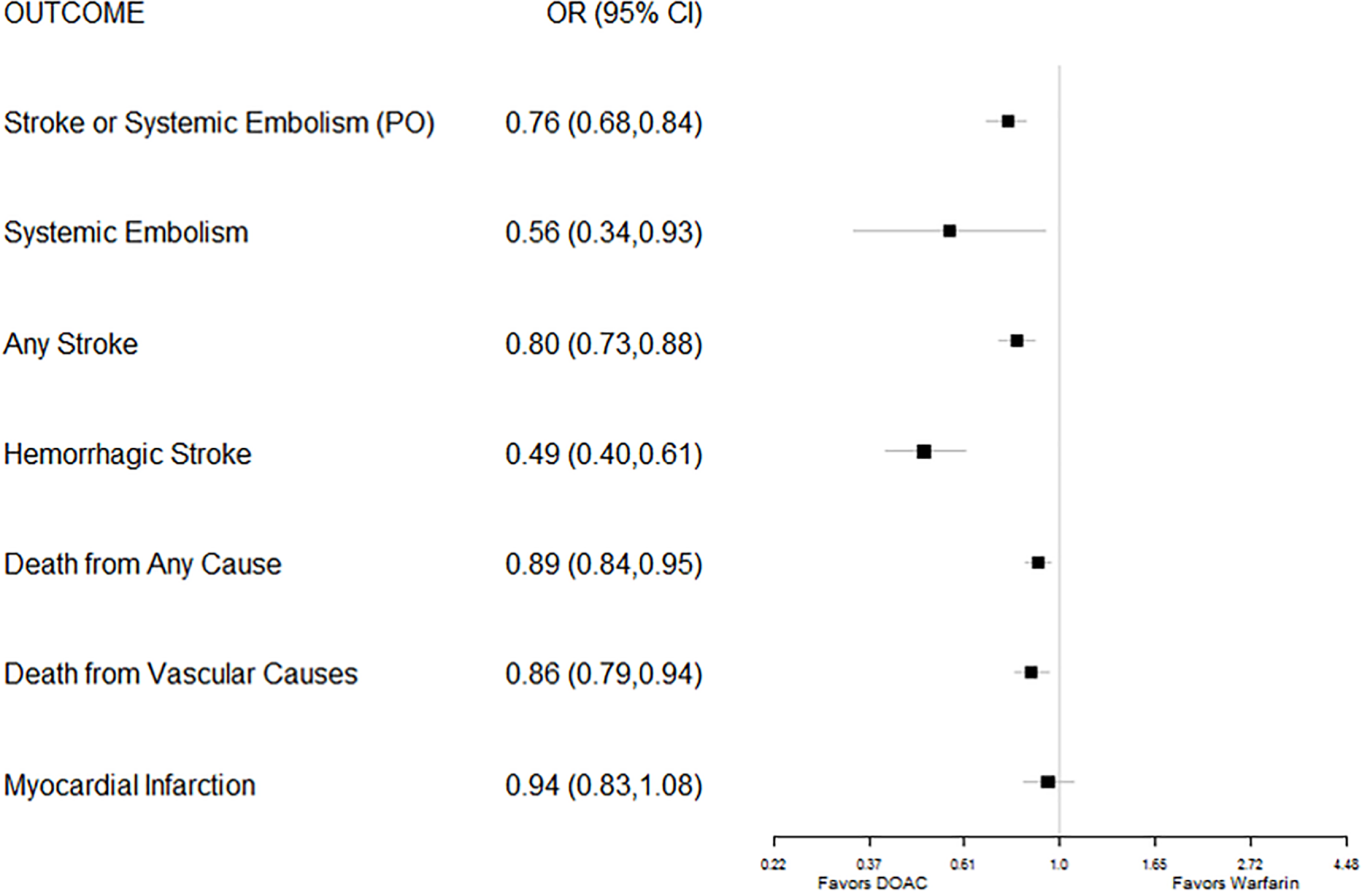
Forest plot of pooled odds ratios (with 95% CI) of various efficacy outcomes for FDA-approved direct oral anticoagulants versus warfarin for thromboembolic stroke prophylaxis in non-valvular atrial fibrillation.

**Fig 3:**
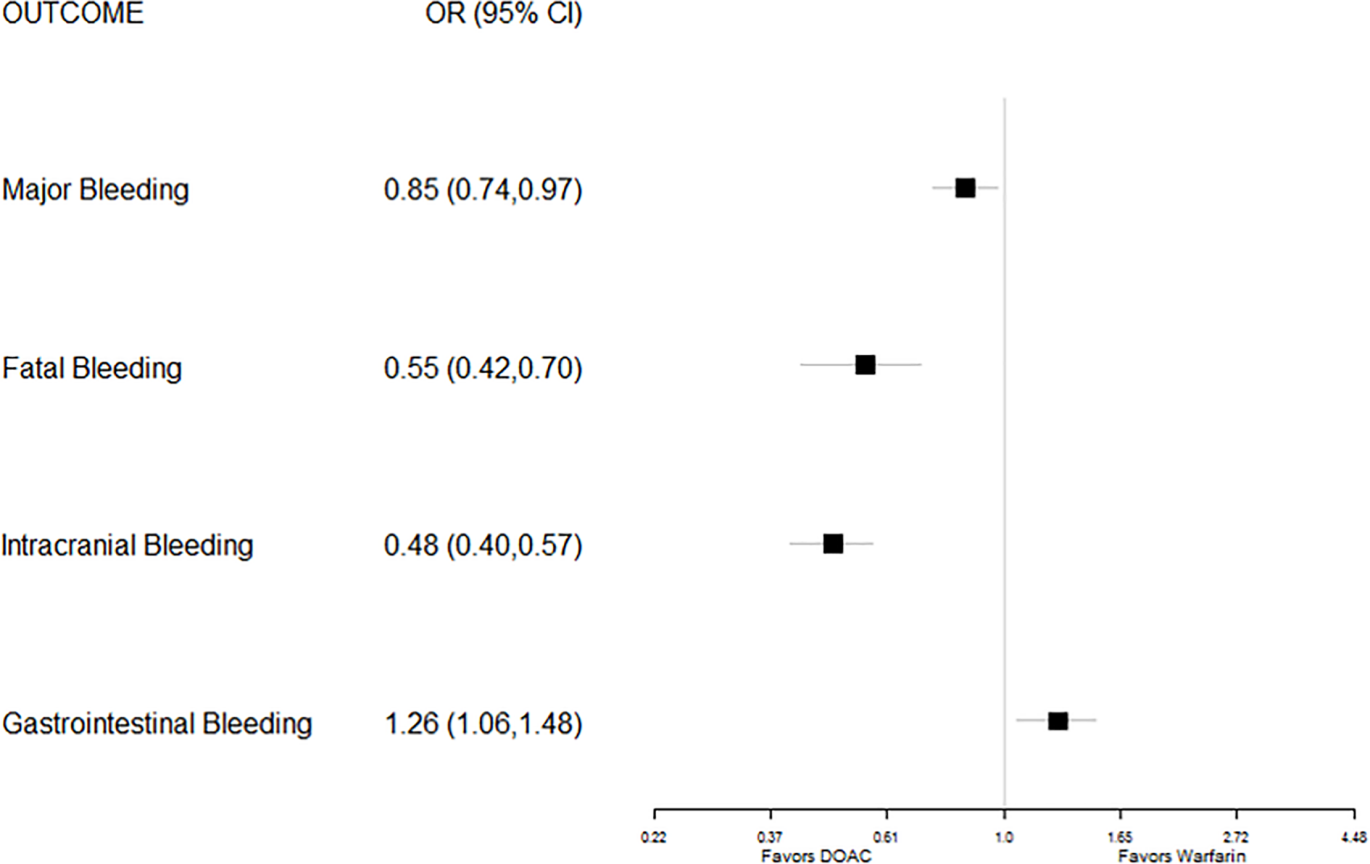
Forest plot of pooled odds ratios (with 95% CI) of various adverse drug events for FDA-approved direct oral anticoagulants versus warfarin for thromboembolic stroke prophylaxis in non-valvular atrial fibrillation.

In trials enrolling participants with NVAF, the odds of developing a stroke or systemic embolism (the primary outcome in most studies of primary prevention in patients with NVAF) was nearly one-quarter lower for patients treated with a DOAC than for those treated with warfarin (pooled odds ratio (OR) 0.76). Patients treated with a DOAC had significantly lower odds of developing any type of stroke (OR 0.80); the greatest benefit came from substantially lower risk of hemorrhagic stroke (OR 0.49). Similarly, the risks of dying from any cause (OR 0.89) and specifically from vascular causes (OR 0.86) were lower for patients treated with a DOAC than for those treated with warfarin (Fig 2). Participants randomized to receive a DOAC also had 15% lower odds of experiencing a major bleeding episode, 45% lower odds of experiencing a fatal bleeding event, and 52% lower odds of experiencing an intracranial bleeding event than participants randomized to receive warfarin therapy. However, participants with NVAF randomized to receive a DOAC had one-quarter higher risk for GI bleeding than participants who were treated with warfarin therapy (Fig 3).

### Efficacy and safety outcomes in trials of DOACs for treatment in patients with acute venous thromboembolism

**Fig 4:**
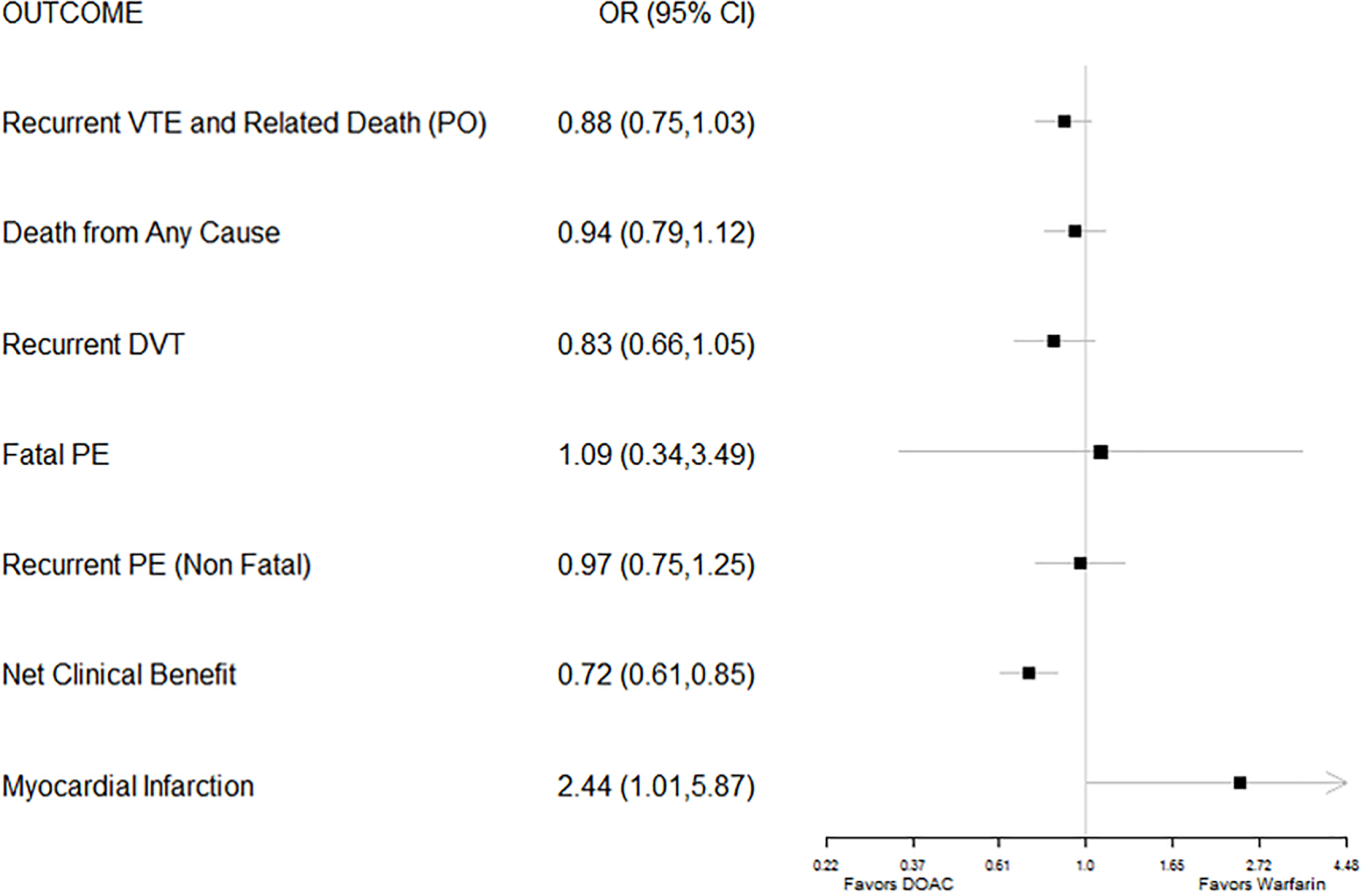
Forest plot of pooled odds ratios (with 95% CI) of various efficacy outcomes for FDA-approved direct oral anticoagulants versus warfarin for treatment of VTE.

In the five Phase 3 studies of DOACs for acute treatment of patients with a DVT, with or without pulmonary embolism (PE), participants randomized to receive a DOAC did not differ from those receiving warfarin on the odds of recurrent VTE and related death (the primary outcome in most studies of secondary prevention in acute VTE), but this primary outcome showed a tendency to favor DOACs (OR 0.88, CI 0.75–1.03). Similarly, the two groups did not differ in their risk of dying from all causes or their risk for a recurrent DVT, fatal PE, or recurrent non-fatal PE (Fig 4).

Recipients of a DOAC also had a favorable net clinical benefit (0.72, 0.61–0.85), but they experienced greater risk of developing a myocardial infarction (OR 2.44, CI 1.01–5.87) than participants randomized to receive warfarin therapy, although the absolute risk was no greater than 0.3%. This relatively higher risk of myocardial infarction persisted when the analysis was restricted to the use of Factor Xa inhibitors alone (OR 3.00, CI 0.81–11.13).

**Fig 5:**
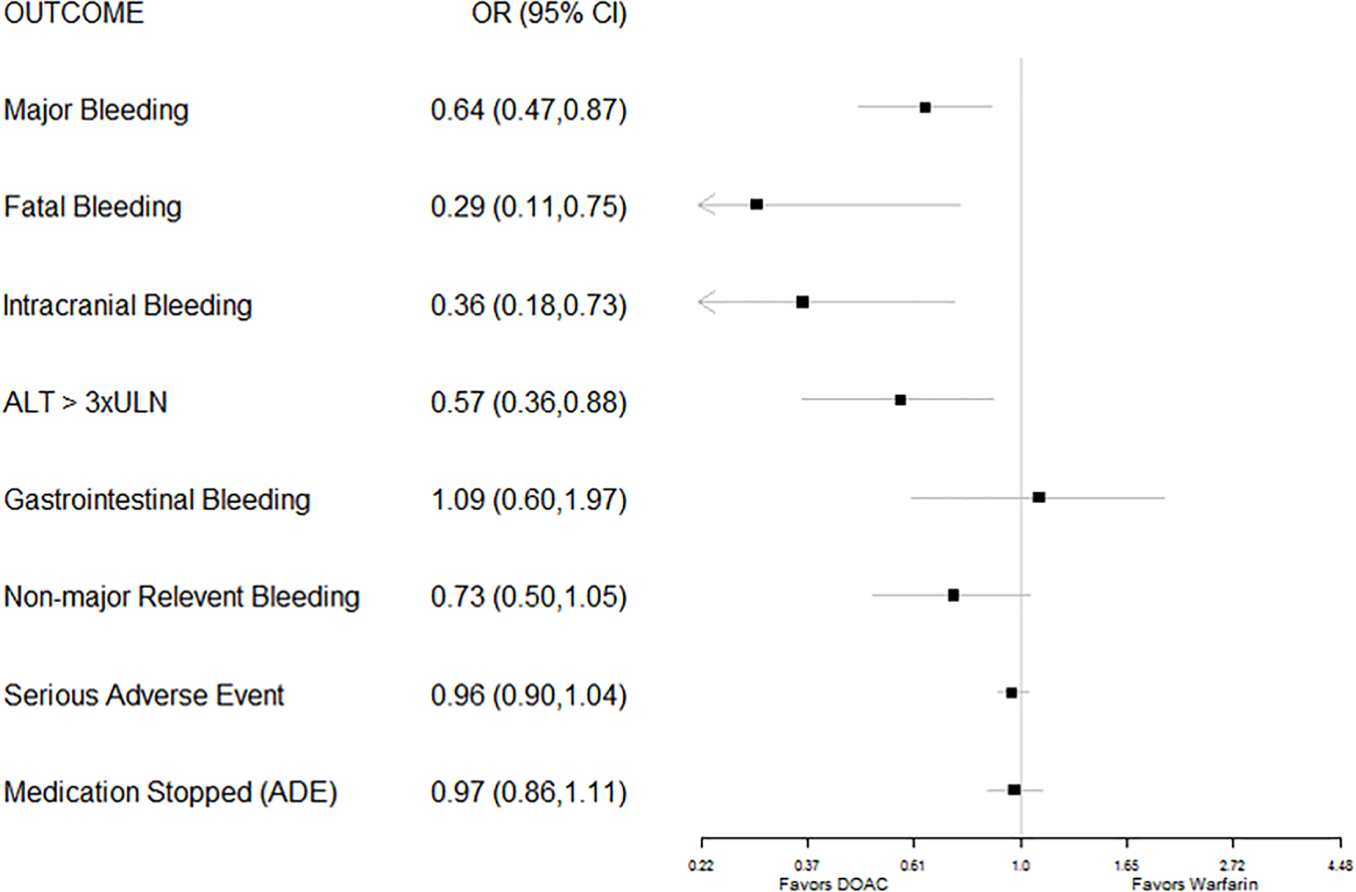
Forest plot of pooled odds ratios (with 95% CI) of various adverse drug events for FDA-approved direct oral anticoagulants versus warfarin for treatment of VTE.

Participants randomized to receive a DOAC had 36% lower odds of an episode of major bleeding, 71% lower odds of fatal bleeding, and 64% lower odds of intracranial bleeding, as well as significantly lower odds of other ADEs such as hepatic transaminase elevations (defined as serum alanine aminotransferase (ALT) level greater than three times the upper limit of normal). In contrast to participants with NVAF, participants treated for an acute VTE with a DOAC did not differ significantly from those treated with warfarin therapy on the odds of experiencing a GI bleed (Fig 5).

## Discussion

This systematic review and meta-analysis provides an updated and pooled analysis of efficacy and safety outcomes related to the use of FDA-approved DOACs (the direct thrombin inhibitor dabigatran and the factor Xa inhibitors apixaban, rivaroxaban, and edoxaban), in comparison with Vitamin K-antagonist-based therapies, for thromboembolic stroke prophylaxis in patients with NVAF and in treatment of patients with acute VTE (DVT with or without PE).

Our analysis showed that patients with NVAF who received one of the 4 approved DOACs for stroke prevention had significantly lower odds of developing a stroke or systemic embolism; in particular, the risk of hemorrhagic stroke was less than half that for those treated with warfarin. Patients on DOACs also experienced significantly lower risk of dying and of developing ADEs, such as a major or fatal bleeding episode, but were at higher risk for GI bleeding than those who were treated with warfarin therapy. In comparison, patients receiving DOACs for management of their acute VTE did not differ significantly from those treated with warfarin in their risk of recurrent VTE and/or death. On the other hand, patients treated with DOACs still experienced a favorable net clinical benefit, primarily because of lower odds of developing all ADEs associated with DOACs. In patients with NVAF, the pooled odds ratio for myocardial infarction was 0.94 (0.83–1.08). Among the four DOACs only dabigatran had an odds ratio for myocardial infarction greater than 1, but the CIs for all four study-level odds ratios included 1, and the pooled odds ratio after excluding dabigatran was 0.88 (0.76–1.02). The available evidence does not establish a difference in the risk of myocardial infarction between the direct thrombin inhibitors and the Factor Xa inhibitors. More generally, useful comparisons of the efficacy and safety profiles among the four DOACs require additional trials, preferably head-to-head comparisons.

Prior systematic reviews and meta-analyses have examined the efficacy and safety of DOACs in patients with various cardiac and non-cardiac conditions. Since the rates of bleeding vary widely between cardiovascular indications and post-operative VTE-prevention indications, however, we did not include trials of patients with non-cardiovascular indications in our systematic review and meta-analysis.

Many of those meta-analyses have only a weak connection to the question that motivated our work. They included data from Phase 2 trials or data for DOACs at dosages not approved by the FDA or for anticoagulants other than DOACs and VKAs, or they combined trials in NVAF or VTE with trials in other cardiovascular conditions, as well as trials in such indications as DVT prophylaxis in hip or knee replacement.

Further, several prior meta-analyses included trials of DOACs for off-label indications, including the prevention of VTE in medically ill patients and treatment of patients with an acute coronary syndrome[34, 35]. Also, several prior meta-analyses included DOACs, such as darexaban and betrixaban, that either have not been approved for use or have been withdrawn from the market. Even among studies that did not have such broader inclusion criteria and related shortcomings, many used methods of analysis that are known to produce unreliable results (S10 File discusses the prior reviews and their limitations). Thus, most prior meta-analyses do not provide usable evidence for the objectives of our systematic review and meta-analysis, even if their results are similar to ours. The main exceptions are two network meta-analyses[36], but they did not produce results for DOACs as a class vs. warfarin.

By including only currently FDA-approved DOACs at FDA-approved doses. using sound analytical methods, and providing a contemporary picture of treatment efficacy and safety, our review and analysis support including DOACs in the clinician’s armamentarium for prevention or treatment of thromboembolic disease in most patients. Further, current practice guidelines for management of anticoagulation in patients with NVAF and VTE[6–10] encourage a shared decision-making process to present personalized risk estimates to individual patients and solicitation of their concerns about treatment prior to recommending long-term preventive therapies. We recommend giving eligible patients and their families information about the risks and benefits of DOACs, along with one’s expert opinion on their expected net clinical benefit, when discussing options for oral anticoagulation treatment.

### Study strengths and limitations

The major strengths of this meta-analysis are its focus on efficacy and safety outcomes related to the use of DOACs in comparison with warfarin-based anticoagulation strategies, its inclusion of all DOACs currently approved for treatment in patients with NVAF and VTE at FDA-approved dosages, its reliance on data from high-quality Phase 3 randomized controlled trials, and its use of sound statistical methods.

Even high-quality Phase 3 trials, however, can differ in ways that add uncertainty to the results and interpretation of a meta-analysis. For example, in the NVAF trials, patients who were treated with warfarin therapy in the ROCKET AF trial had an INR in the therapeutic range (2.0–3.0) for a smaller percentage of the time (median 58%) than those in the ARISTOTLE, ENGAGE AF-TIMI 48, and RE-LY trials. Trial inclusion and exclusion criteria can also create disparities and confusion in data interpretation. In making indirect comparisons among apixaban, dabigatran, and rivaroxaban in NVAF, Schneeweiss et al.[37] focused on the baseline CHADS_2_ score in the warfarin control groups: 87% of patients in the ROCKET AF trial had scores ≥3, versus 30% in the ARISTOTLE trial and 32% in the RE-LY trial. The investigators dealt with these important discrepancies by using data for the subset of patients in the ARISTOTLE and RE-LY trials with CHADS_2_ score ≥3. This type of departure from comparability may have less impact on pairwise meta-analysis than on indirect comparisons, but it would be useful to carry out a meta-analysis for the subset of patients in all 4 NVAF trials with CHADS_2_ scores ≥3. As far as we are aware, however, these data for the ARISTOTLE, ENGAGE AF-TIMI 48, and RE-LY trials are not publicly available.

Several DOACs either have been withdrawn from the market or were not approved for use at certain doses because of their high risk-to-benefit ratio or ADEs observed in preliminary trials. Use of data from these studies would probably have led to overestimation of ADE risks for DOACs. Finally, the population of participants in the EINSTEIN-PE trial[38] differed sufficiently from those in the 5 VTE trials that we excluded this study from the current analysis.

Although we have included data from all relevant Phase 3 randomized trials published to date, the number of trials is modest; results from ongoing trials and post-market surveillance studies should be considered as such data become available.

## Conclusions

In our contemporary analysis including all Phase 3 clinical trials examining the efficacy and safety of DOACs, particularly when used for FDA-approved cardiovascular indications and dosages, DOACs as a class were associated with a superior, or at least comparable, efficacy profile and with substantially lower odds of bleeding complications than warfarin-based anticoagulation strategies. Participants treated with DOACs also experienced either comparable or lower odds of dying from all causes, depending on the indication. With the exception of higher odds of GI bleeding among trial participants with NVAF who received rivaroxaban or dabigatran, the overall safety profile of DOACs was favorable in comparison with warfarin. We therefore conclude that DOACs should be considered as safe and effective alternatives to warfarin therapy in patients with NVAF and VTE.

## Supporting information

S1 File PRISMA checklist. (DOC)

S2 File Search protocol. (PDF)

S3 File Articles excluded after review of full text. (PDF)

S4 File Risk-of-bias assessment. (PDF)

S5 File Data extraction (data abstraction forms and extracted data). (PDF)

S6 File Choice of measure of effect. (PDF)

S7 File Meta-analysis methods. (PDF)

S8 File Details of models used for meta-analysis. (PDF)

S9 File Results of meta-analyses. (PDF)

S10 File Previous meta-analyses. (DOCX)

## Abbreviations

ADE: Adverse drug event
CI: Confidence interval
DOAC: Direct oral anticoagulant
DVT: Deep venous thrombosis
GI: Gastrointestinal
INR: International normalized ratio
NVAF: Non-valvular atrial fibrillation
OR: Odds ratio
PE: Pulmonary embolus
PRISMA: Preferred Reporting Items for Systematic Reviews and Meta-Analyses
VKA: Vitamin K antagonist
VTE: Venous thromboembolism
