# Supplementary material for: Efficacy and safety of direct oral anticoagulants approved for cardiovascular indications: Systematic review and meta-analysis
Source: PLoS One. 2018 May 24;13(5):e0197583. doi: 10.1371/journal.pone.0197583 (PMC5967718; doi:10.1371/journal.pone.0197583)
Supplement: S2 File — (PDF) [file pone.0197583.s002.pdf]

Search Protocol

Supplement to: Makam RCP, Hoaglin DC, McManus DD, Wang V, Gore JM, Spencer FA, Pradhan R, Tran H, Yu H, Goldberg RJ. Efficacy and safety of direct oral anticoagulants approved for cardiovascular indications: systematic review and meta-analysis.

Objective

To investigate the safety of direct oral anticoagulants (DOACs) as a therapeutic class when used for on-label cardiovascular indications.

Criteria for considering studies for this review

We will include evidence from phase 3 randomized clinical trials that compared any of the DOACs at standard dosages to warfarin for the prevention of thromboembolism in patients with non-valvular atrial fibrillation (NVAF) or for the treatment of acute venous thromboembolism (VTE). All articles must be published in a peer-reviewed journals and written in English to be considered for inclusion. We excluded all other type of study rather than phase 3 randomized clinical trial. We also excluded trials that investigated the efficacy and safety of DOACs for other indications (e.g., prophylaxis in patients undergoing hip and knee surgery or prevention of VTE in medically ill patients). We also excluded trials for FDA-approved indications, but with dosages other than those approved by the FDA.

Included DOACs:

Apixaban
Dabigatran
Rivaroxaban
Edoxaban

Types of outcomes:

All-cause mortality
Major bleeding
Fatal bleeding
Non-major relevant bleeding
Elevated liver function test > 3 fold

Search methods for identification of studies

Electronic searches

We performed a search of PubMed (which includes MEDLINE), Scopus (which includes EMBASE), and Cochrane databases. We also reviewed the references of articles that meet selection criteria to be included for abstraction Google Scholar and citation of previously-published reviews.

Dates Searched:

From inception until July 25th, 2016

Search terms:

PubMed:
((("apixaban"[MeSH Terms] OR "apixaban"[All Fields]) OR ("dabigatran"[MeSH Terms] OR "dabigatran"[All Fields]) OR ("rivaroxaban"[MeSH Terms] OR "rivaroxaban"[All Fields]) OR ("edoxaban"[MeSH Terms] OR "edoxaban"[All Fields]) OR ("darexaban"[MeSH Terms] OR "darexaban"[All Fields]) OR "novel oral anticoagulants"[All Fields] OR "non-vitamin K antagonist oral anticoagulants"[All Fields]) AND English[lang]) NOT ((("ximelagatran"[Supplementary Concept] OR "ximelagatran"[MeSH Terms] OR "ximelagatran"[All Fields]) AND Filters[All Fields]) AND ("clinical trial"[Publication Type] OR "clinical trials as topic"[MeSH Terms] OR "clinical trial"[All Fields])) AND (Clinical Trial[ptyp] AND "humans"[MeSH Terms] AND English[lang])

Scopus
(TITLE-ABS-KEY(apixaban) OR TITLE-ABS-KEY(dabigatran) OR TITLE-ABS-KEY(rivaroxaban) OR TITLE-ABS-KEY(edoxaban) OR TITLE-ABS-KEY(darexaban) OR TITLE-ABS-KEY(novel oral anticoagulants) OR TITLE-ABS-KEY(novel oral anticoagulant) OR TITLE-ABS-KEY(non-vitamin K antagonist oral anticoagulants) OR TITLE-ABS-KEY(non-vitamin K antagonist oral anticoagulant) AND NOT TITLE-ABS-KEY(ximelagatran)) AND DOCTYPE(ar) AND NOT(Review) AND NOT(meta-analysis) AND NOT(metaanalysis) AND NOT(mice) AND NOT(rat) AND (LIMIT-TO(LANGUAGE,"English"))

Cochrane:
'Apixaban OR Dabigatran OR Rivaroxaban OR Edoxaban OR Darexaban in Title, Abstract, Keywords in Trials'

Google Scholar:
(Apixaban or dabigatran or rivaroxaban or edoxaban or darexaban) and (clinical trial)
We searched Google Scholar for gray literature in the first 15 pages of results after the final list from three databases have been identified.

Data selection process

After searches have been performed in three databases, references and abstracts were imported into EndNote X7 Reference Manager software. We removed duplicates which matched on author name, year of publication, and title. We reviewed the titles and removed references whose titles indicating other type of study (e.g., meta-analysis, systematic review, letter to the editor, abstract, phase 1 or phase 2 clinical trial, secondary analysis or subgroup analysis of trials, phase 3 trial testing medications other than DOACs, phase 3 trial comparing DOACs with aspirin,

study with outcome other than safety and efficacy [e.g., cost-effective analysis, bioavailability], and study in animals). The remaining references were further reviewed for their abstracts. We excluded references while reviewing abstracts with similar criteria with title exclusion, and additional duplicates which were not found during prior process. Relevant references were reviewed for their full text for inclusion consideration. The final pool of articles included 9 large phase 3 randomized clinical trials, from which we extracted data for analysis.

Librarian Reviewers

Judith Nordberg, University of Massachusetts Medical School
Rebecca Reznik-Zellen, University of Massachusetts Medical School

Inclusion criteria

1. Trials comparing any of the **approved DOACs** (dabigatran, apixaban, rivaroxaban and edoxaban) with conventionally used anticoagulants like warfarin AND used for approved **cardiovascular indications** (stroke prevention in non-valvular atrial fibrillation, acute and post-acute management of venous thrombo-embolism (pulmonary embolus or deep vein thrombosis)
2. Randomized clinical trials
3. Phase 3 or higher
4. Dosages used were similar to those for approved indications
5. ADEs were reported

Exclusion criteria

1. Non-approved DOACs (darexaban, betrixaban, letaxaban, ximelagatran, etc.)
2. Non-approved indications (myocardial infarction, stroke, medically ill, thrombophilia, etc.)
3. Non-cardiovascular indications (thromboprophylaxis in hip and knee surgeries)
4. Phase 2 trials, experimental drugs and/or indications
5. Intensity of ADEs were not mentioned
6. Non-English publications, poor study design, or unpublished/non-peer-reviewed papers
