# Supplementary material for: Efficacy and safety of direct oral anticoagulants approved for cardiovascular indications: Systematic review and meta-analysis
Source: PLoS One. 2018 May 24;13(5):e0197583. doi: 10.1371/journal.pone.0197583 (PMC5967718; doi:10.1371/journal.pone.0197583)
Supplement: S3 File — (PDF) [file pone.0197583.s003.pdf]

Articles Excluded after Review of Full Text

Supplement to: Makam RCP, Hoaglin DC, McManus DD, Wang V, Gore JM, Spencer FA, Pradhan R, Tran H, Yu H, Goldberg RJ. Efficacy and safety of direct oral anticoagulants approved for cardiovascular indications: systematic review and meta-analysis.

Non-clinical-trial studies[1-8]

1. Efremidis M, Vlachos K, Letsas KP, et al. Low dose dabigatran versus uninterrupted acenocoumarol for peri-procedural anticoagulation in atrial fibrillation catheter ablation. Journal of electrocardiology 2015;48:840-4.
2. Mao L, Li C, Li T, Yuan K. Prevention of stroke and systemic embolism with rivaroxaban compared with warfarin in Chinese patients with atrial fibrillation. Vascular 2014;22:252-8.
3. Rosencher N, Llau J, Mueck W, Loewe A, Berkowitz S, Homering M. Incidence of neuraxial haematoma after total hip or knee surgery: RECORD programme (rivaroxaban vs. enoxaparin). Acta Anaesthesiologica Scandinavica 2013;57:565-72.
4. Hori M, Matsumoto M, Tanahashi N, et al. Rivaroxaban vs. warfarin in Japanese patients with atrial fibrillation–the J-ROCKET AF study–. Circulation journal: official journal of the Japanese Circulation Society 2011;76:2104-11.
5. Paikin JS, Haroun MJ, Eikelboom JW. Dabigatran for stroke prevention in atrial fibrillation: the RE-LY trial. Expert review of cardiovascular therapy 2011;9:279-86.
6. Romualdi E, Donadini MP, Ageno W. Oral rivaroxaban after symptomatic venous thromboembolism: the continued treatment study (EINSTEIN-extension study). Expert review of cardiovascular therapy 2011;9:841-4.
7. Fuji T, Fuijita S, Ujihira T, Sato T. Dabigatran etexilate prevents venous thromboembolism after total knee arthroplasty in Japanese patients with a safety profile comparable to placebo. The Journal of arthroplasty 2010;25:1267-74.
8. Haines DE, Mead-Salley M, Salazar M, et al. Dabigatran versus warfarin anticoagulation before and after catheter ablation for the treatment of atrial fibrillation. Journal of Interventional Cardiac Electrophysiology 2013;37:233-9.

Secondary analysis[1-5]

1. O'Donoghue ML, Ruff CT, Giugliano RP, et al. Edoxaban vs. warfarin in vitamin K antagonist experienced and naive patients with atrial fibrillation. European heart journal 2015:ehv014.
2. Hylek EM, Held C, Alexander JH, et al. Major bleeding in patients with atrial fibrillation receiving apixaban or warfarin: The ARISTOTLE Trial (Apixaban for Reduction in Stroke and Other Thromboembolic Events in Atrial Fibrillation): Predictors, Characteristics, and Clinical Outcomes. Journal of the American College of Cardiology 2014;63:2141-7.
3. Schulman S, Kakkar AK, Goldhaber SZ, et al. Treatment of acute venous thromboembolism with dabigatran or warfarin and pooled analysis. Circulation 2014:CIRCULATIONAHA. 113.004450.
4. Agnelli G, Buller HR, Cohen A, et al. Apixaban for extended treatment of venous thromboembolism. New England Journal of Medicine 2013;368:699-708.
5. Schulman S, Kearon C, Kakkar AK, et al. Extended use of dabigatran, warfarin, or placebo in venous thromboembolism. New England Journal of Medicine 2013;368:709-18.

Non-English full text [1,2]

1. De Alba DA. Randomized clinical trial of rivaroxaban in the prevention of post-thrombotic syndrome | Ensayo clínico aleatorizado de rivaroxaban en la prevención del síndrome postrombótico. Revista Mexicana de Angiología 2015;43:109-15.
2. Di Pasquale G, Riva L. Edoxaban in atrial fibrillation: The ENGAGE AF-TIMI 48 trial. Giornale Italiano di Cardiologia 2014;15:22S-6S.

Other indications

1. Raskob GE, Spyropoulos AC, Zrubek J, et al. The MARINER trial of rivaroxaban after hospital discharge for medical patients at high risk of VTE. Thrombosis and haemostasis 2016;115:1240-8.
2. Kuwahara T, Abe M, Yamaki M, et al. Apixaban versus Warfarin for the prevention of periprocedural cerebral Thromboembolism in atrial fibrillation ablation: multicenter prospective randomized study. Journal of cardiovascular electrophysiology 2016.
3. Cavender MA, Gibson CM, Braunwald E, et al. The effect of rivaroxaban on myocardial infarction in the ATLAS ACS 2-TIMI 51 trial. European Heart Journal: Acute Cardiovascular Care 2015;4:468-74.
4. Cappato R, Marchlinski FE, Hohnloser SH, et al. Uninterrupted rivaroxaban vs. uninterrupted vitamin K antagonists for catheter ablation in non-valvular atrial fibrillation. European heart journal 2015;36:1805-11.
5. Zou Y, Tian S, Wang Y, Sun K. Administering aspirin, rivaroxaban and low-molecular-weight heparin to prevent deep venous thrombosis after total knee arthroplasty. Blood Coagulation & Fibrinolysis 2014;25:660-4.
6. Mirdamadi A, Dashtkar S, Kaji M, Pazhang F, Haghpanah B, Gharipour M. Dabigatran versus Enoxaparin in the prevention of venous thromboembolism after total knee arthroplasty: A randomized clinical trial. ARYA atherosclerosis 2014;10:292.
7. Cappato R, Ezekowitz MD, Klein AL, et al. Rivaroxaban vs. vitamin K antagonists for cardioversion in atrial fibrillation. European heart journal 2014;35:3346-55.
8. Mega JL, Braunwald E, Wiviott SD, et al. Comparison of the Efficacy and Safety of Two Rivaroxaban Doses in Acute Coronary Syndrome (from ATLAS ACS 2–TIMI 51). The American journal of cardiology 2013;112:472-8.
9. Maddox W, Kay G, Yamada T, et al. Dabigatran versus warfarin therapy for uninterrupted oral anticoagulation during atrial fibrillation ablation. Journal of cardiovascular electrophysiology 2013;24:861-5.
10. Gibson CM, Chakrabarti AK, Mega J, et al. Reduction of stent thrombosis in patients with acute coronary syndromes treated with rivaroxaban in ATLAS-ACS 2 TIMI 51. Journal of the American College of Cardiology 2013;62:286-90.
11. Cohen AT, Spiro TE, Büller HR, et al. Rivaroxaban for thromboprophylaxis in acutely ill medical patients. New England Journal of Medicine 2013;368:513-23.
12. Mega JL, Braunwald E, Wiviott SD, et al. Rivaroxaban in patients with a recent acute coronary syndrome. New England Journal of Medicine 2012;366:9-19.
13. Kaseno K, Naito S, Nakamura K, et al. Efficacy and safety of periprocedural dabigatran in patients undergoing catheter ablation of atrial fibrillation. Circulation journal: official journal of the Japanese Circulation Society 2011;76:2337-42.
14. Goldhaber SZ, Leizorovicz A, Kakkar AK, et al. Apixaban versus enoxaparin for thromboprophylaxis in medically ill patients. New England Journal of Medicine 2011;365:2167-77.
15. Alexander JH, Lopes RD, James S, et al. Apixaban with antiplatelet therapy after acute coronary syndrome. New England Journal of Medicine 2011;365:699-708.
16. Lassen MR, Raskob GE, Gallus A, Pineo G, Chen D, Hornick P. Apixaban versus enoxaparin for thromboprophylaxis after knee replacement (ADVANCE-2): a randomised double-blind trial. The Lancet 2010;375:807-15.
17. Lassen MR, Gallus A, Raskob GE, Pineo G, Chen D, Ramirez LM. Apixaban versus enoxaparin for thromboprophylaxis after hip replacement. New England Journal of Medicine 2010;363:2487-98.

18. Turpie AG, Lassen MR, Davidson BL, et al. Rivaroxaban versus enoxaparin for thromboprophylaxis after total knee arthroplasty (RECORD4): a randomised trial. The Lancet 2009;373:1673-80.
19. Lassen MR, Raskob GE, Gallus A, Pineo G, Chen D, Portman RJ. Apixaban or enoxaparin for thromboprophylaxis after knee replacement. New England Journal of Medicine 2009;361:594-604.
20. Ginsberg J, Davidson B, Comp P, et al. Oral thrombin inhibitor dabigatran etexilate vs North American enoxaparin regimen for prevention of venous thromboembolism after knee arthroplasty surgery. The Journal of arthroplasty 2009;24:1-9.
21. Lassen MR, Ageno W, Borris LC, et al. Rivaroxaban versus enoxaparin for thromboprophylaxis after total knee arthroplasty. New England Journal of Medicine 2008;358:2776-86.
22. Kakkar AK, Brenner B, Dahl OE, et al. Extended duration rivaroxaban versus short-term enoxaparin for the prevention of venous thromboembolism after total hip arthroplasty: a double-blind, randomised controlled trial. The Lancet 2008;372:31-9.
23. Eriksson BI, Borris LC, Friedman RJ, et al. Rivaroxaban versus enoxaparin for thromboprophylaxis after hip arthroplasty. New England Journal of Medicine 2008;358:2765-75.
24. Eriksson BI, Dahl OE, Rosencher N, et al. Dabigatran etexilate versus enoxaparin for prevention of venous thromboembolism after total hip replacement: a randomised, double-blind, non-inferiority trial. The Lancet 2007;370:949-56.
25. Eriksson B, Dahl O, Rosencher N, et al. Oral dabigatran etexilate vs. subcutaneous enoxaparin for the prevention of venous thromboembolism after total knee replacement: the RE-MODEL randomized trial. Journal of Thrombosis and Haemostasis 2007;5:2178-85.
26. Eriksson BI, Dahl O, Büller H, et al. A new oral direct thrombin inhibitor, dabigatran etexilate, compared with enoxaparin for prevention of thromboembolic events following total hip or knee replacement: the BISTRO II randomized trial. Journal of Thrombosis and Haemostasis 2005;3:103-11.
27. Du W, Zhao C, Wang J, Liu J, Shen B, Zheng Y. Comparison of rivaroxaban and parnaparin for preventing venous thromboembolism after lumbar spine surgery. Journal of orthopaedic surgery and research 2015;10:78.

Non-warfarin control[1-5]

1. Fuji T, Wang C-J, Fujita S, et al. Safety and efficacy of edoxaban, an oral factor Xa inhibitor, versus enoxaparin for thromboprophylaxis after total knee arthroplasty: the STARS E-3 trial. Thrombosis research 2014;134:1198-204.
2. Flaker GC, Eikelboom JW, Shestakovska O, et al. Bleeding during treatment with aspirin versus apixaban in patients with atrial fibrillation unsuitable for warfarin. Stroke 2012;43:3291-7.
3. Sakon M, Nakamura M. Darexaban (YM150) prevents venous thromboembolism in Japanese patients undergoing major abdominal surgery: Phase III randomized, mechanical prophylaxis-controlled, open-label study. Thrombosis research 2012;130:e52-e9.
4. Connolly SJ, Eikelboom J, Joyner C, et al. Apixaban in patients with atrial fibrillation. New England Journal of Medicine 2011;364:806-17.
5. Oldgren J, Budaj A, Granger CB, et al. Dabigatran vs. placebo in patients with acute coronary syndromes on dual antiplatelet therapy: a randomized, double-blind, phase II trial. European heart journal 2011;32:2781-9.

Sample size not reported/small[1-3]

1. Nakamura M, Nishikawa M, Komuro I, et al. Apixaban for the treatment of Japanese subjects with acute venous thromboembolism (AMPLIFY-J Study). Circ J 2015;79:1230-6.
2. Yamada N, Hirayama A, Maeda H, et al. Oral rivaroxaban for Japanese patients with symptomatic venous thromboembolism–the J-EINSTEIN DVT and PE program. Thrombosis journal 2015;13:2.
3. Eriksson BI, Dahl OE, Huo MH, et al. Oral dabigatran versus enoxaparin for thromboprophylaxis after primary total hip arthroplasty (RE-NOVATE II). Thromb Haemost 2011;105:721-9.
