# Supplementary material for: Efficacy and safety of direct oral anticoagulants approved for cardiovascular indications: Systematic review and meta-analysis
Source: PLoS One. 2018 May 24;13(5):e0197583. doi: 10.1371/journal.pone.0197583 (PMC5967718; doi:10.1371/journal.pone.0197583)
Supplement: S4 File — (PDF) [file pone.0197583.s004.pdf]

Risk-of-bias Assessment

Supplement to: Makam RCP, Hoaglin DC, McManus DD, Wang V, Gore JM, Spencer FA, Pradhan R, Tran H, Yu H, Goldberg RJ. Efficacy and safety of direct oral anticoagulants approved for cardiovascular indications: systematic review and meta-analysis.

	NVAF Studies				VTE Studies				
	ARISTOTLE	RE-LY 150	ROCKET AF	ENGAGE AF	AMPLIFY	EINSTEIN-DVT	Hokusai-VTE	RE-COVER	RECOVER-2
Random sequence generation (selection bias)	Low risk	Low risk	Low risk	Low risk	Low risk	Low risk	Low risk	Low risk	Low risk
Allocation concealment (selection bias)	Low risk	Low risk	Low risk	Low risk	Low risk	Low risk	Low risk	Low risk	Low risk
Blinding of participants and personnel (performance bias)	Low risk	Unclear risk	Low risk	Low risk	Low risk	Unclear risk	Low risk	Low risk	Low risk
Blinding of outcome assessment (detection bias) (patient-reported outcomes)	Low risk	Unclear risk	Low risk	Low risk	Low risk	Unclear risk	Low risk	Low risk	Low risk
Blinding of outcome assessment (detection bias) (Mortality)	Low risk	Unclear risk	Low risk	Low risk	Low risk	Unclear risk	Low risk	Low risk	Low risk
Incomplete outcome data addressed (attrition bias)	Low risk	Unclear risk	Low risk	Low risk	Low risk	Low risk	Unclear risk	Unclear risk	Low risk
Selective reporting (reporting bias)	Low risk	Unclear risk	Low risk	Low risk	Low risk	Low risk	Low risk	Low risk	Low risk
**Summary Judgment**	**Low risk**	**Unclear risk**	**Low risk**	**Low risk**	**Low risk**	**Unclear risk**	**Low risk**	**Low risk**	**Low risk**
