# Supplementary material for: Efficacy and safety of direct oral anticoagulants approved for cardiovascular indications: Systematic review and meta-analysis
Source: PLoS One. 2018 May 24;13(5):e0197583. doi: 10.1371/journal.pone.0197583 (PMC5967718; doi:10.1371/journal.pone.0197583)
Supplement: S5 File — (PDF) [file pone.0197583.s005.pdf]

Data Extraction

Supplement to: Makam RCP, Hoaglin DC, McManus DD, Wang V, Gore JM, Spencer FA, Pradhan R, Tran H, Yu H, Goldberg RJ. Efficacy and safety of direct oral anticoagulants approved for cardiovascular indications: systematic review and meta-analysis.

Efficacy Data Abstraction Form

RCT: _________ **Year: ______**

Efficacy outcomes

Death from any cause

Death from vascular causes

Death related to pulmonary embolism

Death where pulmonary embolism could not be ruled out

Any Stroke

Hemorrhagic stroke

Ischemic stroke

Stroke or systemic embolism

Systemic embolism

Myocardial infarction

Fatal pulmonary embolism

Nonfatal pulmonary embolism

Recurrent pulmonary embolism (fatal/non-fatal)

Recurrent pulmonary embolism (non fatal)

Venous thromboembolism or related death during the study period

Recurrent Venous thromboembolism and related death

Symptomatic deep-vein thrombosis

Recurrent deep-vein thrombosis

Net clinical benefit in terms of Venous thromboembolism plus major bleeding

Efficacy Data Extracted: Non-valvular atrial fibrillation

	ARISTOTLE	
	Apixaban (N = 9120)	Warfarin (N = 9081)
Stroke or systemic embolism	212	265
Any Stroke	199	250
Ischemic or uncertain type of stroke	162	175
Hemorrhagic stroke	40	78
Systemic embolism	15	17
Death from any cause	603	669
Stroke, systemic embolism, or death from any cause	752	837
Myocardial infarction	90	102
Stroke, systemic embolism, myocardial infarction, or death from any cause	810	906
Pulmonary embolism or deep-vein thrombosis	7	9

	RE-LY – 150	
	Dabigatran, 150 mg (N = 6076)	Warfarin (N = 6022)
Stroke or systemic embolism	134	199
Any Stroke	122	185
Ischemic or uncertain type of stroke	111	142
Hemorrhagic stroke	12	45
Death from any cause	438	487
Myocardial infarction	89	63
Nondisabling stroke	44	69
Disabling or fatal stroke	80	118
Pulmonary embolism	18	11
Hospitalization	2439	2458
Death from vascular causes	274	317

	ROCKET AF	
	Rivaroxaban (N=6958)	Warfarin (N=7004)
Stroke or systemic embolism	188	241
Any Stroke	184	221
Ischemic or uncertain type of stroke		
Hemorrhagic stroke	29	50
Systemic embolism	5	22
Death from any cause	208	250
Myocardial infarction	101	126
Nondisabling stroke	88	87
Death from vascular causes	170	193
Stroke, non-CNS embolism, and vascular death	346	410
Stroke, non-CNS embolism, vascular death, and myocardial infarction	433	519
Fatal stroke	47	67
Disabling stroke	43	57
Ischemic stroke	149	161

	ENGAGE-AF	
	Edoxaban (N = 7035)	Warfarin (N=7036)
Stroke or systemic embolism	182	232
Any Stroke	281	317
Ischemic or uncertain type of stroke	236	235
Hemorrhagic stroke	49	90
Systemic embolism	15	23
Death from any cause	773	839
Stroke, systemic embolism, or death from any cause	949	1046
Myocardial infarction	133	141
Disabling or fatal stroke	132	135
Death from vascular causes	530	611
Stroke, non-CNS embolism, and vascular death	728	831
Stroke, non-CNS embolism, vascular death, and myocardial infarction	827	926
Fatal stroke	80	86
Nondisabling and nonfatal stroke	154	190
Stroke, systemic embolic event, or death from cardiovascular causes	728	831
Death from cardiovascular causes	530	611
Ischemic stroke	236	235
Death or disabling stroke	812	878
Death or intracranial hemorrhage	817	926

Efficacy Data Extracted: Venous Thromboembolism

	RE-COVER	
	Dabigatran (N=1274)	Warfarin (N = 1265)
Venous thromboembolism or related death during the study period	30	27
Venous thromboembolism or related death during the study period plus an additional 30-day follow-up	34	32
Symptomatic deep-vein thrombosis	16	18
Symptomatic nonfatal pulmonary embolism	13	7
Death related to venous thromboembolism	1	3
All deaths	21	21
Myocardial infarction	4	2

	RE-COVER II	
	Dabigatran (N=1279)	Warfarin (n=1289)
Venous thromboembolism or related death during the study period	30	28
Venous thromboembolism or related death during the study period plus an additional 30-day follow-up	34	30
Symptomatic deep-vein thrombosis	25	17
Symptomatic nonfatal pulmonary embolism	7	13
All deaths	25	25
Death related to pulmonary embolism	3	0
Myocardial infarction	4	2

	EINSTEIN DVT	
	Rivaroxaban (ITT N=1731)	Enoxaparin–VKA (ITT N=1718)
Symptomatic deep-vein thrombosis	14	28
Symptomatic nonfatal pulmonary embolism	20	18
All deaths	38	49
Death related to pulmonary embolism	4	6
Recurrent VTE	36	51
Fatal PE	1	0
Nonfatal PE	20	18
Recurrent DVT plus PE	1	0
Recurrent DVT	14	28
Net clinical benefit in terms of VTE plus major bleeding	51	73
Myocardial infarction	5	1
Ischemic stroke	3	5

	AMPLIFY	
	Apixaban (N = 2609)	Conventional Therapy (N = 2635)
Venous thromboembolism or related death during the study period	59	71
All deaths	41 out of 2676	52 out of 2689
Fatal PE	1	2
Death where PE could not be ruled out	11	13
Nonfatal PE	27	23
Recurrent DVT	20	33
Net clinical benefit in terms of VTE plus major bleeding	73	118
VTE or death from cardiovascular cause	61	77
VTE or death from any cause	84	104
VTE, VTE-related death, or major bleeding	73	118
Myocardial infarction	4	2
Ischemic stroke	6	3
Hemorrhagic stroke	2	5

	Hokusai-VTE	
	Edoxaban (N= 4118)	Warfarin (N = 4122)
Venous thromboembolism or related death during the study period	130	146
All deaths	132	126
Fatal PE	4	3
Death where PE could not be ruled out	20	21
Nonfatal PE	49	59
Recurrent DVT	57	63
Net clinical benefit in terms of VTE plus major bleeding	120	144
VTE or death from any cause	228	228
Myocardial infarction	20	13
Ischemic stroke	26	26

Safety Data Abstraction Form

RCT: _________ **Year: ______**

Adverse Drug Events

Major bleeding

Life threatening

Non–life threatening

Gastrointestinal

Intracranial bleeding

Extracranial bleeding

Minor bleeding

Major or minor bleeding

Net clinical benefit outcome

Other Adverse events

Dyspepsia

Dizziness

Dyspnea

Peripheral edema

Fatigue

Cough

Chest pain

Back pain 316

Arthralgia

Nasopharyngitis

Diarrhea

Atrial fibrillation

Urinary tract infection

Upper respiratory tract infection

Liver function

ALT or AST >3× ULN

ALT or AST >3× ULN with concurrent bilirubin >2× ULN

Hepatobiliary disorder

Serious adverse event

Non–serious adverse event

Any other ADEs not listed above_____

Study-drug discontinuation

Discontinued at 1 yr

Discontinued at 2 yr

Reason for discontinuation

Patient's decision

Outcome event

Serious adverse event

Gastrointestinal symptoms

Gastrointestinal bleeding

Safety Data Extracted: Non-valvular atrial fibrillation

	ARISTOTLE	
	Apixaban (N= 9088)	Warfarin (N=9052)
Major bleeding	327	462
Fatal bleeding	34	55
Intracranial bleeding	52	122
Gastrointestinal bleeding	105	119
Non-major relevant bleeding	286	415
Non ICB GIB major bleeding	275	340
All-cause mortality	603	669

	RE-LY – 150	
	Warfarin (N=6022)	Dabigatran (N=6076)
Major bleeding	421	399
Intracranial bleeding	90	38
Gastrointestinal bleeding	126	188
All-cause mortality	438	487
All-cause mortality denominator	6076	6022

	ROCKET AF	
	Rivaroxaban (N=7111)	Warfarin (N= 7125)
Major bleeding	395	386
Fatal bleeding	27	55
Intracranial bleeding	55	84
Gastrointestinal bleeding	224	154
All cause mortality	208	250
All cause mortality denominator	7061	7082
Non-major relevant bleeding	1185	1151

	ENGAGE AF-TIMI 48 (high dose)	
	Edoxaban (N= 7012)	Warfarin (N= 7012)
Major bleeding	418	524
Fatal bleeding	32	59
Intracranial bleeding	61	132
Gastrointestinal bleeding	232	190
All-cause mortality	773	839
Loss >2g/dl	317	327
Non-major relevant bleeding	286	415
Any overt bleeding	1865	2114
Elevated LFT > 3 fold	5	2

Safety Data Extracted: Venous Thromboembolism

	**RE-COVER**	
	Dabigatran (RCI) (N=1273)	Warfarin (N=1266)
All-cause mortality	21	21
Major bleeding	20	24
Fatal bleeding	1	1
Intracranial bleeding	0	3
Gastrointestinal bleeding	53	35
Loss >2g/dl	20	18
Intramuscular bleeding	8	27
Non-major relevant bleeding	51	87
Any overt bleeding	205	277
Diarrhea	57	38
Edema	43	48
Epistaxis	40	107
Headache	79	88
ALT greater than 3xULN	42	46
Serious adverse event	165	150
Medication stopped (ADE)	126	102
All-cause mortality denominator	1273	1266
ALT greater than 3xULN denominator	1220	1199

	RE-COVER II	
	Dabigatran (RCII) (N=1280)	Warfarin (N=1288)
All-cause mortality	25	25
Major bleeding	15	22
Fatal bleeding	0	1
Intracranial bleeding	2	2
Gastrointestinal bleeding	48	33
Loss >2g/dl	13	19
Intramuscular bleeding	6	20
Serious adverse event	156	153
Medication stopped (ADE)	102	101
All-cause mortality denominator	1280	1288

	EINSTEIN-DVT	
	Rivaroxaban (N=1718)	Enoxaparin, Warfarin/acenocoumarol (N=1711)
All-cause mortality	38	49
Major bleeding	14	20
Fatal bleeding	1	5
ALT greater than 3xULN	25	62
Non-major relevant bleeding	126	119
Serious adverse event	201	233
Medication stopped (ADE)	85	81
All-cause mortality denominator	1718	1711
ALT greater than 3xULN denominator	1680	1649

	AMPLIFY	
	Apixaban	Enoxaparin,Warfarin
Safety Sample	2676	2689
All-cause mortality	41	52
Major bleeding	15	49
Fatal bleeding	1	2
Intracranial bleeding	3	6
Gastrointestinal bleeding	7	18
ALT greater than 3xULN	50	145
Non-major relevant bleeding	103	215
Serious adverse event	417	410
Medication stopped (ADE)	150	182
All -cause mortality denominator	2676	2689
ALT greater than 3xULN denominator	2601	2598

	Hokusai-VTE	
	Edoxaban (N=4118)	LMWH Warfarin (N=4122)
All-cause mortality	132	126
Major bleeding	56	66
Fatal bleeding	2	10
Intracranial bleeding	5	18
Gastrointestinal bleeding	1	2
ALT greater than 3xULN	81	90
Non-major relevant bleeding	298	368
Serious adverse event	503	544
Medication stopped (ADE)	41	51
All-cause mortality denominator	4118	4122
ALT greater than 3xULN denominator	3901	3903
