# Supplementary material for: Efficacy and safety of direct oral anticoagulants approved for cardiovascular indications: Systematic review and meta-analysis
Source: PLoS One. 2018 May 24;13(5):e0197583. doi: 10.1371/journal.pone.0197583 (PMC5967718; doi:10.1371/journal.pone.0197583)
Supplement: S6 File — (PDF) [file pone.0197583.s006.pdf]

Choice of Measure of Effect

Supplement to: Makam RCP, Hoaglin DC, McManus DD, Wang V, Gore JM, Spencer FA, Pradhan R, Tran H, Yu H, Goldberg RJ. Efficacy and safety of direct oral anticoagulants approved for cardiovascular indications: systematic review and meta-analysis.

Patients' adverse drug events generally occur at different times (which are subject to censoring), and the distribution of time to first event may vary among drugs. These features can be captured by an analysis of the time to event (i.e., survival analysis), usually based on a proportional-hazards model (also known as a Cox model) and summarized in the estimated hazard ratio for treatment versus control in an RCT (if the data satisfy the assumption of proportional hazards).

If one has available (or uses) only, for each arm, the number of patients and the number who had an ADE, one can estimate the odds ratio (the ratio of the odds of an event in the treatment arm to the odds of an event in the control arm) or the risk ratio (the corresponding ratio of the proportions of patients who had an event). These measures do not take into account censoring or the time to the first event.

Another type of analysis uses the number of events in each arm and the corresponding total numbers of patient-years of exposure to estimate a rate ratio (the ratio of the number of events per 1000 patient-years, say, in the treatment arm to the corresponding rate in the control arm). Ordinarily this approach counts all events that individual patients experience, not just the first event. Unless one had access to the individual patients' data, this analysis is not feasible.

The meaning of "odds ratio" and "OR" is generally clear. "Risk ratio" and "RR," however, can be ambiguous. Some articles report hazard ratios and label them "risk ratios," and RR could denote a rate ratio. Thus, it is important to determine (if possible) which measure of effect a study used.

The choice of the measure of effect for a meta-analysis depends on the estimates that the various trials report. All four of the trials in our meta-analysis of AF reported estimates of hazard ratios (the report on RE-LY used the label "relative risk") from proportional hazards models, but only four of the five trials in our meta-analysis of VTE reported such estimates (AMPLIFY reported Mantel-Haenszel estimates of relative risks). All nine of the trials reported the numbers of patients and the numbers who had an ADE. Thus, for consistency, our meta-analyses used the odds ratio as the measure of effect. As we describe in another section, our approach worked directly with those counts. Parmar et al. (1998) describe a method for estimating the log-hazard-ratio and its variance from survival curves; but, as they point out, "the published curves are often too small to read accurately, and enlarging them may not help." Also, a report is likely to contain survival curves for only a few ADEs (AMPLIFY reported Kaplan-Meier cumulative event rates only for major bleeding).

Use of the hazard ratio involves two other considerations. First, in meta-analyses the actual measure of effect is the logarithm of the hazard ratio (the estimate produced by a proportional-hazards model). In the standard methods of meta-analysis, the pooled estimate of the log-hazard-ratio is a weighted average of the individual trials' estimates in which the weights are based on the estimated

variances of those estimates. Unfortunately, such estimates are known to be unreliable.

Second, some trials' proportional-hazards models adjust for features of the design. When those adjustment variables differ among trials, the estimated log-hazard-ratios are not comparable.

Reference

Parmar MKB, Torri V, Stewart L (1998). Extracting summary statistics to perform meta-analyses of the published literature for survival endpoints. *Statistics in Medicine* 17:2815-2834.
