# Supplementary material for: Efficacy and safety of direct oral anticoagulants approved for cardiovascular indications: Systematic review and meta-analysis
Source: PLoS One. 2018 May 24;13(5):e0197583. doi: 10.1371/journal.pone.0197583 (PMC5967718; doi:10.1371/journal.pone.0197583)
Supplement: S7 File — (PDF) [file pone.0197583.s007.pdf]

Meta-analysis Methods

Supplement to: Makam RCP, Hoaglin DC, McManus DD, Wang V, Gore JM, Spencer FA, Pradhan R, Tran H, Yu H, Goldberg RJ. Efficacy and safety of direct oral anticoagulants approved for cardiovascular indications: systematic review and meta-analysis.

This section describes the random-effects method used in our meta-analyses, discusses commonly used methods and an alternative method, and comments on the use of the statistics Q and I^2 to assess heterogeneity of the effects from individual trials.

Meta-analysis of Odds Ratios Based on Binomial Likelihood

For a particular ADE the basic data are the number of patients and the number who experienced the ADE in each arm of the trial. For trial i ($i=1,\ldots,k$) the numbers of patients in the treated and control arms are n_{iT} and n_{iC}, respectively, and the corresponding numbers of patients who experienced the ADE are x_{iT} and x_{iC}. As is customary, we assume that x_{iT} and x_{iC} are observations from binomial distributions in which the probabilities of experiencing the ADE are p_{iT} and p_{iC}:

$$x_{iT} \sim \text{binomial}(n_{iT}, p_{iT})$$

$$x_{iC} \sim \text{binomial}(n_{iC}, p_{iC}).$$

If p is the probability of an event, then the odds of an event are $p/(1-p)$, and the log-odds are $\log_e[p/(1-p)]$. (This logarithmic transformation is known as the logit: $\text{logit}(p) = \log_e[p/(1-p)]$.) For the treatment versus the control the odds ratio is

$$\frac{p_T/(1-p_T)}{p_C/(1-p_C)}.$$

Analyses of odds ratios customarily use the logarithmic scale. Our random-effects meta-analysis used the following model:

$$\text{logit}(p_{iC}) = g_i$$
$$\text{logit}(p_{iT}) = g_i + \mu + u_i$$
$$u_i \sim \text{Normal}(0, \tau^2);$$

g_i is the log-odds for experiencing the ADE in the control arm of trial i, and $g_i + \mu + u_i$ is the log-odds for experiencing the ADE in the treatment arm. Thus, $\mu + u_i$ is the log of the odds ratio, in trial i, for experiencing the ADE in the treatment arm, relative to the control arm. That is, μ is the overall average log-odds-ratio, which the meta-analysis seeks to estimate, and the random effect u_i is the departure

of the log-odds-ratio in trial *i* from the overall average. The u_i attempt to account for heterogeneity of the log-odds-ratios among the *k* trials, summarized by τ^2, the variance of the distribution of random effects. In this mixed-effects logistic regression model, introduced as a multilevel model by Turner et al. (2000), the data n_{iT}, x_{iT}, n_{iC}, and x_{iC} enter through the binomial likelihood. Chang and Hoaglin (2017) compare this model with other approaches in the context of an example.

The effect of treatment, relative to control, is random, but each trial has its own log-odds for the control arm, g_i. The g_i are "nuisance parameters"; they are fixed effects, rather than random effects. Some models put a second hierarchical model on the g_i, and others put a bivariate normal model on the g_i and the u_i; but Dias et al. (2013) point out that, unless that model is correct, the estimated relative treatment effects will be biased.

Commonly Used Methods of Meta-analysis

For meta-analysis of odds ratios, the common methods use either a fixed-effect method or a random-effects method. Fixed-effect methods assume that a single overall odds ratio underlies all of the trials and that the observed trial-level odds ratios differ from that true odds ratio only because of sampling variation within each trial. This assumption may be appropriate when the studies have essentially the same design, treatments, patient population, and outcome measures. Usually, however, designs, patients' characteristics, and details of the treatments vary among studies; and it is more realistic to account for variation in the true odds ratios by regarding them as coming from a distribution of study-level odds ratios. Thus, random-effects methods focus on estimating the mean of that distribution and also the distribution's variance (as a summary of the heterogeneity of the study-level effects).

The common fixed-effect and random-effects methods use the logarithm of each trial's sample odds ratio, along with an estimate of its variance. In the notation of the preceding section, the logarithm of the sample odds ratio in trial *i* is

$$y_i = \log(x_{iT}) - \log(n_{iT} - x_{iT}) - \log(x_{iC}) + \log(n_{iC} - x_{iC}),$$

and the usual estimate of its variance is

$$s_i^2 = \frac{1}{x_{iT}} + \frac{1}{n_{iT} - x_{iT}} + \frac{1}{x_{iC}} + \frac{1}{n_{iC} - x_{iC}} .$$

Two limitations are seldom mentioned. First, the sample log-odds, for example,

$$\log(x_{iT}) - \log(n_{iT} - x_{iT})$$

is a biased estimate of the population log-odds

$$\log(p_{iT}) - \log(1 - p_{iT}),$$

especially in small samples. Modifications are available that reduce the bias, but they are not routinely used. Second, technically, the formula for s_i^2 cannot be correct: whenever any of x_{iT}, $n_{iT} - x_{iT}$, x_{iC}, or $n_{iC} - x_{iC}$ has a positive probability of being 0, the true variance of log(OR) is not finite. The formula corresponds to the variance of the normal distribution that the distribution of log(OR) approaches as n_{iT} and n_{iC} become large. Adding a positive constant to each of the four counts avoids non-finite variance, but hardly any empirical evidence is available on how closely the distribution of the modified log(OR) resembles a normal distribution.

The conventional fixed-effect estimate of the overall log-odds-ratio is a weighted mean with inverse-variance weights, $w_i = 1/s_i^2$:

$$\bar{y}_w = \frac{\Sigma w_i y_i}{\Sigma w_i}.$$

It is customary to estimate the variance of $\bar{y}_w$ by $1/\Sigma w_i$, but this approach relies heavily on the assumption that $s_i^2 = \sigma_i^2$, the true within-trial variance of y_i or on the assumption that the number of patients in each arm is large.

In the conventional random-effects model the observed trial-level effects have two sources of variation: within study (σ_i^2) and between studies (τ^2). That is, $\mathrm{var}(y_i) = \sigma_i^2 + \tau^2$. The conventional random-effects estimate of the overall log-odds-ratio is another weighted mean, now with weights that reflect both sources of variation, $w_i^* = 1/(s_i^2 + \hat{\tau}^2)$:

$$\bar{y}_w^* = \frac{\Sigma w_i^* y_i}{\Sigma w_i^*}.$$

The estimate of τ^2, denoted by $\hat{\tau}^2$, is derived from the y_i and the s_i^2. The customary estimate of the variance of $\bar{y}_w^*$ is $1/\Sigma w_i^*$. As in the fixed-effect method, it substitutes s_i^2 for the unknown σ_i^2. Another potential source of complications is its use of $\hat{\tau}^2$ as if it were the true value, τ^2 (i.e., without making allowance for the variability of $\hat{\tau}^2$).

The random-effects approach summarized above is the basis for the procedure described by DerSimonian and Laird (1986). They obtain an estimate of τ^2 by using the method of moments: setting $Q = \Sigma w_i (y_i - \bar{y}_w)^2$ equal to an expression for its expected value and solving to produce

$$\hat{\tau}^2 = \max\{0, [Q - (k-1)] / [\Sigma w_i - (\Sigma w_i^2 / \Sigma w_i)]\}$$

(the max operation avoids negative estimates of between-study variance, but $\hat{\tau}^2 = 0$ is still possible; then $w_i^* = w_i$ and $\bar{y}_w^* = \bar{y}_w$). Unfortunately, the derivation of the expected value of *Q* assumes that $s_i^2 = \sigma_i^2$. A number of studies, using extensive simulations, have found that the resulting $1/\Sigma w_i^*$ tends to underestimate the variance of $\bar{y}_w^*$ and leads to confidence intervals (based on a normal distribution) that have lower than nominal coverage (e.g., at the 95% level) of the overall effect. Also, some studies have found substantial bias in $\bar{y}_w^*$. Despite these shortcomings, the DerSimonian-Laird procedure (DL) is the default method in many meta-analysis software programs.

For the log-odds-ratio in particular, bias is a potential problem for both $\bar{y}_w$ and $\bar{y}_w^*$ because the y_i and the s_i^2 are correlated: both are functions of x_{iT}, n_{iT}, x_{iC}, and n_{iC}.

Through its use of the data in a binomial likelihood, the mixed-effects logistic regression model avoids the assumptions and approximations involved in the common methods.

Meta-analysis of Rate Ratios Based on a Poisson Model

An alternative approach compares treatments on their rates of an ADE per 1000 person-years. Cooper et al. (2006) illustrate this approach in a Bayesian network meta-analysis, using a random-effects Poisson regression model. The data for treatment *j* in trial *i* are the number of events, x_{ij}, and the number of patient-years of follow-up, t_{ij}. The model considers x_{ij} to be an observation from the Poisson

distribution with mean λ_{ij}, and it relates $\log(\lambda_{ij})$ to $\log(t_{ij}/1000)$ and parameters for the log of the rate of an event on the base treatment in trial *i* and the trial-specific log-rate-ratio of treatment *j* relative to the base treatment.

As Cooper et al. point out, the analysis of rates has the advantage of incorporating the duration of the trials, and it also handles data that count multiple events per patient. One can use this approach, however, only when the various trials report the necessary data. For example, Granger et al. (2011) report the number of patients with an event and the event rate (%/yr), from which one could estimate the number of patient-years of follow-up (assuming that a patient's contribution ends when the patient has an event or is lost to follow-up or at the cutoff date for the analysis or at end of the trial). Some trials, however, do not report the number of patient-years of follow-up (or equivalent information). Interestingly, in a later paper Cooper et al. (2009) use the odds ratio as the measure of effect.

Heterogeneity

Most published meta-analyses assess the heterogeneity of the trial-level estimates y_i by using two statistics, Cochran's *Q* and the related measure denoted by I^2. As defined earlier,

$$Q = \sum_{i=1}^{k} w_i (y_i - \bar{y}_w)^2 .$$

The customary test for heterogeneity refers *Q* to the chi-squared distribution on $k-1$ degrees of freedom, making the assumption that *Q* follows that distribution under the null hypothesis of no heterogeneity. Actually, however, the null distribution of *Q* only approaches that chi-squared distribution as the sample sizes of the trials become large (and criteria for "large" are not well established). If those sample sizes are not large, the null distribution of *Q* is not well approximated by the chi-squared distribution on $k-1$ degrees of freedom. Indeed, the null distribution of *Q* differs substantially among measures of effect (so far, results are available for only two measures besides log-odds-ratio). Hoaglin (2016) reviews results on the behavior of *Q*. An important complication is that the null distributions involve parameters that must be estimated from the data in each meta-analysis.

The difficulties of *Q* carry over to the heterogeneity measure I^2 (Higgins and Thompson 2002), which is usually calculated from

$$I^2 = 100 \times \max\left\{0, \frac{Q-(k-1)}{Q}\right\}$$

and interpreted as the percentage of total variation in the estimates of treatment effect that is due to heterogeneity among studies. That appealing interpretation, however, overlooks the fact that, even if the null distribution of Q were chi-squared on $k-1$ degrees of freedom, the probability of values exceeding the mean ($k-1$) would not be small. For example, when $k = 4$, that probability is .392; and it increases to .465 when $k=30$, to .481 when $k=100$, and to .5 in the limit (from the normal approximation). That is, values of I^2 that would be interpreted as evidence of heterogeneity are quite likely under the assumed chi-squared null distribution, which corresponds to absence of heterogeneity (Hoaglin 2016).

Instead of using the mean as the threshold for positive values of I^2, it might be possible to choose a suitable quantile, substantially above the median; but adapting I^2 to the correct null distribution of Q would still require a different expression for each measure of effect, and the values of those expressions would have to be calculated anew for each meta-analysis.

References

Chang BH, Hoaglin DC (2017). Meta-analysis of odds ratios: current good practices. *Medical Care* 55:328-335.

Cooper NJ, Sutton AJ, Lu G, Khunti K (2006). Mixed comparison of stroke prevention treatments in individuals with nonrheumatic atrial fibrillation. *Archives of Internal Medicine* 166:1269-1275.

Cooper NJ, Sutton AJ, Morris D, Ades AE, Welton NJ (2009). Addressing between-study heterogeneity and inconsistency in mixed treatment comparisons: applications to stroke prevention treatments in individuals with non-rheumatic atrial fibrillation. *Statistics in Medicine* 28:1861-1881.

DerSimonian R, Laird N (1986). Meta-analysis in clinical trials. *Controlled Clinical Trials* 7:177-188.

Dias S, Sutton AJ, Ades AE, Welton NJ (2013). Evidence synthesis for decision making 2: a generalized linear modeling framework for pairwise and network meta-analysis of randomized controlled trials. *Medical Decision Making* 33:607-617.

Granger CB, Alexander JH, McMurray JJV, et al. (2011). Apixaban versus warfarin in patients with atrial fibrillation. *New England Journal of Medicine* 365:981-992.

Higgins JPT, Thompson SG (2002). Quantifying heterogeneity in a meta-analysis. *Statistics in Medicine* 21:1539-1558.

Hoaglin DC (2016). Misunderstandings about Q and "Cochran's Q test" in meta-analysis. *Statistics in Medicine* 35:485-504. With discussion and a reply by the author.

Turner RM, Omar RZ, Yang M, Goldstein H, Thompson SG (2000). A multilevel model framework for meta-analysis of clinical trials with binary outcomes. *Statistics in Medicine* 19:3417-3432.
