# Supplementary material for: Efficacy and safety of direct oral anticoagulants approved for cardiovascular indications: Systematic review and meta-analysis
Source: PLoS One. 2018 May 24;13(5):e0197583. doi: 10.1371/journal.pone.0197583 (PMC5967718; doi:10.1371/journal.pone.0197583)
Supplement: S8 File — (PDF) [file pone.0197583.s008.pdf]

Details of Models Used for Meta-analysis

Supplement to: Makam RCP, Hoaglin DC, McManus DD, Wang V, Gore JM, Spencer FA, Pradhan R, Tran H, Yu H, Goldberg RJ. Efficacy and safety of direct oral anticoagulants approved for cardiovascular indications: systematic review and meta-analysis.

R code used for random-effects meta-analysis of odd ratios

```
library(lme4)

# Variable definitions
# id is study_id  (1 to 4 for safety data, 1 to 5 for efficacy data)
# n is sample_size
# arm is study_group  (0: control, 1: intervention)
# ADE is the number_of_occurrences_of the ADE (PO in the example below)

# nAGQ=7 specifies that the estimation method is adaptive Gauss-Hermite
# quadrature (AGHQ) based on 7 quadrature points.

# AF_ Efficacy is the Data Used table in Results of Meta-analyses: Non-valvular
# atrial fibrillation: Efficacy

id <- rep(1:4, times=2)
arm <- c(rep(1,4), rep(0, 4))
n <- AF_ Efficacy$`Sample Size`

PO <- AF_ Efficacy $`Stroke or Systemic Embolism (PO)`
nPO <- n - PO

results <- glmer(cbind(PO, nPO)~ as.factor(arm) + as.factor(id) + (-1+arm|id),
family=binomial, nAGQ=7)

# model-fitting summary.  See "Stroke or systemic embolism" in Efficacy section of
# Results of Meta-analyses: Non-valvular atrial fibrillation

summary <- summary(results)

# Odds ratio and 95% confidence interval

Lor <- cbind(est= fixef(results), confint(results,parm="beta_",method="Wald",
level= 0.95))
OR <- exp(Lor[2,1])
UpperCI <- exp(Lor[2,2])
LowerCI <- exp(Lor[2,3])
```
