# Supplementary material for: Efficacy and safety of direct oral anticoagulants approved for cardiovascular indications: Systematic review and meta-analysis
Source: PLoS One. 2018 May 24;13(5):e0197583. doi: 10.1371/journal.pone.0197583 (PMC5967718; doi:10.1371/journal.pone.0197583)

Results of Meta-analyses

Supplement to: Makam RCP, Hoaglin DC, McManus DD, Wang V, Gore JM, Spencer FA, Pradhan R, Tran H, Yu H, Goldberg RJ. Efficacy and safety of direct oral anticoagulants approved for cardiovascular indications: systematic review and meta-analysis.

Results of Meta-analyses: Non-valvular atrial fibrillation

Efficacy

Data Used

Trial	Drug	Sample Size	Stroke or Systemic Embolism (PO)	Systemic Embolism	Any Stroke	Hemorrhagic Stroke	Death from Any Cause	Death from Vascular Causes	Myocardial Infarction
**ARISTOTLE**	Apixaban	9120	212	15	199	40	603		90
**ENGAGE-AF**	Edoxaban	7035	182	15	281	49	773	530	133
**RE-LY – 150**	Dabigatran	6076	134		122	12	438	274	97
**ROCKET I**	Rivaroxaban	6958	188	5	184	29	208	170	101
**ARISTOTLE**	Warfarin	9081	265	17	250	78	669		102
**ENGAGE-AF**	Warfarin	7036	232	23	317	90	839	611	141
**RE-LY – 150**	Warfarin	6022	202		186	45	487	317	75
**ROCKET I**	Warfarin	7004	241	22	221	50	250	193	126

Stroke or systemic embolism

```
Generalized linear mixed model fit by maximum likelihood (Adaptive Gauss-Hermite
Quadrature, nAGQ = 7) ['glmerMod']
 Family: binomial  ( logit )
Formula: cbind(ADEs[, i], nADEs[, i]) ~ as.factor(arm) + as.factor(id) +      (-1 + arm | id)

   AIC      BIC   logLik deviance df.resid
  14.2     14.7     -1.1      2.2        2

Scaled residuals:
    Min       1Q   Median       3Q      Max
-0.99625 -0.24489  0.01467  0.28001  0.87612

Random effects:
 Groups Name Variance Std.Dev.
 id     arm  0        0
Number of obs: 8, groups:  id, 4

Fixed effects:
               Estimate Std. Error z value Pr(>|z|)
(Intercept)     -3.48348    0.05131  -67.90  < 2e-16 ***
as.factor(arm)1 -0.28148    0.05031   -5.59 2.21e-08 ***
as.factor(id)2   0.11877    0.06815    1.74   0.0814 .
as.factor(id)3   0.06000    0.07223    0.83   0.4062
as.factor(id)4   0.16306    0.06753    2.41   0.0158 *
---
Signif. codes:  0 ‘***’ 0.001 ‘**’ 0.01 ‘*’ 0.05 ‘.’ 0.1 ‘ ‘ 1

Correlation of Fixed Effects:
            (Intr) as.()1 as.()2 as.()3
as.fctr(r)1 -0.426
as.fctr(d)2 -0.616  0.000
as.fctr(d)3 -0.581 -0.001  0.438
as.fctr(d)4 -0.622  0.002  0.468  0.442
```

Odds Ratio of Studies for Stroke or Systemic Embolism (PO)

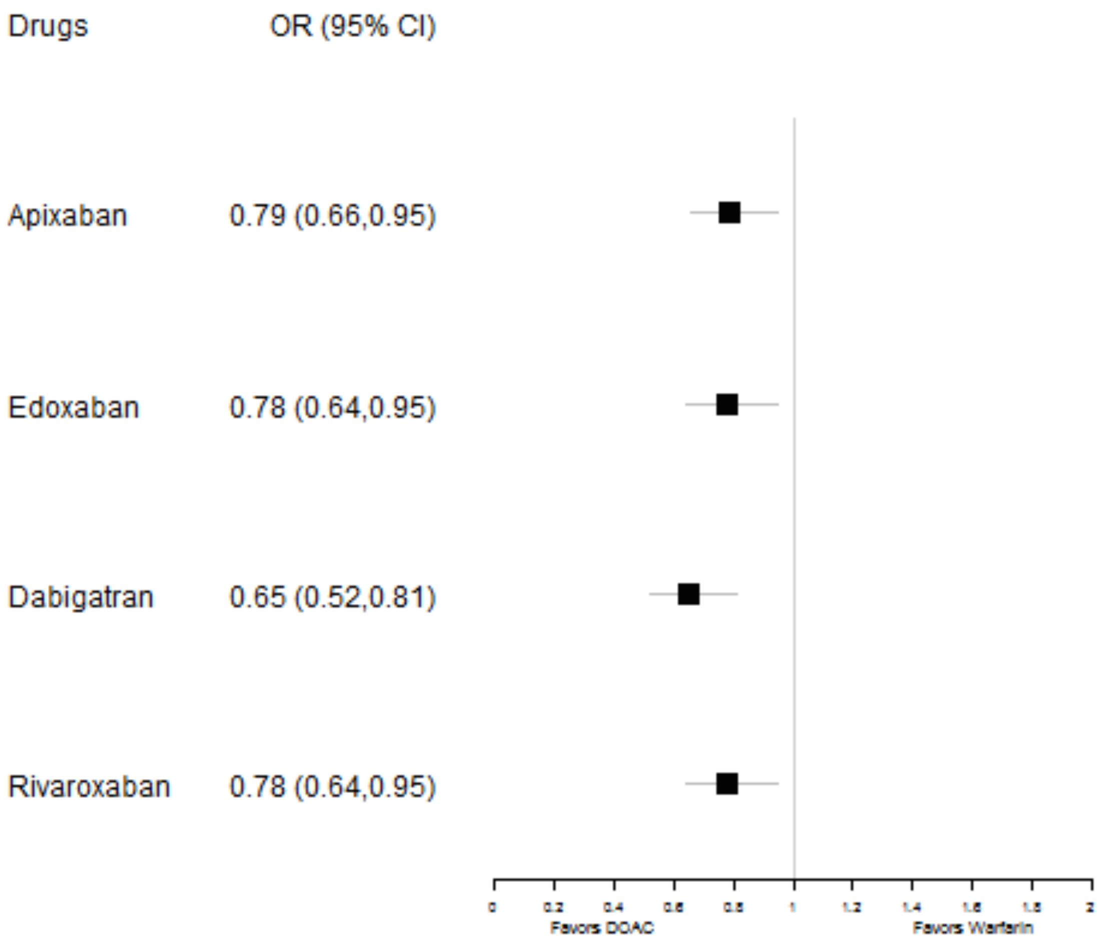

Systemic embolism

```
Generalized linear mixed model fit by maximum likelihood (Adaptive Gauss-Hermite
Quadrature, nAGQ = 7) ['glmerMod']
 Family: binomial  ( logit )
Formula: cbind(ADEs[, i], nADEs[, i]) ~ as.factor(arm) + as.factor(id) +      (-1 + arm | id)

    AIC      BIC   logLik deviance df.resid
   15.7     14.6     -2.8      5.7        1

Scaled residuals:
    Min       1Q   Median       3Q      Max
-1.43128 -0.59779  0.04713  0.79839  1.05525

Random effects:
 Groups Name Variance Std.Dev.
 id     arm  0.009755 0.09877
Number of obs: 6, groups:  id, 3

Fixed effects:
                Estimate Std. Error z value Pr(>|z|)
(Intercept)      -6.1055     0.2783 -21.937   <2e-16 ***
as.factor(arm)1  -0.5800     0.2611  -2.222   0.0263 *
as.factor(id)2    0.4368     0.2821   1.549   0.1215
as.factor(id)4    0.1206     0.5722   0.211   0.8331
---
Signif. codes:  0 '***' 0.001 '**' 0.01 '*' 0.05 '.' 0.1 ' ' 1

Correlation of Fixed Effects:
            (Intr) as.()1 as.()2
as.fctr(r)1  0.158
as.fctr(d)2 -0.768 -0.262
as.fctr(d)4 -0.836 -0.475  0.639
```

Odds Ratio of Studies for Systemic Embolism

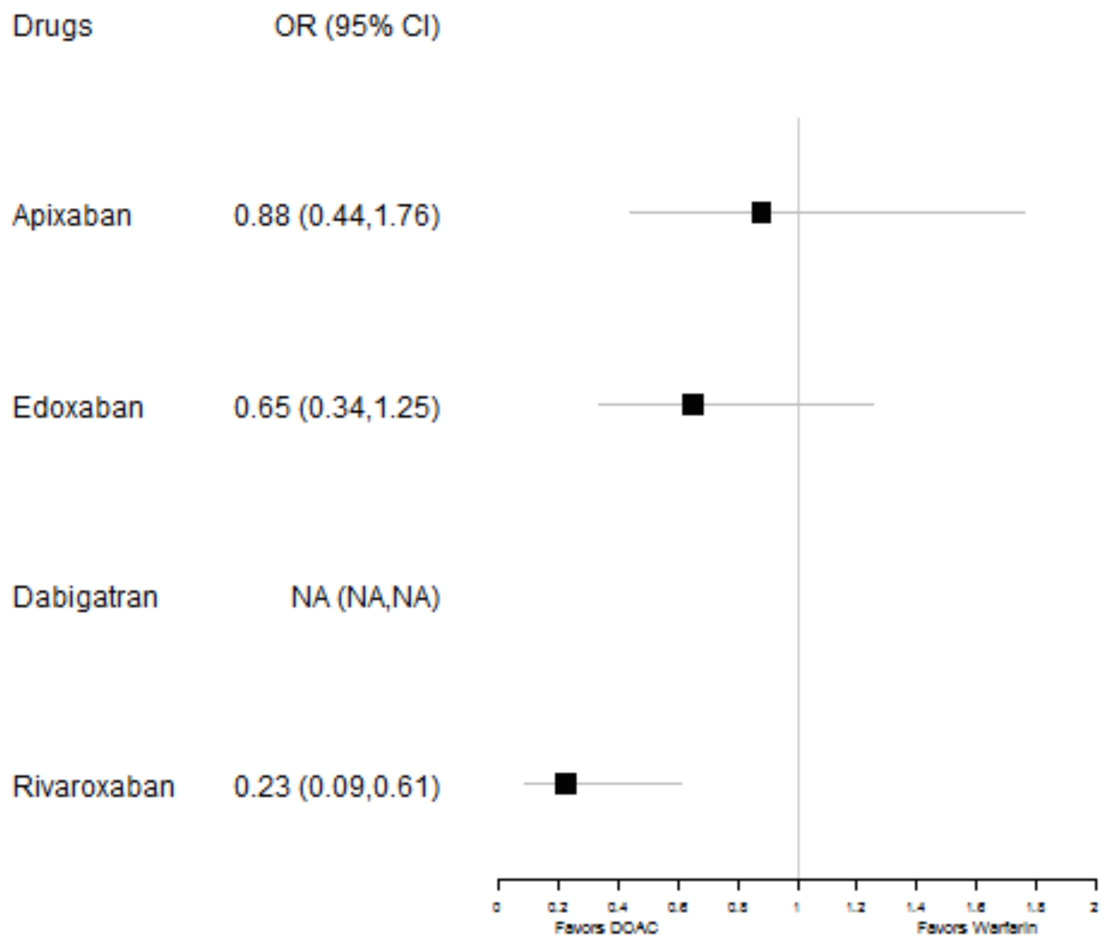

Any stroke

```
Generalized linear mixed model fit by maximum likelihood (Adaptive Gauss-Hermite
Quadrature, nAGQ = 7) ['glmerMod']
 Family: binomial  ( logit )
Formula: cbind(ADEs[, i], nADEs[, i]) ~ as.factor(arm) + as.factor(id) +      (-1 + arm | id)

    AIC      BIC   logLik deviance df.resid
   17.0     17.5     -2.5      5.0        2

Scaled residuals:
    Min       1Q   Median       3Q      Max
-1.38088 -0.39535 -0.00596  0.43917  1.24798

Random effects:
 Groups Name Variance Std.Dev.
 id     arm  0        0
Number of obs: 8, groups:  id, 4

Fixed effects:
                 Estimate Std. Error z value Pr(>|z|)
(Intercept)      -3.57156    0.05255  -67.97  < 2e-16 ***
as.factor(arm)1  -0.22261    0.04872   -4.57  4.9e-06 ***
as.factor(id)2    0.56235    0.06349    8.86  < 2e-16 ***
as.factor(id)3    0.03259    0.07495    0.43    0.664
as.factor(id)4    0.16591    0.06948    2.39    0.017 *
---
Signif. codes:  0 ‘***’ 0.001 ‘**’ 0.01 ‘*’ 0.05 ‘.’ 0.1 ‘ ’ 1

Correlation of Fixed Effects:
            (Intr) as.()1 as.()2 as.()3
as.fctr(r)1 -0.416
as.fctr(d)2 -0.684 -0.001
as.fctr(d)3 -0.580 -0.001  0.480
as.fctr(d)4 -0.626  0.002  0.518  0.439
```

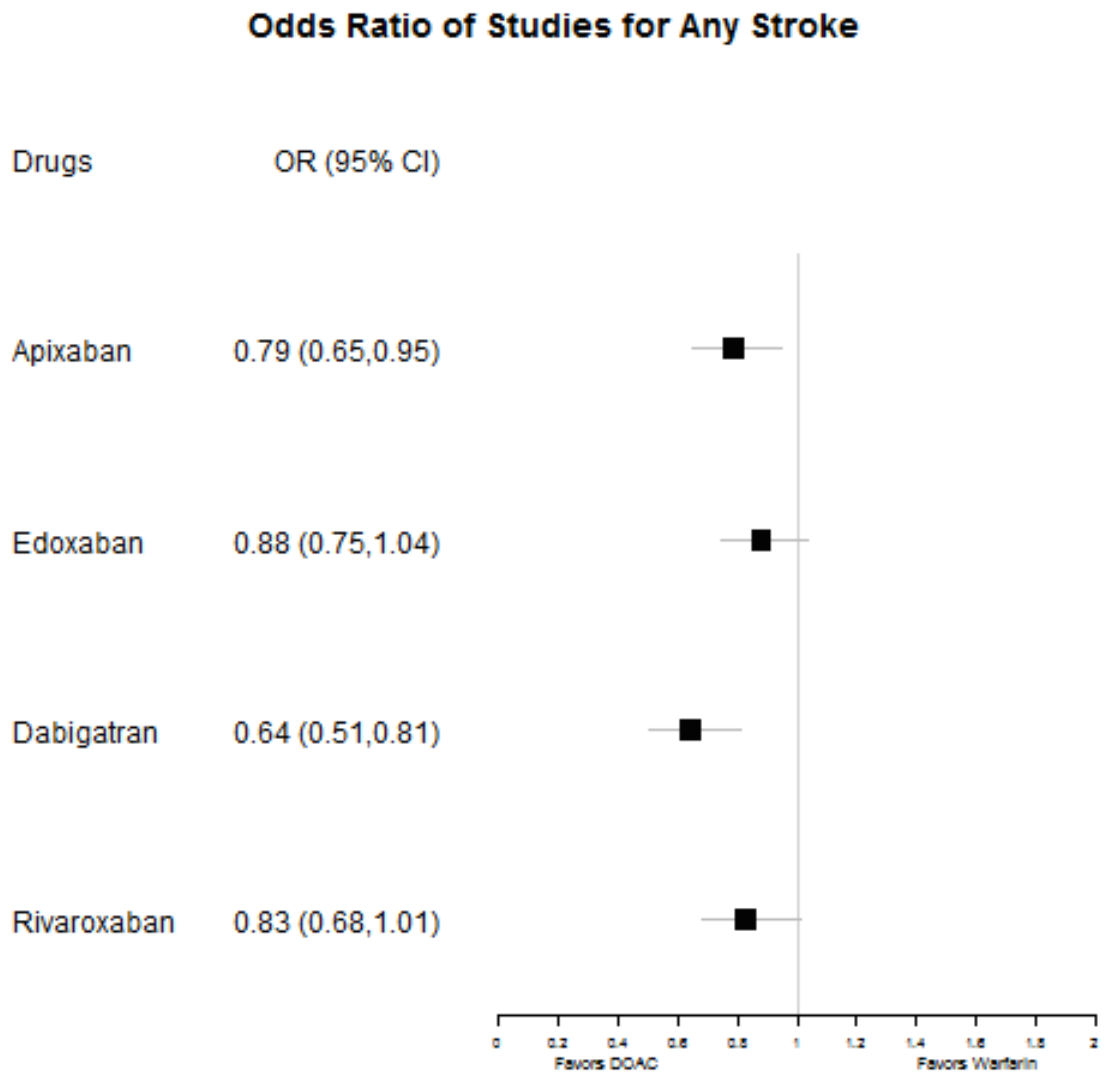
Odds Ratio of Studies for Any Stroke
Drugs
OR (95% CI)
Apixaban
0.79 (0.65,0.95)
Edoxaban
0.88 (0.75,1.04)
Dabigatran
0.64 (0.51,0.81)
Rivaroxaban
0.83 (0.68,1.01)
Favors DOAC
Favors Warfarin

Hemorrhagic stroke

```
Generalized linear mixed model fit by maximum likelihood (Adaptive Gauss-Hermite
Quadrature, nAGQ = 7) ['glmerMod']
 Family: binomial  ( logit )
Formula: cbind(ADEs[, i], nADEs[, i]) ~ as.factor(arm) + as.factor(id) +      (-1 + arm | id)

    AIC      BIC   logLik deviance df.resid
   17.0     17.4     -2.5      5.0        2

Scaled residuals:
    Min       1Q   Median       3Q      Max
-1.59536 -0.33959  0.02125  0.48220  1.12681

Random effects:
 Groups Name Variance Std.Dev.
 id     arm  0        0
Number of obs: 8, groups:  id, 4

Fixed effects:
               Estimate Std. Error z value Pr(>|z|)
(Intercept)     -4.73703    0.09907  -47.82  < 2e-16 ***
as.factor(arm)1 -0.71079    0.10756   -6.61 3.89e-11 ***
as.factor(id)2   0.42421    0.12574    3.37 0.000742 ***
as.factor(id)3  -0.32039    0.16178   -1.98 0.047657 *
as.factor(id)4  -0.13888    0.14586   -0.95 0.341011
---
Signif. codes:  0 ‘***’ 0.001 ‘**’ 0.01 ‘*’ 0.05 ‘.’ 0.1 ‘ ’ 1

Correlation of Fixed Effects:
            (Intr) as.()1 as.()2 as.()3
as.fctr(r)1 -0.361
as.fctr(d)2 -0.685  0.000
as.fctr(d)3 -0.533  0.000  0.420
as.fctr(d)4 -0.591  0.002  0.465  0.362
```

Odds Ratio of Studies for Hemorrhagic Stroke

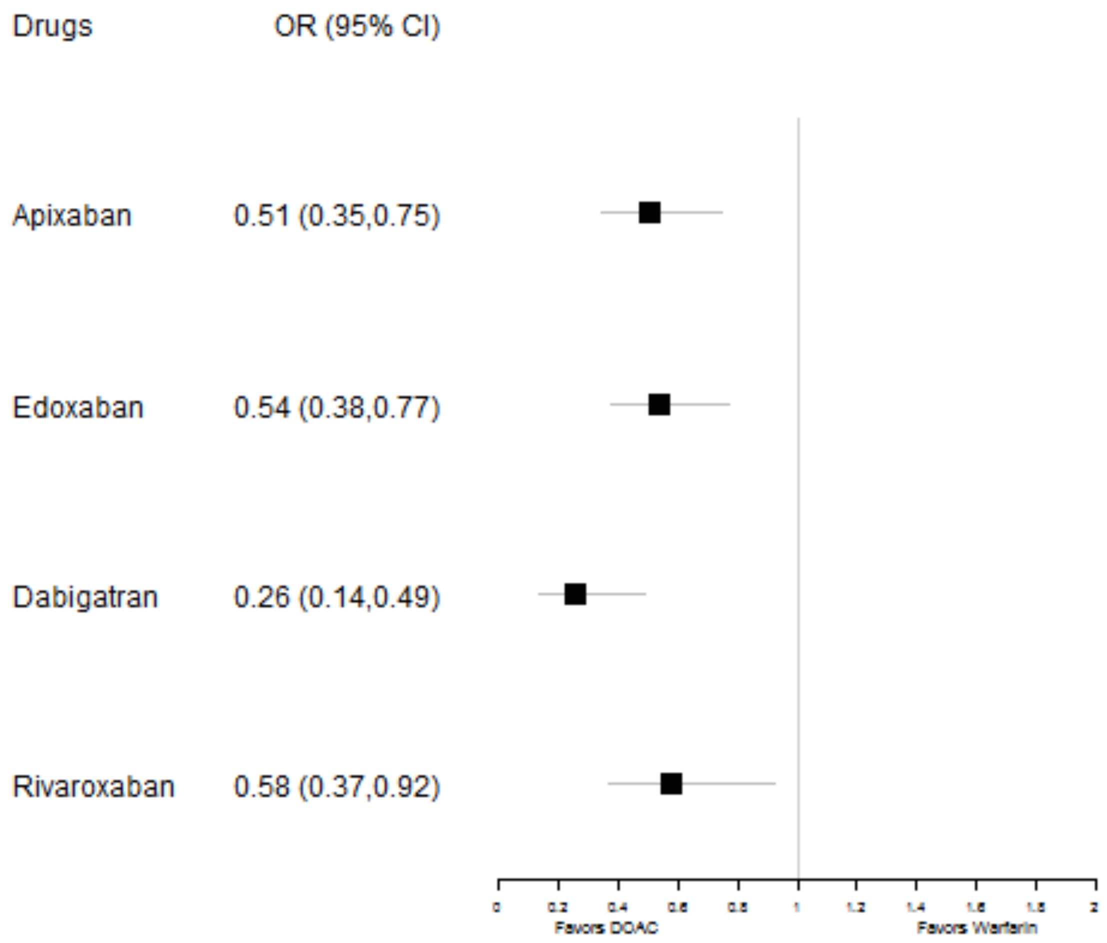

Death from any cause

```
Generalized linear mixed model fit by maximum likelihood (Adaptive Gauss-Hermite
Quadrature, nAGQ = 7) ['glmerMod']
 Family: binomial  ( logit )
Formula: cbind(ADEs[, i], nADEs[, i]) ~ as.factor(arm) + as.factor(id) +      (-1 + arm | id)

    AIC      BIC   logLik deviance df.resid
   12.7     13.2     -0.4      0.7        2

Scaled residuals:
    Min       1Q   Median       3Q      Max
-0.50637 -0.13996  0.00019  0.14102  0.47788

Random effects:
 Groups Name Variance Std.Dev.
 id     arm  0        0
Number of obs: 8, groups:  id, 4

Fixed effects:
              Estimate Std. Error z value Pr(>|z|)
(Intercept)     -2.53133    0.03283  -77.10  < 2e-16 ***
as.factor(arm)1 -0.11691    0.03203   -3.65 0.000263 ***
as.factor(id)2   0.54349    0.03932   13.82  < 2e-16 ***
as.factor(id)3   0.09713    0.04490    2.16 0.030526 *
as.factor(id)4  -0.79588    0.05571  -14.29  < 2e-16 ***
---
Signif. codes:  0 '***' 0.001 '**' 0.01 '*' 0.05 '.' 0.1 ' ' 1

Correlation of Fixed Effects:
            (Intr) as.()1 as.()2 as.()3
as.fctr(r)1 -0.464
as.fctr(d)2 -0.654 -0.001
as.fctr(d)3 -0.573 -0.001  0.479
as.fctr(d)4 -0.464  0.003  0.386  0.338
```

Odds Ratio of Studies for Death from Any Cause

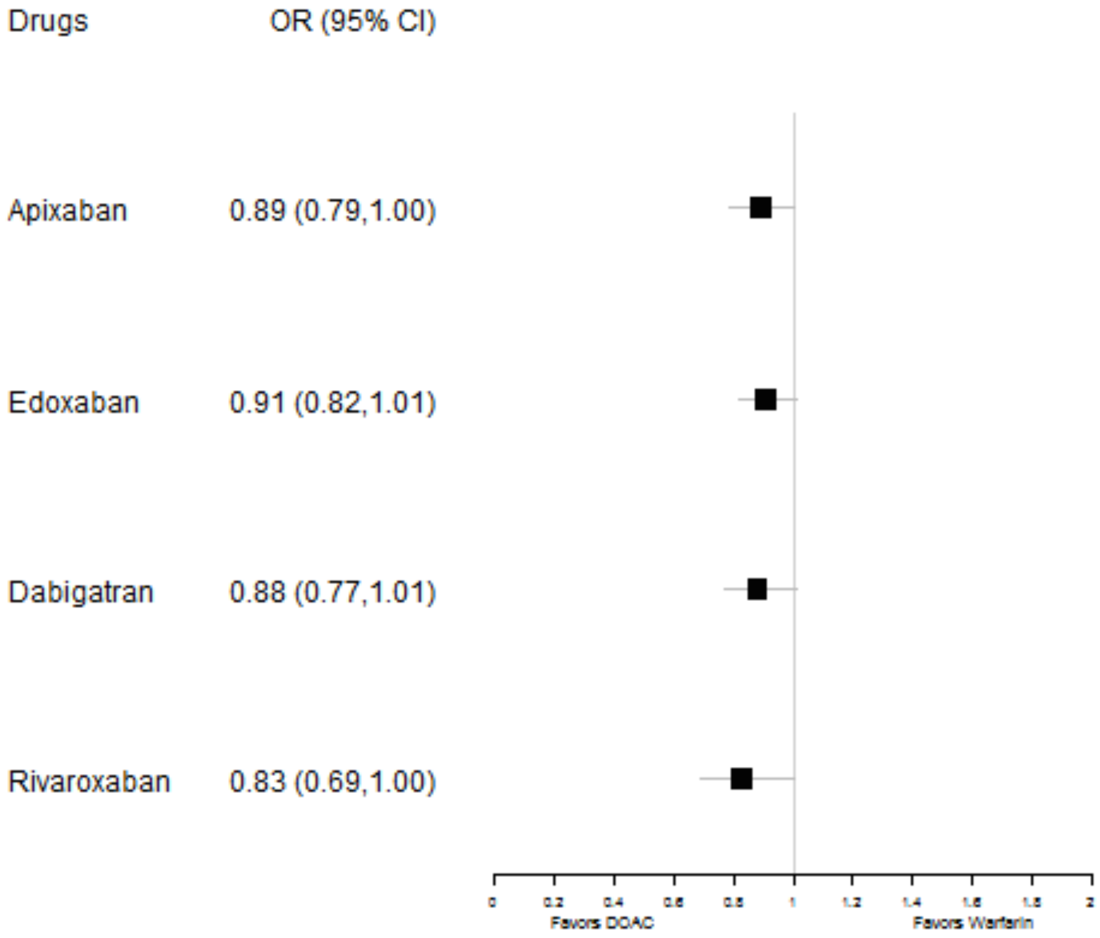

Death from vascular causes

```
Generalized linear mixed model fit by maximum likelihood (Adaptive Gauss-Hermite
Quadrature, nAGQ = 7) ['glmerMod']
 Family: binomial  ( logit )
Formula: cbind(ADEs[, i], nADEs[, i]) ~ as.factor(arm) + as.factor(id) +      (-1 + arm | id)

    AIC      BIC   logLik deviance df.resid
   10.1      9.0      0.0      0.1        1

Scaled residuals:
      Min        1Q    Median        3Q       Max
-0.177361 -0.082998 -0.001193  0.077870  0.191178

Random effects:
 Groups Name Variance Std.Dev.
 id     arm  0        0
Number of obs: 6, groups:  id, 3

Fixed effects:
                 Estimate Std. Error z value Pr(>|z|)
(Intercept)      -2.35440    0.03746  -62.86  < 2e-16 ***
as.factor(arm)1 -0.15130    0.04523   -3.34 0.000823 ***
as.factor(id)3  -0.54109    0.05228  -10.35  < 2e-16 ***
as.factor(id)4  -1.19626    0.06151  -19.45  < 2e-16 ***
---
Signif. codes:  0 '***' 0.001 '**' 0.01 '*' 0.05 '.' 0.1 ' ' 1

Correlation of Fixed Effects:
            (Intr) as.()1 as.()3
as.fctr(r)1 -0.566
as.fctr(d)3 -0.487  0.000
as.fctr(d)4 -0.417  0.004  0.297
```

Odds Ratio of Studies for Death from Vascular Causes

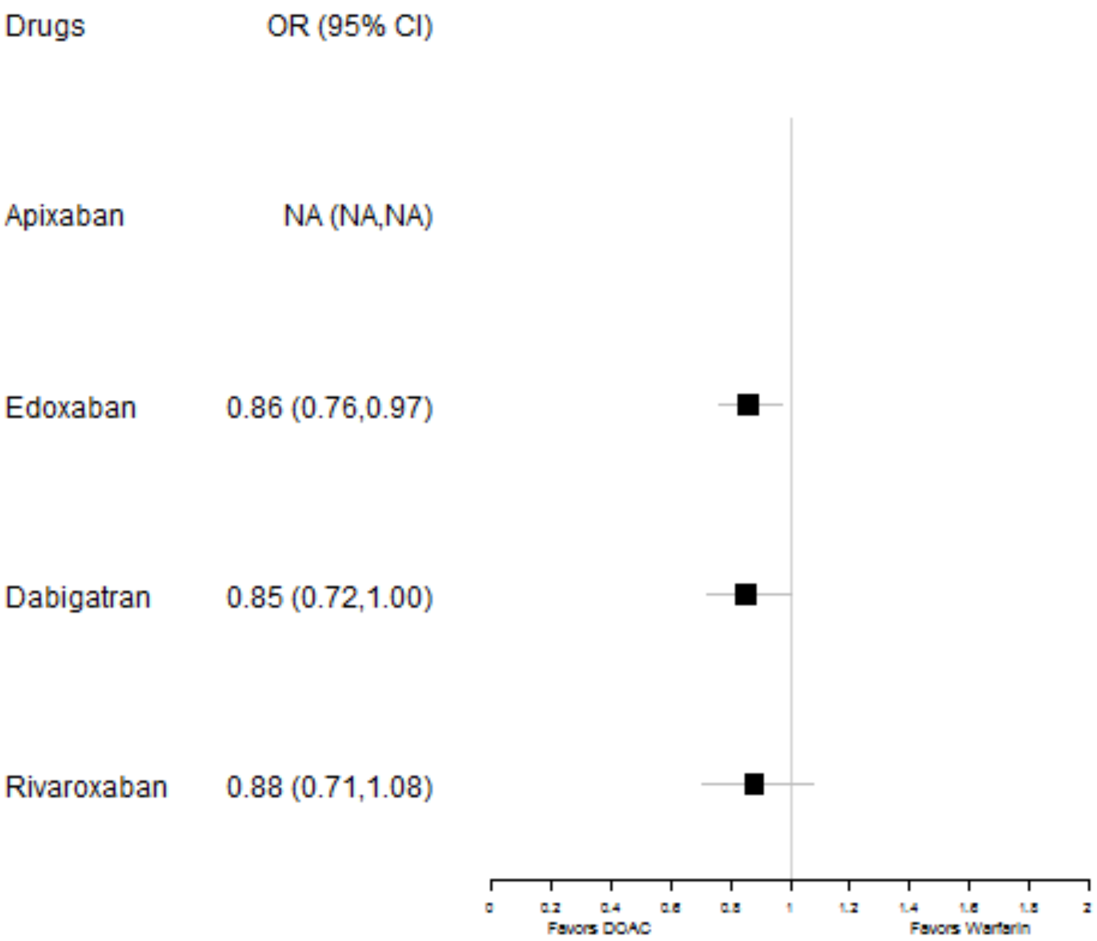

Myocardial infarction

```
Generalized linear mixed model fit by maximum likelihood (Adaptive Gauss-Hermite
Quadrature, nAGQ = 7) ['glmerMod']
 Family: binomial  ( logit )
Formula: cbind(ADEs[, i], nADEs[, i]) ~ as.factor(arm) + as.factor(id) +      (-1 + arm | id)

    AIC      BIC   logLik deviance df.resid
   17.7     18.2     -2.9      5.7        2

Scaled residuals:
    Min       1Q   Median       3Q      Max
-1.39079 -0.49761 -0.00034  0.48434  1.42209

Random effects:
 Groups Name Variance Std.Dev.
 id     arm  0        0
Number of obs: 8, groups:  id, 4

Fixed effects:
                Estimate Std. Error z value Pr(>|z|)
(Intercept)     -4.51394    0.07989  -56.51  < 2e-16 ***
as.factor(arm)1 -0.05500    0.06856   -0.80  0.42243
as.factor(id)2   0.62200    0.09479    6.56 5.33e-11 ***
as.factor(id)3   0.30222    0.10565    2.86  0.00423 **
as.factor(id)4   0.43823    0.09870    4.44 9.00e-06 ***
---
Signif. codes:  0 ‘***’ 0.001 ‘**’ 0.01 ‘*’ 0.05 ‘.’ 0.1 ‘ ’ 1

Correlation of Fixed Effects:
           (Intr) as.()1 as.()2 as.()3
as.fctr(r)1 -0.419
as.fctr(d)2 -0.695  0.001
as.fctr(d)3 -0.623 -0.001  0.526
as.fctr(d)4 -0.668  0.002  0.563  0.505
```

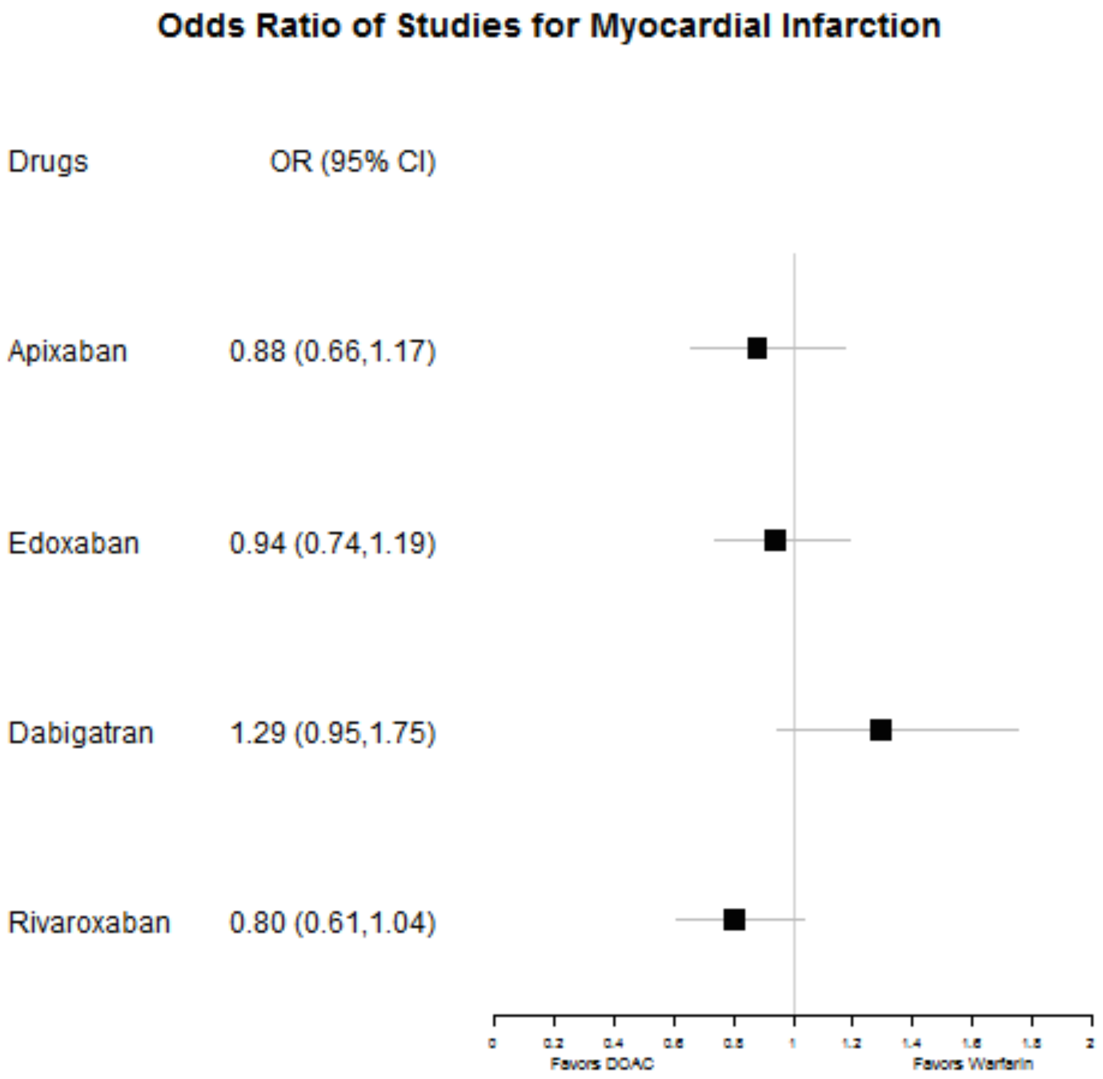
Odds Ratio of Studies for Myocardial Infarction
Drugs
OR (95% CI)
Apixaban
0.88 (0.66,1.17)
Edoxaban
0.94 (0.74,1.19)
Dabigatran
1.29 (0.95,1.75)
Rivaroxaban
0.80 (0.61,1.04)
0
0.2
0.4
0.6
0.8
1
1.2
1.4
1.6
1.8
2
Favors DOAC
Favors Warfarin

Safety

Data Used

STUDY	Drug	Sample Size	Major Bleeding	Fatal Bleeding	Intracranial Bleeding	Gastrointestinal Bleeding
**ARISTOTLE**	Apixaban	9088	327	34	52	105
**ENGAGE AF-TIMI 48 (high dose)**	Edoxaban	7012	418	32	61	232
**RE-LY - 150**	Dabigatran	6076	399		38	188
**ROCKET AF**	Rivaroxaban	7111	395	27	55	224
**ARISTOTLE**	Warfarin	9052	462	55	122	119
**ENGAGE AF-TIMI 48 (high dose)**	Warfarin	7012	524	59	132	190
**RE-LY - 150**	Warfarin	6022	421		90	126
**ROCKET AF**	Warfarin	7125	386	55	84	154

Major bleeding

```
Generalized linear mixed model fit by maximum likelihood (Adaptive Gauss-Hermite
Quadrature, nAGQ = 7) ['glmerMod']
 Family: binomial  ( logit )
Formula: cbind(ADEs[, i], nADEs[, i]) ~ as.factor(arm) + as.factor(id) +      (-1 + arm | id)

    AIC      BIC   logLik deviance df.resid
   24.0     24.5     -6.0     12.0        2

Scaled residuals:
    Min       1Q   Median       3Q      Max
-0.55360 -0.30118 -0.00809  0.30982  0.49351

Random effects:
 Groups Name Variance Std.Dev.
 id     arm  0.01477  0.1215
Number of obs: 8, groups:  id, 4

Fixed effects:
                Estimate Std. Error z value Pr(>|z|)
(Intercept)     -2.94592    0.04901  -60.11  < 2e-16 ***
as.factor(arm)1 -0.16368    0.07060   -2.32   0.0204 *
as.factor(id)2   0.42121    0.06342    6.64 3.10e-11 ***
as.factor(id)3   0.36998    0.07007    5.28 1.29e-07 ***
as.factor(id)4   0.11146    0.07467    1.49   0.1355
---
Signif. codes:  0 '***' 0.001 '**' 0.01 '*' 0.05 '.' 0.1 ' ' 1

Correlation of Fixed Effects:
            (Intr) as.()1 as.()2 as.()3
as.fctr(r)1 -0.165
as.fctr(d)2 -0.719  0.008
as.fctr(d)3 -0.720 -0.013  0.522
as.fctr(d)4 -0.717 -0.018  0.510  0.561
```

Odds Ratio of Studies for Major Bleeding

Drugs	OR (95% CI)
Apixaban	0.69 (0.60,0.80)
Edoxaban	0.78 (0.68,0.89)
Dabigatran	0.94 (0.82,1.08)
Rivaroxaban	1.03 (0.89,1.19)

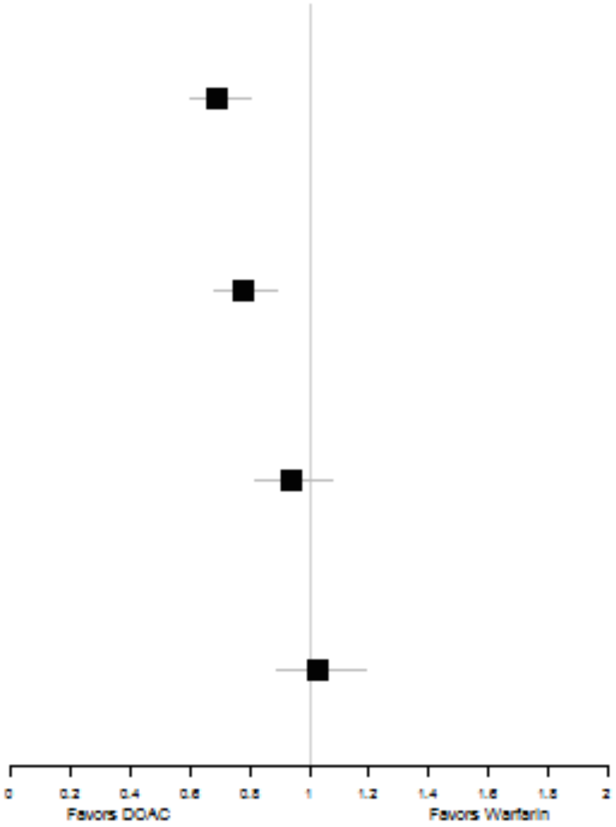

Fatal bleeding

```
Generalized linear mixed model fit by maximum likelihood (Adaptive Gauss-Hermite
Quadrature, nAGQ = 7) ['glmerMod']
 Family: binomial  ( logit )
Formula: cbind(ADEs[, i], nADEs[, i]) ~ as.factor(arm) + as.factor(id) +     (-1 + arm | id)

    AIC      BIC   logLik deviance df.resid
   10.5      9.5     -0.3      0.5        1

Scaled residuals:
    Min      1Q  Median      3Q     Max
-0.3823 -0.2467 -0.0067  0.2225  0.4188

Random effects:
 Groups Name Variance Std.Dev.
 id     arm  0        0
Number of obs: 6, groups:  id, 3

Fixed effects:
                Estimate Std. Error z value Pr(>|z|)
(Intercept)      -5.0552     0.1159  -43.63  < 2e-16 ***
as.factor(arm)1  -0.6014     0.1295   -4.65 3.39e-06 ***
as.factor(id)2    0.2807     0.1495    1.88   0.0605 .
as.factor(id)4    0.1605     0.1535    1.05   0.2958
---
Signif. codes:  0 '***' 0.001 '**' 0.01 '*' 0.05 '.' 0.1 ' ' 1

Correlation of Fixed Effects:
            (Intr) as.()1 as.()2
as.fctr(r)1 -0.398
as.fctr(d)2 -0.652  0.000
as.fctr(d)4 -0.635  0.001  0.492
```

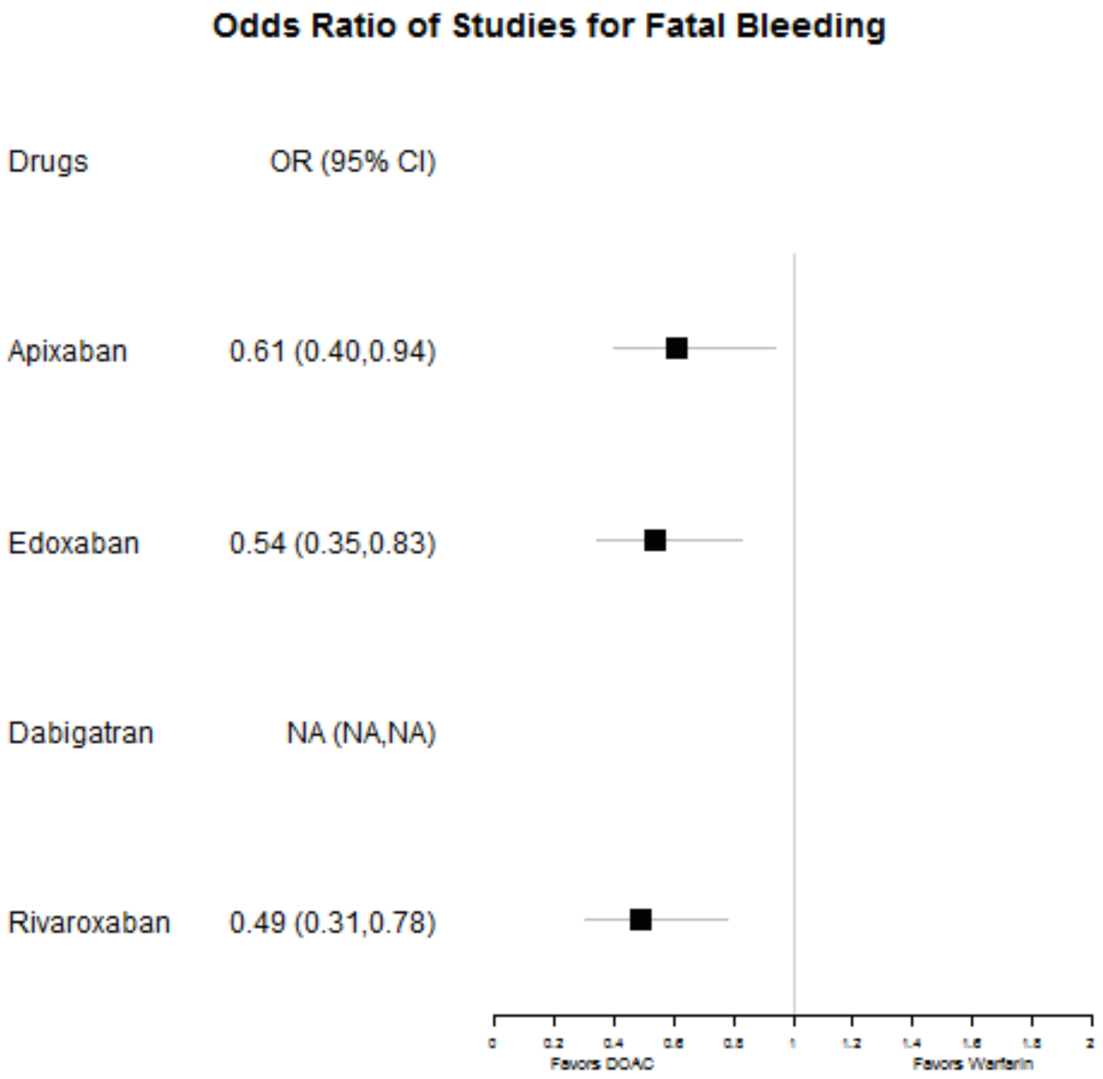
Odds Ratio of Studies for Fatal Bleeding
Drugs
OR (95% CI)
Apixaban
0.61 (0.40,0.94)
Edoxaban
0.54 (0.35,0.83)
Dabigatran
NA (NA,NA)
Rivaroxaban
0.49 (0.31,0.78)
Favors DOAC
Favors Warfarin

Intracranial bleeding

Generalized linear mixed model fit by maximum likelihood (Adaptive Gauss-Hermite Quadrature, nAGQ = 7) ['glmerMod']
Family: binomial (logit)
Formula: cbind(ADEs[, i], nADEs[, i]) ~ as.factor(arm) + as.factor(id) + (-1 + arm | id)

```
   AIC      BIC   logLik deviance df.resid
  16.3     16.8    -2.2      4.3        2

Scaled residuals:
    Min      1Q  Median      3Q     Max
-1.0383 -0.5899 -0.0324  0.4117  1.4954

Random effects:
 Groups Name Variance Std.Dev.
 id     arm  0        0
Number of obs: 8, groups:  id, 4

Fixed effects:
                Estimate Std. Error z value Pr(>|z|)
(Intercept)     -4.33169    0.08114  -53.38  < 2e-16 ***
as.factor(arm)1 -0.74160    0.08522   -8.70  < 2e-16 ***
as.factor(id)2   0.36502    0.10523    3.47 0.000522 ***
as.factor(id)3   0.10006    0.11712    0.85 0.392905
as.factor(id)4   0.01690    0.11438    0.15 0.882515
---
Signif. codes:  0 ‘***’ 0.001 ‘**’ 0.01 ‘*’ 0.05 ‘.’ 0.1 ‘ ’ 1

Correlation of Fixed Effects:
            (Intr) as.()1 as.()2 as.()3
as.fctr(r)1 -0.343
as.fctr(d)2 -0.680  0.000
as.fctr(d)3 -0.611 -0.001  0.471
as.fctr(d)4 -0.626  0.001  0.483  0.434
```

Odds Ratio of Studies for Intracranial Bleeding

Drugs	OR (95% CI)
Apixaban	0.42 (0.30,0.58)
Edoxaban	0.46 (0.34,0.62)
Dabigatran	0.41 (0.28,0.60)
Rivaroxaban	0.65 (0.46,0.91)

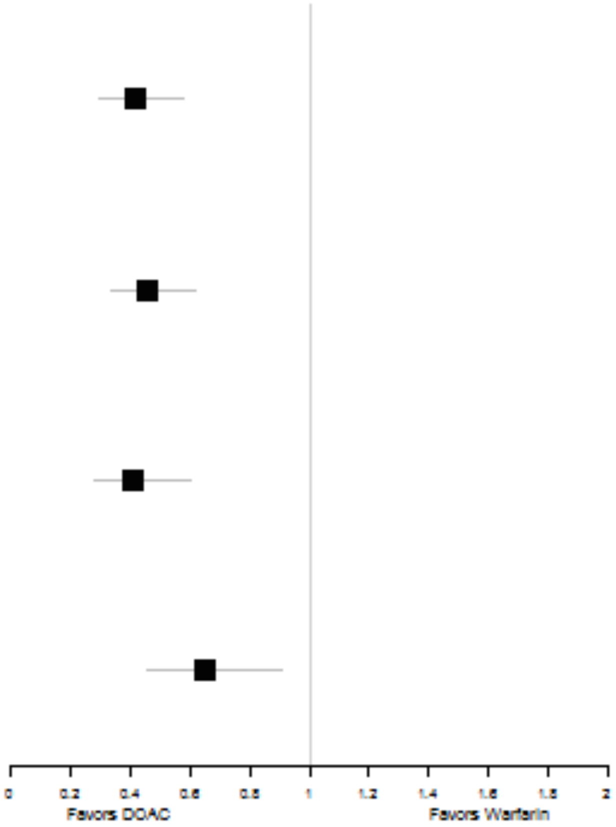

Gastrointestinal bleeding

```
Generalized linear mixed model fit by maximum likelihood (Adaptive Gauss-Hermite
Quadrature, nAGQ = 7) ['glmerMod']
 Family: binomial  ( logit )
Formula: cbind(ADEs[, i], nADEs[, i]) ~ as.factor(arm) + as.factor(id) +      (-1 + arm | id)

    AIC      BIC   logLik deviance df.resid
   23.1     23.6     -5.6     11.1        2

Scaled residuals:
    Min       1Q   Median       3Q      Max
-1.01639 -0.49079  0.00631  0.42784  1.06326

Random effects:
 Groups Name Variance Std.Dev.
 id     arm  0.0149   0.1221
Number of obs: 8, groups:  id, 4

Fixed effects:
             Estimate Std. Error z value Pr(>|z|)
(Intercept)     -4.41646    0.11807  -37.41  < 2e-16 ***
as.factor(arm)1  0.22908    0.08423    2.72 0.006535 **
as.factor(id)2   0.83015    0.12798    6.49 8.79e-11 ***
as.factor(id)3   0.61827    0.16332    3.79 0.000153 ***
as.factor(id)4   0.64286    0.15787    4.07 4.66e-05 ***
---
Signif. codes:  0 '***' 0.001 '**' 0.01 '*' 0.05 '.' 0.1 ' ' 1

Correlation of Fixed Effects:
          (Intr) as.()1 as.()2 as.()3
as.fctr(r)1 -0.304
as.fctr(d)2 -0.850  0.129
as.fctr(d)3 -0.856  0.118  0.742
as.fctr(d)4 -0.871  0.132  0.753  0.797
```

Odds Ratio of Studies for Gastrointestinal Bleeding

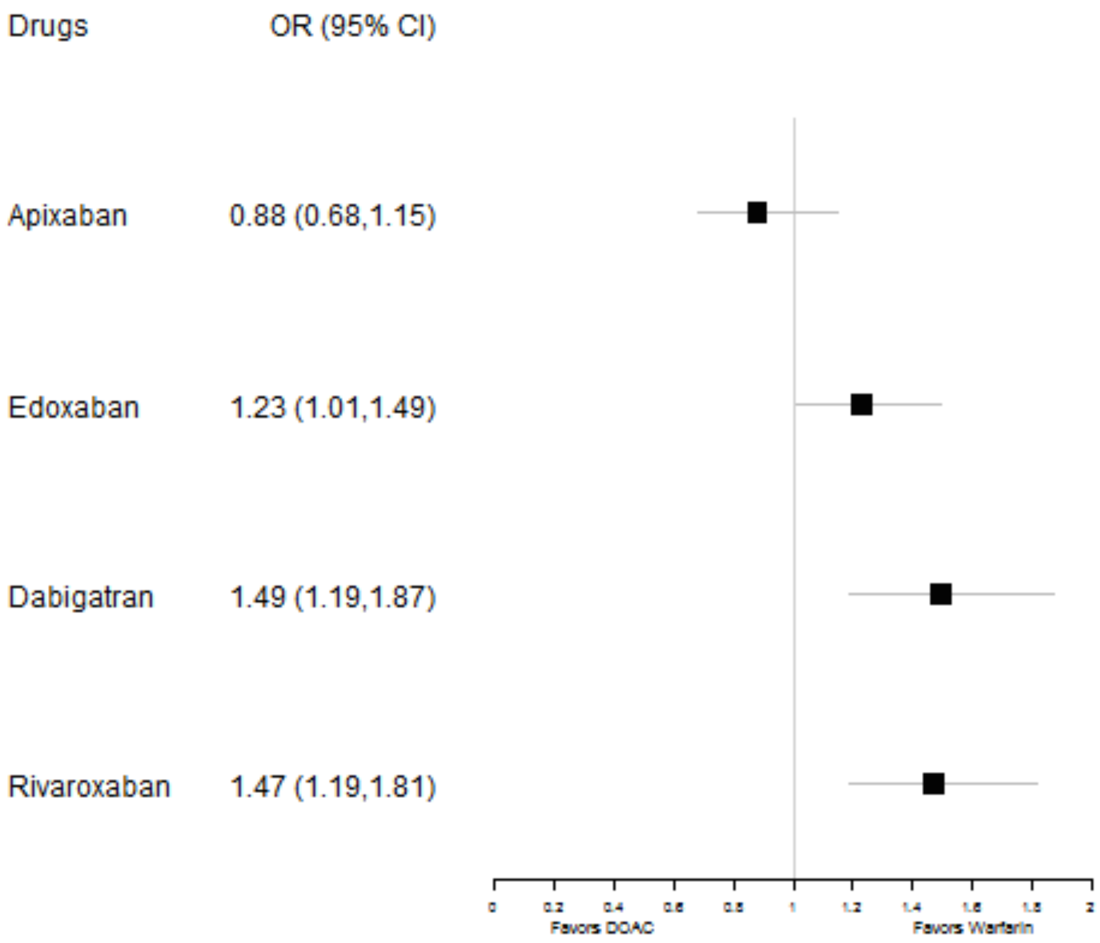

Results of Meta-analyses: Venous thromboembolism

Efficacy

Data Used

Trial	drug	Sample size	Recurrent VTE and Related Death (PO)	Death from Any Cause	Recurrent DVT	Fatal PE
**AMPLIFY**	Apixaban	2609	59	41	20	1
**EINSTEIN DVT**	Rivaroxaban	1731	36	38	14	1
**Hokusai-VTE**	Edoxaban	4118	130	132	57	4
**RE-COVER**	Dabigatran (RCI)	1274	30	21	16	
**RECOVER II**	Dabigatran (RCII)	1279	30	25	25	
**AMPLIFY**	Warfarin	2635	71	52	33	2
**EINSTEIN DVT**	Warfarin	1718	51	49	28	0.5
**Hokusai-VTE**	Warfarin	4122	146	126	63	3
**RE-COVER**	Warfarin	1265	27	21	18	
**RECOVER II**	Warfarin	1289	28	25	17	
**Trial**	**drug**	**Recurrent PE (Non-Fatal)**	**Net Clinical Benefit**	**Myocardial Infarction**	**All death Sample size**	**MIss**
**AMPLIFY**	Apixaban	27	73	4	2676	2609
**EINSTEIN DVT**	Rivaroxaban	20	51	5	1731	1731
**Hokusai-VTE**	Edoxaban	49	120		4118	4118
**RE-COVER**	Dabigatran (RCI)	13		4	1274	1274
**RECOVER II**	Dabigatran (RCII)	7		4	1279	1280
**AMPLIFY**	Warfarin	23	118	2	2689	2635
**EINSTEIN DVT**	Warfarin	18	73	1	1718	1718
**Hokusai-VTE**	Warfarin	59	144		4122	4122
**RE-COVER**	Warfarin	7		2	1265	1265
**RECOVER II**	Warfarin	13		2	1289	1288

Recurrent VTE and related death

```
Generalized linear mixed model fit by maximum likelihood (Adaptive Gauss-Hermite
Quadrature, nAGQ = 7) ['glmerMod']
 Family: binomial  ( logit )
Formula: cbind(ADEs[, i], nADEs[, i]) ~ as.factor(arm) + as.factor(id) +      (-1 + arm | id)

    AIC      BIC   logLik deviance df.resid
   16.6     18.7     -1.3      2.6        3

Scaled residuals:
    Min      1Q  Median      3Q     Max
-0.7858 -0.4514  0.0014  0.4746  0.7425

Random effects:
 Groups Name Variance Std.Dev.
 id     arm  0        0
Number of obs: 10, groups:  id, 5

Fixed effects:
                Estimate Std. Error z value Pr(>|z|)
(Intercept)     -3.61083    0.09681  -37.30  < 2e-16 ***
as.factor(arm)1 -0.12721    0.08244   -1.54  0.12284
as.factor(id)2   0.01838    0.14029    0.13  0.89579
as.factor(id)3   0.31024    0.10788    2.88  0.00403 **
as.factor(id)4  -0.10104    0.16074   -0.63  0.52963
as.factor(id)5  -0.09533    0.15978   -0.60  0.55074
---
Signif. codes:  0 ‘***’ 0.001 ‘**’ 0.01 ‘*’ 0.05 ‘.’ 0.1 ‘ ’ 1

Correlation of Fixed Effects:
            (Intr) as.()1 as.()2 as.()3 as.()4
as.fctr(r)1 -0.398
as.fctr(d)2 -0.580 -0.003
as.fctr(d)3 -0.754 -0.002  0.521
as.fctr(d)4 -0.506 -0.002  0.350  0.455
as.fctr(d)5 -0.510  0.000  0.352  0.458  0.307
```

Odds Ratio of Studies for Recurrent VTE and Related Death (PO)

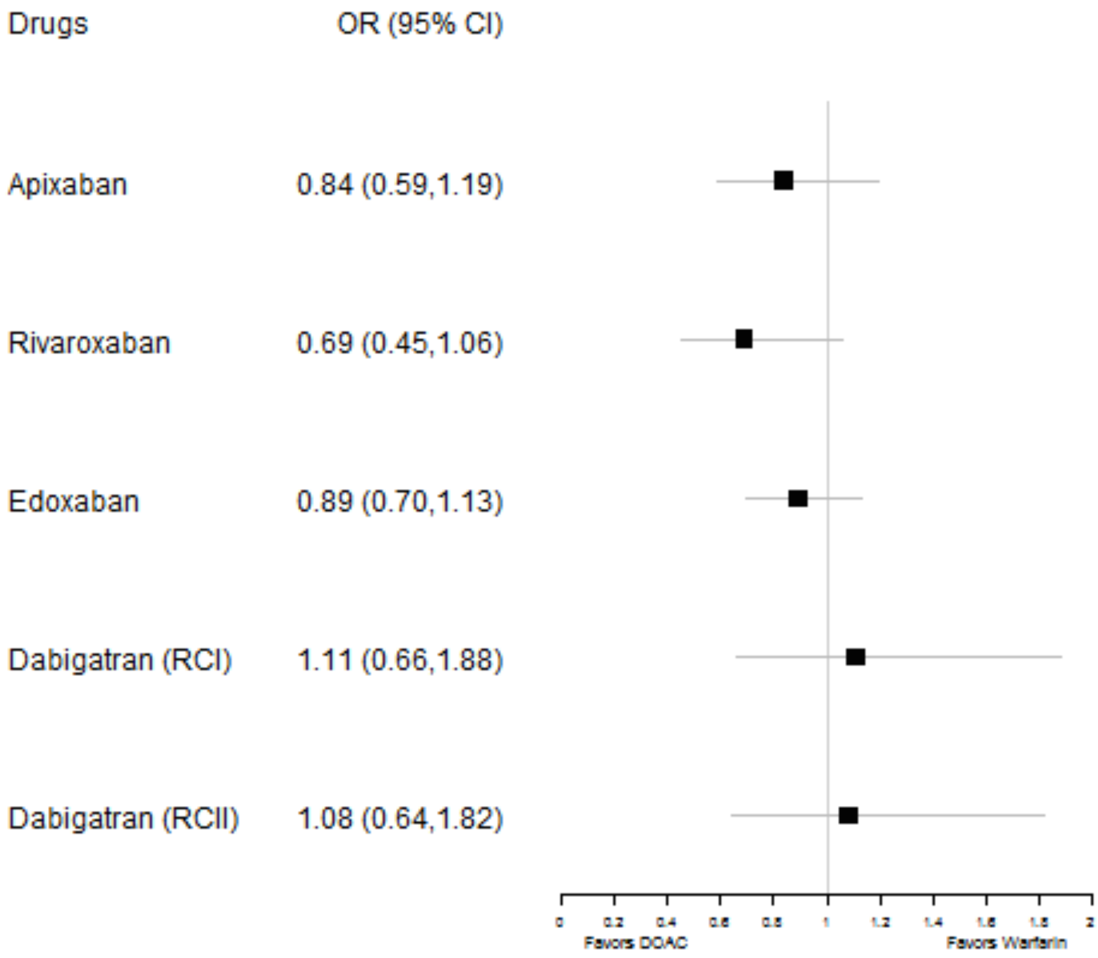

Death from any cause

```
Generalized linear mixed model fit by maximum likelihood (Adaptive Gauss-Hermite
Quadrature, nAGQ = 7) ['glmerMod']
 Family: binomial  ( logit )
Formula: cbind(ADEs[, i], nADEs[, i]) ~ as.factor(arm) + as.factor(id) +      (-1 + arm | id)

    AIC      BIC   logLik deviance df.resid
   16.5     18.6     -1.2      2.5        3

Scaled residuals:
    Min       1Q   Median       3Q      Max
-0.67762 -0.49102  0.00163  0.47825  0.66054

Random effects:
 Groups Name Variance Std.Dev.
 id     arm  0        0
Number of obs: 10, groups:  id, 5

Fixed effects:
               Estimate Std. Error z value Pr(>|z|)
(Intercept)     -4.00724    0.11295  -35.48  < 2e-16 ***
as.factor(arm)1 -0.06172    0.08805   -0.70    0.483
as.factor(id)2   0.38338    0.15078    2.54    0.011 *
as.factor(id)3   0.60565    0.12225    4.95 7.25e-07 ***
as.factor(id)4  -0.04743    0.18749   -0.25    0.800
as.factor(id)5   0.11833    0.17703    0.67    0.504
---
Signif. codes:  0 '***' 0.001 '**' 0.01 '*' 0.05 '.' 0.1 ' ' 1

Correlation of Fixed Effects:
            (Intr) as.()1 as.()2 as.()3 as.()4
as.fctr(r)1 -0.377
as.fctr(d)2 -0.642 -0.002
as.fctr(d)3 -0.792 -0.001  0.594
as.fctr(d)4 -0.516 -0.001  0.387  0.477
as.fctr(d)5 -0.547  0.000  0.410  0.506  0.330
```

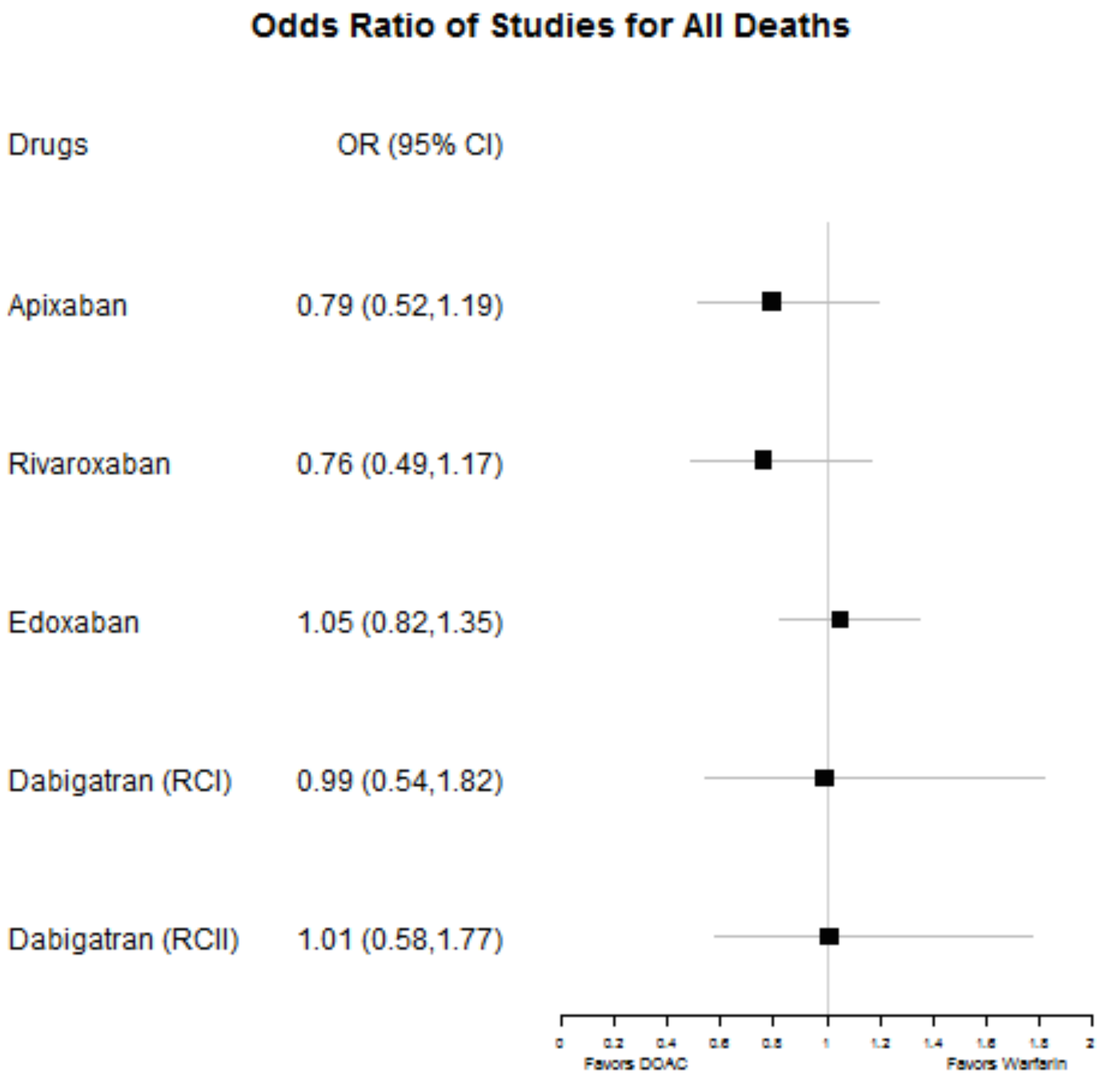
Odds Ratio of Studies for All Deaths
Drugs
OR (95% CI)
Apixaban
0.79 (0.52,1.19)
Rivaroxaban
0.76 (0.49,1.17)
Edoxaban
1.05 (0.82,1.35)
Dabigatran (RCI)
0.99 (0.54,1.82)
Dabigatran (RCII)
1.01 (0.58,1.77)
Favors DOAC
Favors Warfarin

Recurrent DVT

```
Generalized linear mixed model fit by maximum likelihood (Adaptive Gauss-Hermite
Quadrature, nAGQ = 7) ['glmerMod']
 Family: binomial  ( logit )
Formula: cbind(ADEs[, i], nADEs[, i]) ~ as.factor(arm) + as.factor(id) +      (-1 + arm | id)

    AIC      BIC   logLik deviance df.resid
   21.6     23.7     -3.8      7.6        3

Scaled residuals:
    Min       1Q   Median       3Q      Max
-1.26411 -0.68339  0.00542  0.63531  1.38932

Random effects:
 Groups Name Variance  Std.Dev.
 id     arm  2.463e-18 1.569e-09
Number of obs: 10, groups:  id, 5

Fixed effects:
               Estimate Std. Error z value Pr(>|z|)
(Intercept)     -4.4955     0.1481 -30.356   <2e-16 ***
as.factor(arm)1  -0.1872     0.1185  -1.579   0.1143
as.factor(id)2    0.1893     0.2078   0.911   0.3622
as.factor(id)3    0.3703     0.1659   2.232   0.0256 *
as.factor(id)4    0.2855     0.2211   1.292   0.1965
as.factor(id)5    0.4878     0.2080   2.345   0.0190 *
---
Signif. codes:  0 '***' 0.001 '**' 0.01 '*' 0.05 '.' 0.1 ' ' 1

Correlation of Fixed Effects:
            (Intr) as.()1 as.()2 as.()3 as.()4
as.fctr(r)1 -0.362
as.fctr(d)2 -0.619 -0.003
as.fctr(d)3 -0.775 -0.002  0.553
as.fctr(d)4 -0.581 -0.002  0.415  0.520
as.fctr(d)5 -0.619 -0.001  0.441  0.552  0.415
```

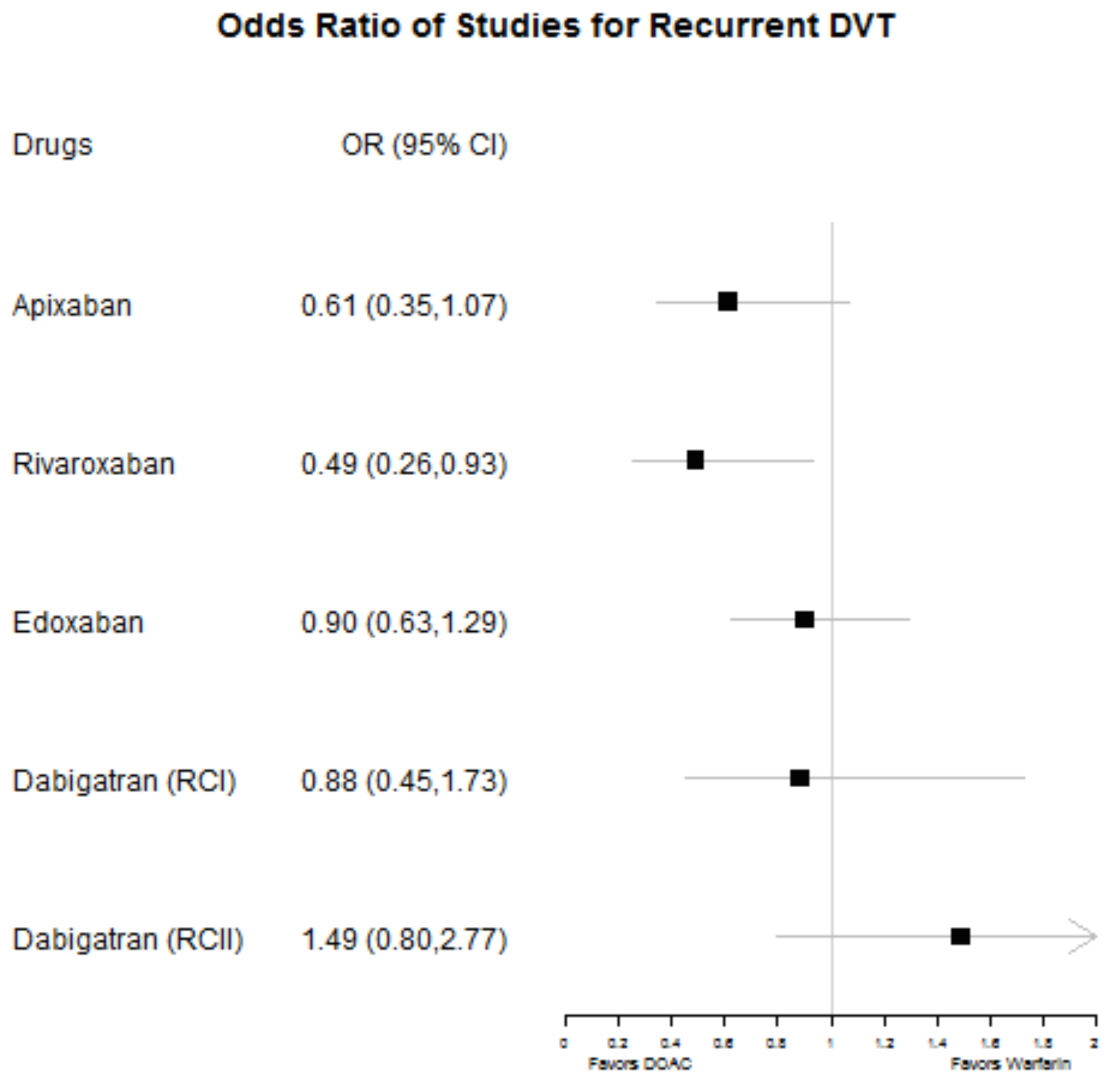

Odds Ratio of Studies for Recurrent DVT
Drugs
OR (95% CI)
Apixaban
0.61 (0.35,1.07)
Rivaroxaban
0.49 (0.26,0.93)
Edoxaban
0.90 (0.63,1.29)
Dabigatran (RCI)
0.88 (0.45,1.73)
Dabigatran (RCII)
1.49 (0.80,2.77)
0
0.2
0.4
0.6
0.8
1
1.2
1.4
1.6
1.8
2
Favors DOAC
Favors Warfarin

Fatal PE

```
Generalized linear mixed model fit by maximum likelihood (Adaptive Gauss-Hermite
Quadrature, nAGQ = 7) ['glmerMod']
 Family: binomial  ( logit )
Formula: cbind(ADEs[, i], nADEs[, i]) ~ as.factor(arm) + as.factor(id) +      (-1 + arm | id)

    AIC      BIC   logLik deviance df.resid
   11.8     10.7     -0.9      1.8        1

Scaled residuals:
    Min       1Q   Median       3Q      Max
-0.67240 -0.39577 -0.00441  0.42745  0.61069

Random effects:
 Groups Name Variance Std.Dev.
 id     arm  0        0
Number of obs: 6, groups:  id, 3

Fixed effects:
               Estimate Std. Error z value Pr(>|z|)
(Intercept)      -7.5620     0.6648 -11.375   <2e-16 ***
as.factor(arm)1   0.1851     0.6057   0.306    0.760
as.factor(id)2   -0.6807     1.1549  -0.589    0.556
as.factor(id)3    0.3952     0.6903   0.573    0.567
---
Signif. codes:  0 '***' 0.001 '**' 0.01 '*' 0.05 '.' 0.1 ' ' 1

Correlation of Fixed Effects:
            (Intr) as.()1 as.()2
as.fctr(r)1 -0.495
as.fctr(d)2 -0.433 -0.002
as.fctr(d)3 -0.726 -0.002  0.418
```

Odds Ratio of Studies for Fatal PE

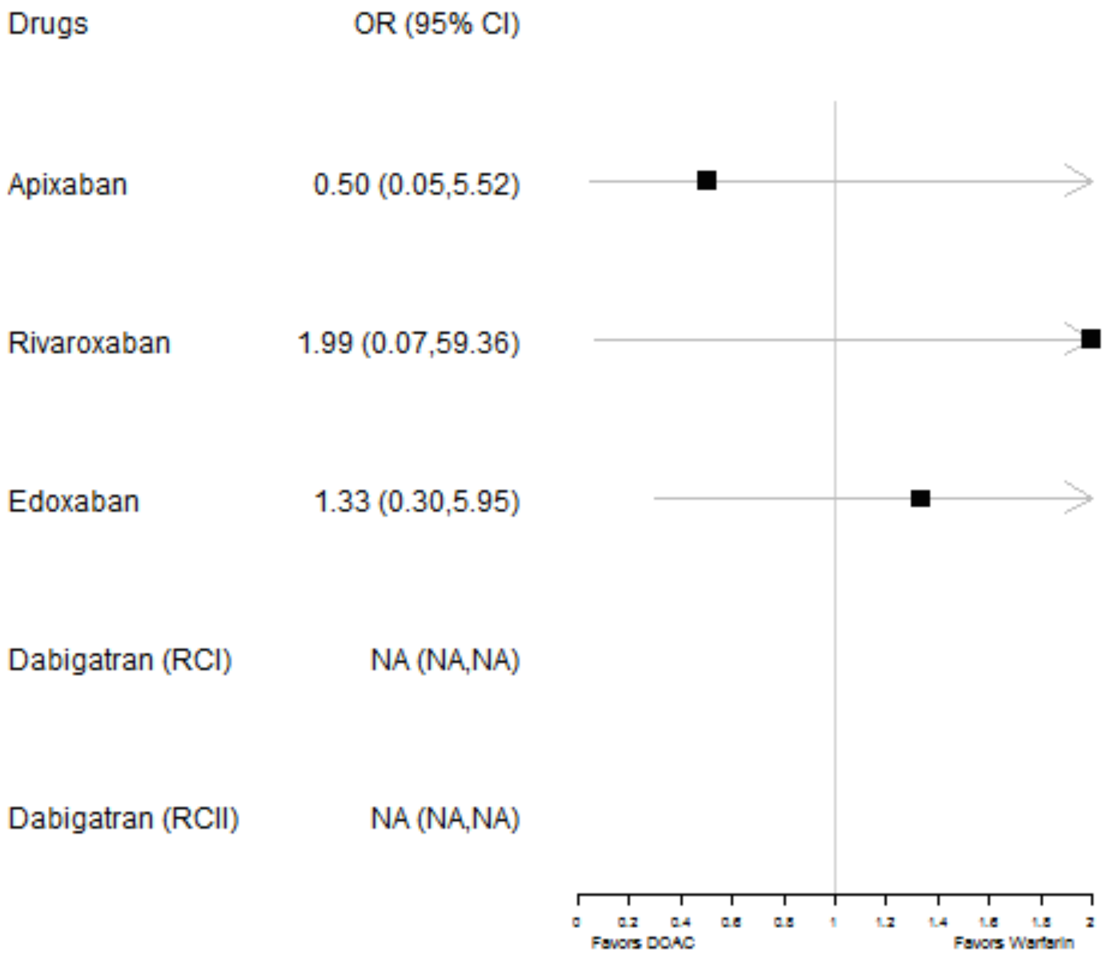

Recurrent PE (non-fatal)

```
Generalized linear mixed model fit by maximum likelihood (Adaptive Gauss-Hermite
Quadrature, nAGQ = 7) ['glmerMod']
 Family: binomial  ( logit )
Formula: cbind(ADEs[, i], nADEs[, i]) ~ as.factor(arm) + as.factor(id) +      (-1 + arm | id)

   AIC      BIC   logLik deviance df.resid
  18.9     21.0     -2.5      4.9        3

Scaled residuals:
    Min       1Q   Median       3Q      Max
-0.98675 -0.55035  0.00176  0.54609  0.99930

Random effects:
 Groups Name Variance Std.Dev.
 id     arm  0        0
Number of obs: 10, groups:  id, 5

Fixed effects:
             Estimate Std. Error z value Pr(>|z|)
(Intercept)     -4.62701    0.15588 -29.682   <2e-16 ***
as.factor(arm)1 -0.03288    0.13094  -0.251   0.8017
as.factor(id)2   0.14621    0.21634   0.676   0.4992
as.factor(id)3   0.32188    0.17197   1.872   0.0613 .
as.factor(id)4  -0.19251    0.26569  -0.725   0.4687
as.factor(id)5  -0.20408    0.26568  -0.768   0.4424
---
Signif. codes:  0 ‘***’ 0.001 ‘**’ 0.01 ‘*’ 0.05 ‘.’ 0.1 ‘ ’ 1

Correlation of Fixed Effects:
          (Intr) as.()1 as.()2 as.()3 as.()4
as.fctr(r)1 -0.411
as.fctr(d)2 -0.598 -0.003
as.fctr(d)3 -0.753 -0.002  0.543
as.fctr(d)4 -0.487 -0.002  0.351  0.442
as.fctr(d)5 -0.487  0.000  0.351  0.442  0.286
```

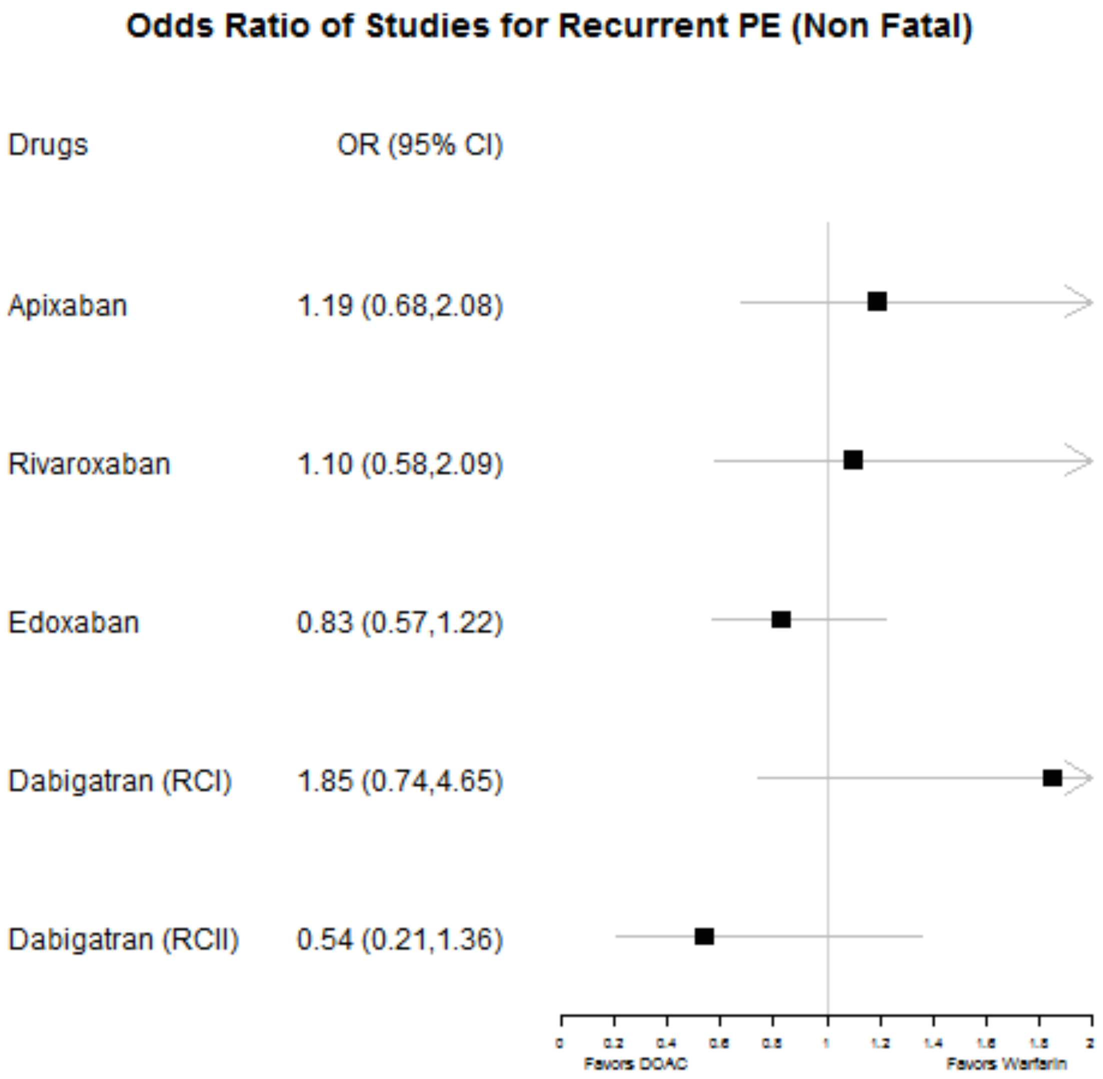
Odds Ratio of Studies for Recurrent PE (Non Fatal)
Drugs
OR (95% CI)
Apixaban
1.19 (0.68,2.08)
Rivaroxaban
1.10 (0.58,2.09)
Edoxaban
0.83 (0.57,1.22)
Dabigatran (RCI)
1.85 (0.74,4.65)
Dabigatran (RCII)
0.54 (0.21,1.36)
0
0.2
0.4
0.6
0.8
1
1.2
1.4
1.6
1.8
2
Favors DOAC
Favors Warfarin

Net clinical benefit

```
Generalized linear mixed model fit by maximum likelihood (Adaptive Gauss-Hermite
Quadrature, nAGQ = 7) ['glmerMod']
 Family: binomial  ( logit )
Formula: cbind(ADEs[, i], nADEs[, i]) ~ as.factor(arm) + as.factor(id) +     (-1 + arm | id)

    AIC      BIC   logLik deviance df.resid
   12.4     11.4     -1.2      2.4        1

Scaled residuals:
    Min       1Q   Median       3Q      Max
-0.81205 -0.59411 -0.01502  0.56822  0.83814

Random effects:
 Groups Name Variance Std.Dev.
 id     arm  0        0
Number of obs: 6, groups:  id, 3

Fixed effects:
               Estimate Std. Error z value Pr(>|z|)
(Intercept)     -3.12556    0.08214  -38.05  < 2e-16 ***
as.factor(arm)1 -0.32596    0.08561   -3.81  0.00014 ***
as.factor(id)2  -0.01208    0.11752   -0.10  0.91813
as.factor(id)3  -0.13217    0.09672   -1.37  0.17178
---
Signif. codes:  0 '***' 0.001 '**' 0.01 '*' 0.05 '.' 0.1 ' ' 1

Correlation of Fixed Effects:
            (Intr) as.()1 as.()2
as.fctr(r)1 -0.440
as.fctr(d)2 -0.562 -0.003
as.fctr(d)3 -0.684 -0.001  0.478
```

Odds Ratio of Studies for Net Clinical Benefit

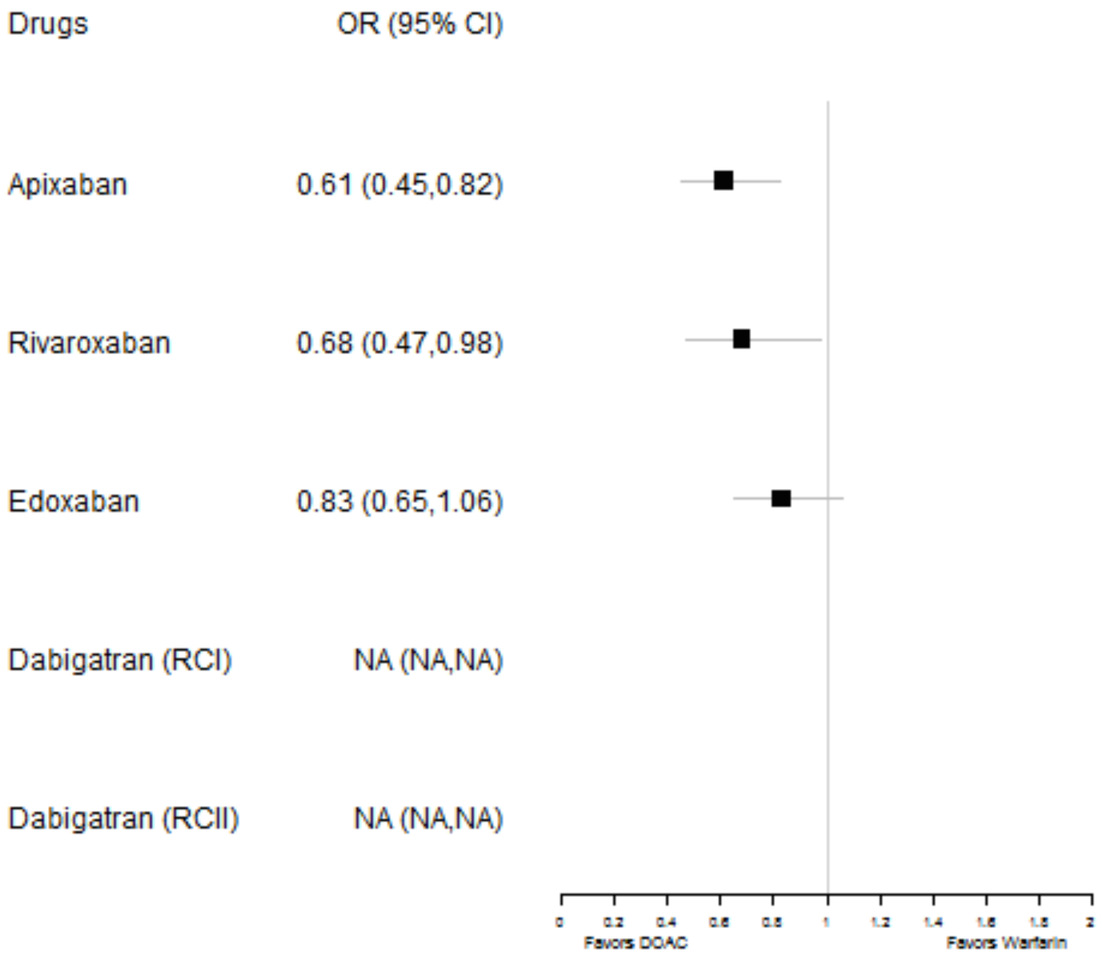

Myocardial infarction

```
Generalized linear mixed model fit by maximum likelihood (Adaptive Gauss-Hermite
Quadrature, nAGQ = 7) ['glmerMod']
 Family: binomial  ( logit )
Formula: cbind(ADEs[, i], nADEs[, i]) ~ as.factor(arm) + as.factor(id) +      (-1 + arm | id)

    AIC      BIC   logLik deviance df.resid
   12.6     13.1     -0.3      0.6        2

Scaled residuals:
    Min       1Q   Median       3Q      Max
-0.56129 -0.11978  0.03198  0.18622  0.35898

Random effects:
 Groups Name Variance Std.Dev.
 id     arm  0        0
Number of obs: 8, groups:  id, 4

Fixed effects:
              Estimate Std. Error z value Pr(>|z|)
(Intercept)      -7.3101     0.5174 -14.130   <2e-16 ***
as.factor(arm)1   0.8893     0.4494   1.979   0.0479 *
as.factor(id)2    0.4161     0.5778   0.720   0.4715
as.factor(id)4    0.7232     0.5779   1.251   0.2108
as.factor(id)5    0.7146     0.5779   1.236   0.2163
---
Signif. codes:  0 '***' 0.001 '**' 0.01 '*' 0.05 '.' 0.1 ' ' 1

Correlation of Fixed Effects:
           (Intr) as.()1 as.()2 as.()4
as.fctr(r)1 -0.614
as.fctr(d)2 -0.557 -0.003
as.fctr(d)4 -0.557 -0.002  0.500
as.fctr(d)5 -0.558  0.000  0.500  0.500
```

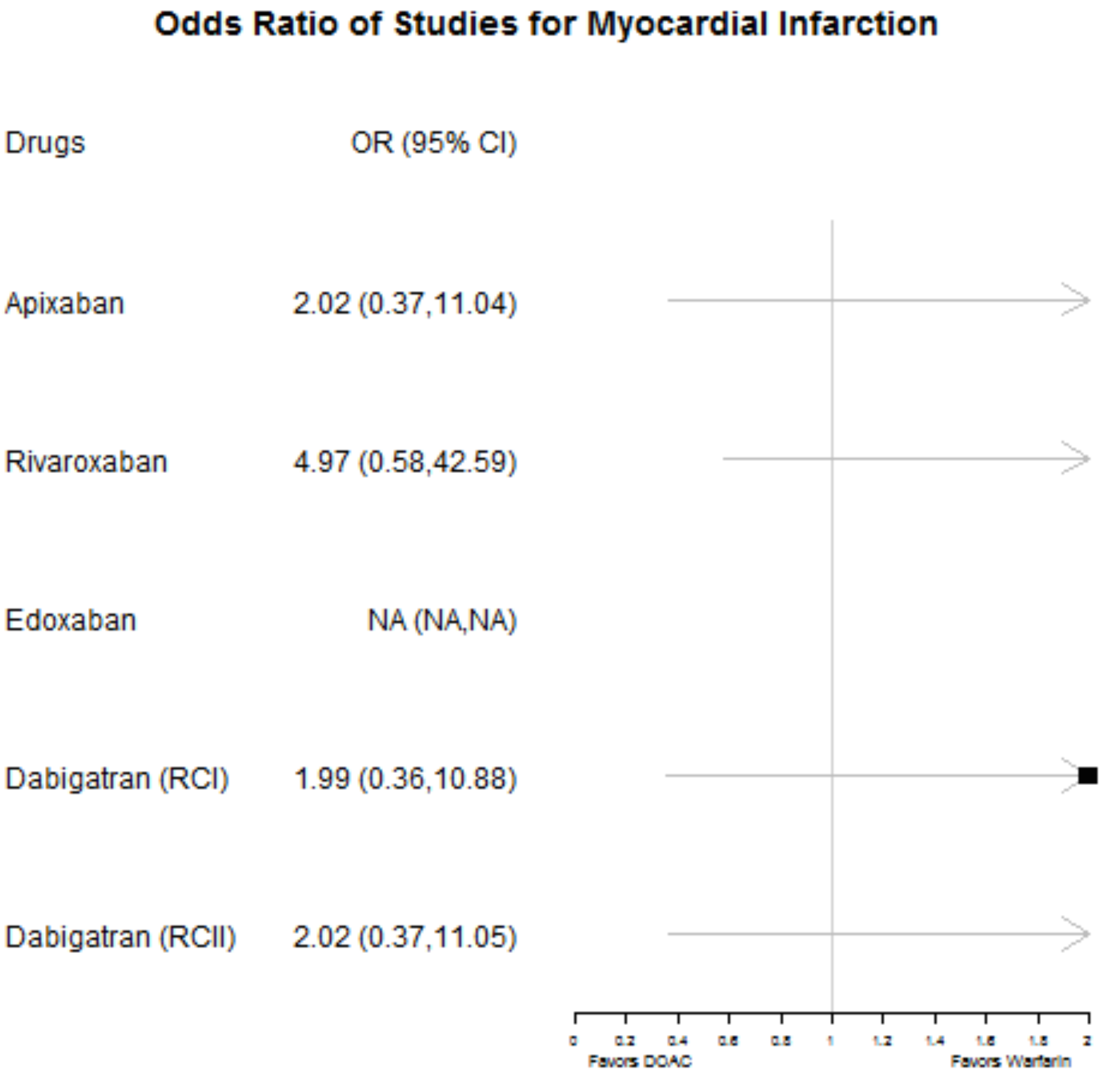
Odds Ratio of Studies for Myocardial Infarction
Drugs
OR (95% CI)
Apixaban
2.02 (0.37,11.04)
Rivaroxaban
4.97 (0.58,42.59)
Edoxaban
NA (NA,NA)
Dabigatran (RCI)
1.99 (0.36,10.88)
Dabigatran (RCII)
2.02 (0.37,11.05)
0
0.2
0.4
0.6
0.8
1
1.2
1.4
1.6
1.8
2
Favors DOAC
Favors Warfarin

Safety

Data Used

STUDY	Drug	Sample Size	All-cause Mortality	Major Bleeding	Fatal Bleeding	Intracranial Bleeding	Gastrointestinal Bleeding
**RE-COVER**	Dabigatran (RCI)	1273	21	20	1	0	53
**RE-COVER2**	Dabigatran (RCII)	1280	25	15	0	2	48
**EINSTEIN-DVT**	Rivaroxaban	1718	38	14	1		
**AMPLIFY**	Apixaban	2676	41	15	1	3	7
**Hokusai-VTE**	Edoxaban	4118	132	56	2	5	
**RE-COVER**	Warfarin	1266	21	24	1	3	35
**RE-COVER2**	Warfarin	1288	25	22	1	2	33
**EINSTEIN-DVT**	Enoxaparin, Warfarin/acenocoumarol	1711	49	20	5		
**AMPLIFY**	Enoxaparin,Warfarin	2689	52	49	2	6	18
**Hokusai-VTE**	LMWH Warfarin	4122	126	66	10	18	
**STUDY**	Drug	ALT greater than 3xULN	Non-major Relevant Bleeding	Serious Adverse Event	Medication Stopped (ADE)	All-cause Mortality Denominator	ALT greater than 3xULN Denominator
**RE-COVER**	Dabigatran (RCI)	42		165	126	1273	1220
**RE-COVER2**	Dabigatran (RCII)			156	102	1280	
**EINSTEIN-DVT**	Rivaroxaban	25	126	201	85	1718	1680
**AMPLIFY**	Apixaban	50	103	417	150	2676	2601
**Hokusai-VTE**	Edoxaban	81	298	503	41	4118	3901
**RE-COVER**	Warfarin	46		150	102	1266	1199
**RE-COVER2**	Warfarin			153	101	1288	
**EINSTEIN-DVT**	Enoxaparin, Warfarin/acenocoumarol	62	119	233	81	1711	1649
**AMPLIFY**	Enoxaparin,Warfarin	145	215	410	182	2689	2598
**Hokusai-VTE**	LMWH Warfarin	90	368	544	51	4122	3903

Major bleeding

```
Generalized linear mixed model fit by maximum likelihood (Adaptive Gauss-Hermite
Quadrature, nAGQ = 7) ['glmerMod']
 Family: binomial  ( logit )
Formula: cbind(ADEs[, i], nADEs[, i]) ~ as.factor(arm) + as.factor(id) +      (-1 + arm | id)

    AIC      BIC   logLik deviance df.resid
   23.4     25.5     -4.7      9.4        3

Scaled residuals:
    Min       1Q   Median       3Q      Max
-1.31368 -0.29020  0.02406  0.37533  0.94190

Random effects:
 Groups Name Variance Std.Dev.
 id     arm  0.04453  0.211
Number of obs: 10, groups:  id, 5

Fixed effects:
             Estimate Std. Error z value Pr(>|z|)
(Intercept)      -3.8733     0.1771 -21.867  < 2e-16 ***
as.factor(arm)1  -0.4505     0.1591  -2.832  0.00463 **
as.factor(id)2   -0.1633     0.2485  -0.657  0.51101
as.factor(id)3   -0.5424     0.2532  -2.142  0.03217 *
as.factor(id)4   -0.2491     0.2511  -0.992  0.32126
as.factor(id)5   -0.1916     0.2017  -0.950  0.34199
---
Signif. codes:  0 '***' 0.001 '**' 0.01 '*' 0.05 '.' 0.1 ' ' 1

Correlation of Fixed Effects:
          (Intr) as.()1 as.()2 as.()3 as.()4
as.fctr(r)1 -0.216
as.fctr(d)2 -0.665 -0.021
as.fctr(d)3 -0.650 -0.024  0.463
as.fctr(d)4 -0.740 -0.035  0.512  0.498
as.fctr(d)5 -0.804  0.083  0.560  0.550  0.536
```

Odds Ratio of Studies for Major Bleeding

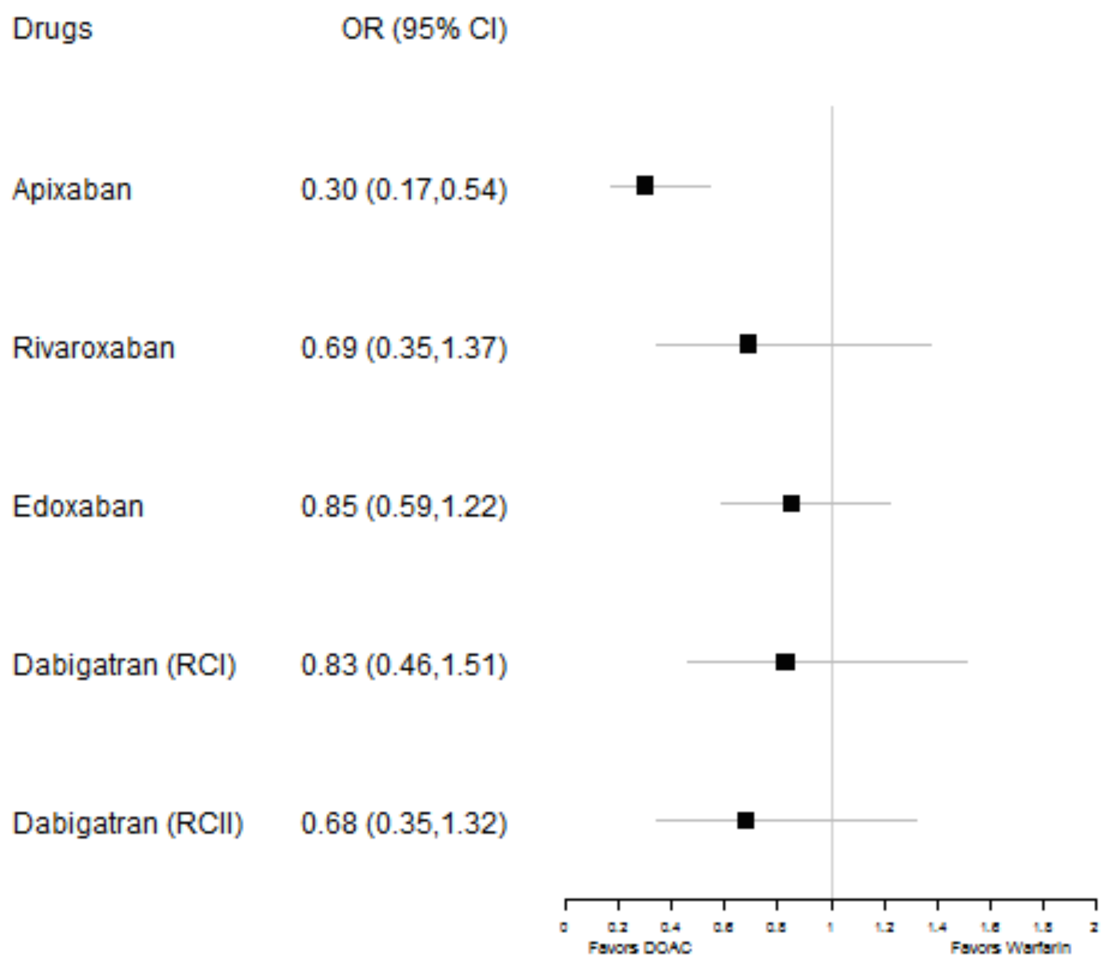

Fatal bleeding

```
Generalized linear mixed model fit by maximum likelihood (Adaptive Gauss-Hermite
Quadrature, nAGQ = 7) ['glmerMod']
 Family: binomial  ( logit )
Formula: cbind(ADEs[, i], nADEs[, i]) ~ as.factor(arm) + as.factor(id) +      (-1 + arm | id)

    AIC      BIC   logLik deviance df.resid
   15.4     17.5     -0.7      1.4        3

Scaled residuals:
     Min       1Q   Median       3Q      Max
-0.44158 -0.28097  0.00517  0.27078  0.81836

Random effects:
 Groups Name Variance Std.Dev.
 id     arm  0        0
Number of obs: 10, groups:  id, 5

Fixed effects:
               Estimate Std. Error z value Pr(>|z|)
(Intercept)     -6.7046     0.7158  -9.366   <2e-16 ***
as.factor(arm)1  -1.2411     0.4844  -2.562   0.0104 *
as.factor(id)2   -0.3025     1.0806  -0.280   0.7795
as.factor(id)3    0.7990     0.8170   0.978   0.3281
as.factor(id)4   -0.3458     0.9132  -0.379   0.7049
as.factor(id)5    0.6136     0.7642   0.803   0.4220
---
Signif. codes:  0 ‘***’ 0.001 ‘**’ 0.01 ‘*’ 0.05 ‘.’ 0.1 ‘ ’ 1

Correlation of Fixed Effects:
            (Intr) as.()1 as.()2 as.()3 as.()4
as.fctr(r)1 -0.153
as.fctr(d)2 -0.647  0.001
as.fctr(d)3 -0.856  0.000  0.567
as.fctr(d)4 -0.766  0.001  0.507  0.671
as.fctr(d)5 -0.915  0.001  0.606  0.802  0.717
```

Odds Ratio of Studies for Fatal Bleeding

Drugs	OR (95% CI)
Apixaban	0.50 (0.05,5.52)
Rivaroxaban	0.20 (0.02,1.71)
Edoxaban	0.20 (0.04,0.91)
Dabigatran (RCI)	0.99 (0.06,15.85)
Dabigatran (RCII)	0.50 (0.02,14.92)

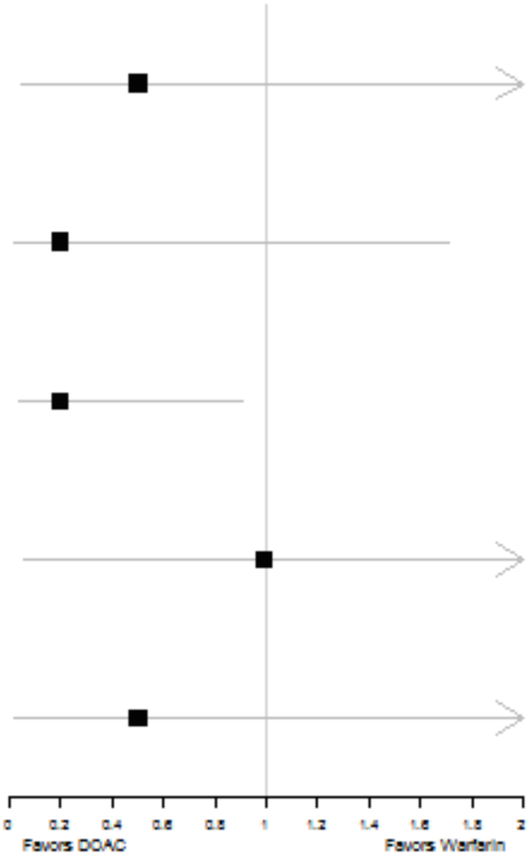

Intracranial bleeding

```
Generalized linear mixed model fit by maximum likelihood (Adaptive Gauss-Hermite
Quadrature, nAGQ = 7) ['glmerMod']
 Family: binomial  ( logit )
Formula: cbind(ADEs[, i], nADEs[, i]) ~ as.factor(arm) + as.factor(id) +      (-1 + arm | id)

    AIC      BIC   logLik deviance df.resid
   13.8     14.3     -0.9      1.8        2

Scaled residuals:
    Min       1Q   Median       3Q      Max
-0.54918 -0.45066  0.01637  0.30424  0.91436

Random effects:
 Groups Name Variance Std.Dev.
 id     arm  0        0
Number of obs: 8, groups:  id, 4

Fixed effects:
              Estimate Std. Error z value Pr(>|z|)
(Intercept)     -6.1996     0.5436 -11.405  < 2e-16 ***
as.factor(arm)1  -1.0163     0.3605  -2.819  0.00482 **
as.factor(id)2   0.1196     0.7326   0.163  0.87027
as.factor(id)4   0.1943     0.6305   0.308  0.75799
as.factor(id)5   0.7057     0.5743   1.229  0.21914
---
Signif. codes:  0 ‘***’ 0.001 ‘**’ 0.01 ‘*’ 0.05 ‘.’ 0.1 ‘ ’ 1

Correlation of Fixed Effects:
            (Intr) as.()1 as.()2 as.()4
as.fctr(r)1 -0.177
as.fctr(d)2 -0.719  0.001
as.fctr(d)4 -0.835  0.001  0.620
as.fctr(d)5 -0.917  0.000  0.680  0.790
```

Odds Ratio of Studies for Intracranial Bleeding

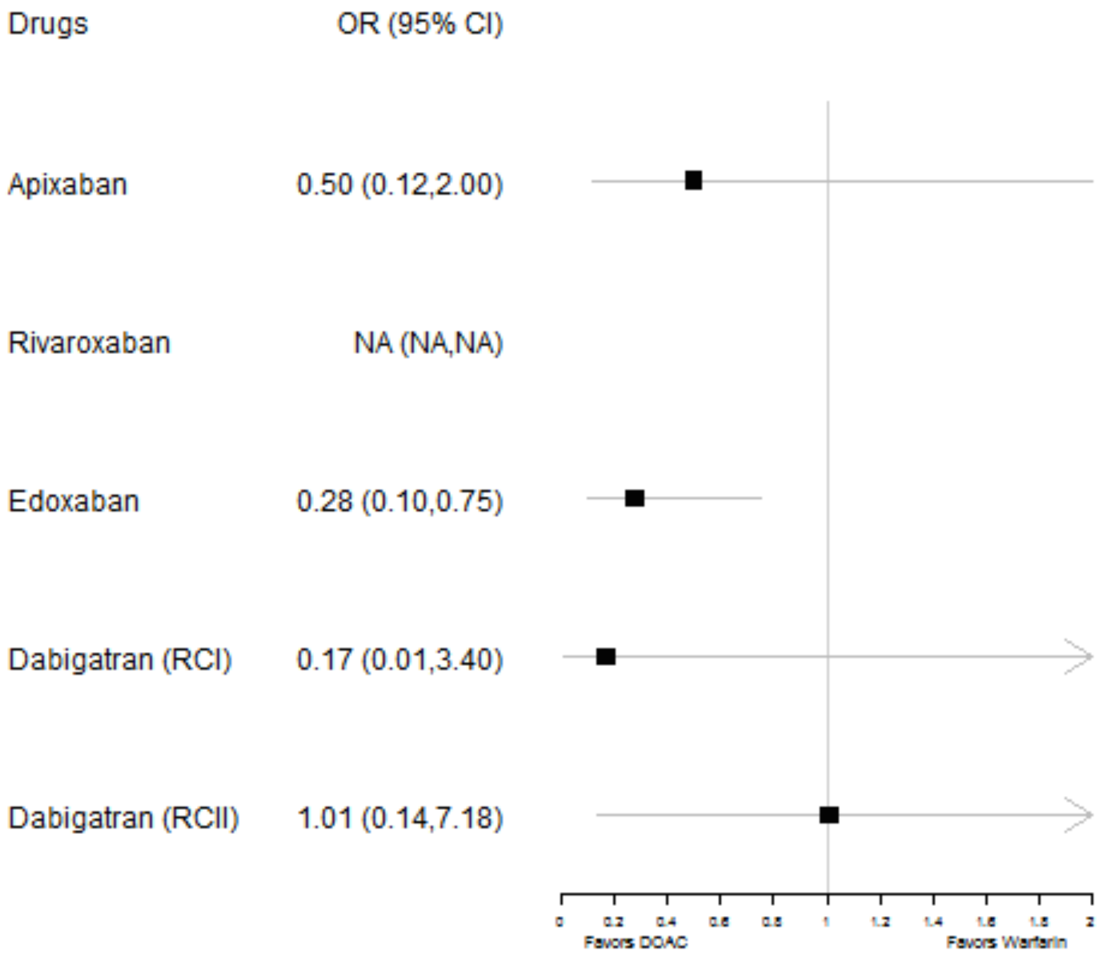

ALT > 3xULN

```
Generalized linear mixed model fit by maximum likelihood (Adaptive Gauss-Hermite
Quadrature, nAGQ = 7) ['glmerMod']
 Family: binomial  ( logit )
Formula: cbind(ADEs[, i], nADEs[, i]) ~ as.factor(arm) + as.factor(id) +      (-1 + arm | id)

    AIC      BIC   logLik deviance df.resid
   24.8     25.3     -6.4     12.8        2

Scaled residuals:
    Min       1Q   Median       3Q      Max
-0.37833 -0.31366 -0.01216  0.24833  0.34724

Random effects:
 Groups Name Variance Std.Dev.
 id     arm  0.1674   0.4092
Number of obs: 8, groups:  id, 4

Fixed effects:
             Estimate Std. Error z value Pr(>|z|)
(Intercept)     -3.17499    0.14458 -21.961  < 2e-16 ***
as.factor(arm)1 -0.57433    0.22651  -2.536  0.01123 *
as.factor(id)3  -0.09717    0.19455  -0.499  0.61743
as.factor(id)4   0.32629    0.16995   1.920  0.05487 .
as.factor(id)5  -0.54432    0.17355  -3.136  0.00171 **
---
Signif. codes:  0 '***' 0.001 '**' 0.01 '*' 0.05 '.' 0.1 ' ' 1

Correlation of Fixed Effects:
           (Intr) as.()1 as.()3 as.()4
as.fctr(r)1 -0.155
as.fctr(d)3 -0.750  0.024
as.fctr(d)4 -0.861  0.079  0.659
as.fctr(d)5 -0.797  0.055  0.593  0.681
```

Odds Ratio of Studies for ALT > 3xULN

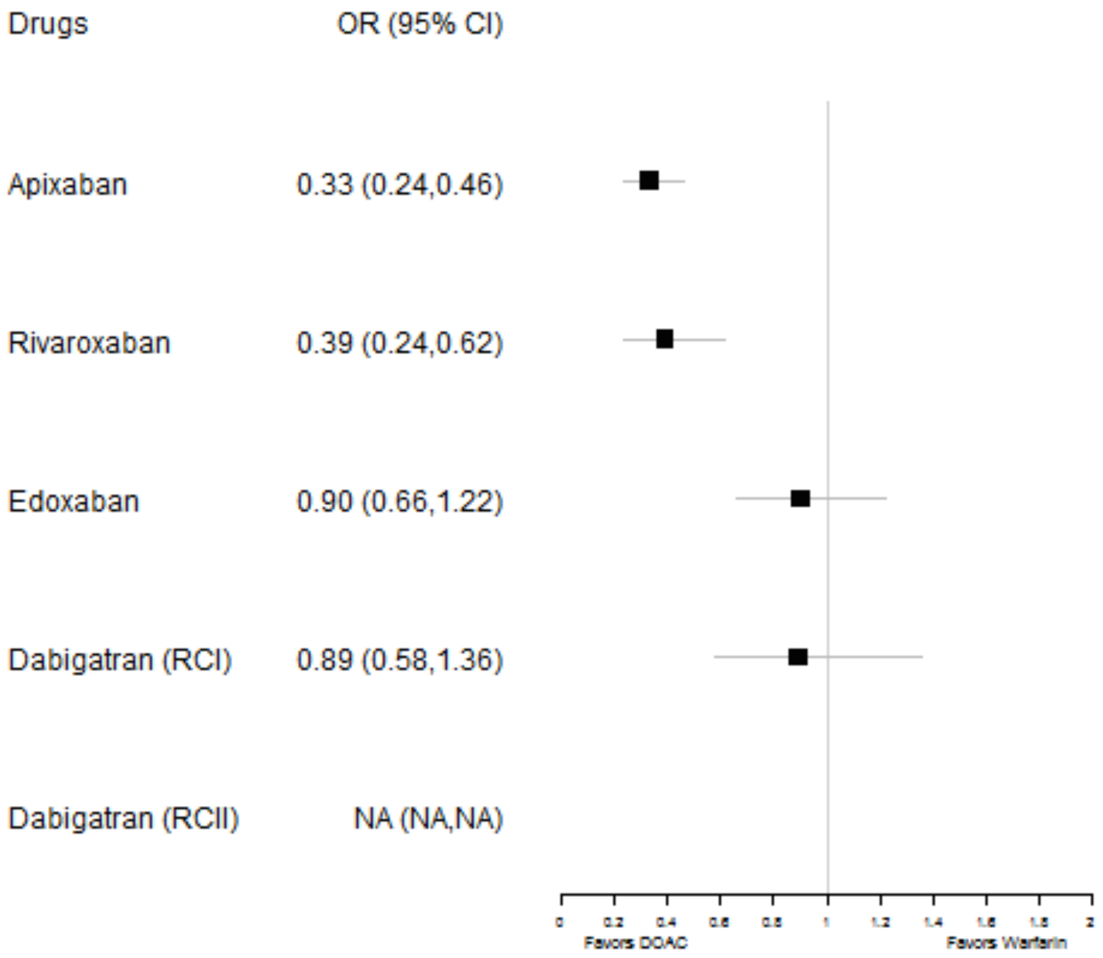

Gastrointestinal bleeding

```
Generalized linear mixed model fit by maximum likelihood (Adaptive Gauss-Hermite
Quadrature, nAGQ = 7) ['glmerMod']
 Family: binomial  ( logit )
Formula: cbind(ADEs[, i], nADEs[, i]) ~ as.factor(arm) + as.factor(id) +      (-1 + arm | id)

    AIC      BIC   logLik deviance df.resid
   19.3     18.2     -4.6      9.3        1

Scaled residuals:
    Min       1Q   Median       3Q      Max
-1.01435 -0.29156 -0.01378  0.25732  0.88046

Random effects:
 Groups Name Variance Std.Dev.
 id     arm  0.1361   0.3689
Number of obs: 6, groups:  id, 3

Fixed effects:
              Estimate Std. Error z value Pr(>|z|)
(Intercept)     -3.50915    0.16817 -20.867  < 2e-16 ***
as.factor(arm)1  0.08666    0.30441   0.285    0.776
as.factor(id)2  -0.08124    0.22098  -0.368    0.713
as.factor(id)4  -1.69853    0.40471  -4.197 2.71e-05 ***
---
Signif. codes:  0 ‘***’ 0.001 ‘**’ 0.01 ‘*’ 0.05 ‘.’ 0.1 ‘ ‘ 1

Correlation of Fixed Effects:
            (Intr) as.()1 as.()2
as.fctr(r)1 -0.058
as.fctr(d)2 -0.647 -0.021
as.fctr(d)4 -0.569 -0.392  0.288
```

Odds Ratio of Studies for Gastrointestinal Bleeding

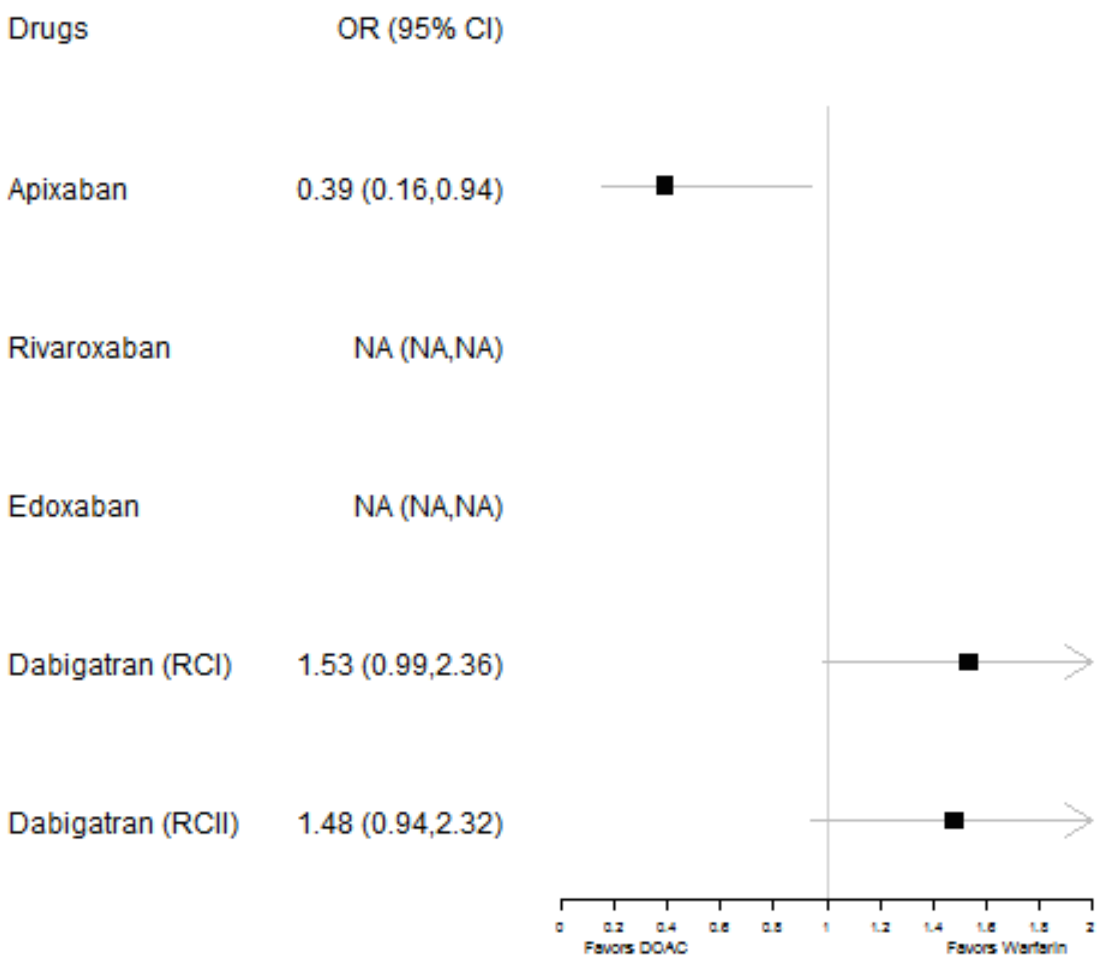

Non-major relevant bleeding

```
Generalized linear mixed model fit by maximum likelihood (Adaptive Gauss-Hermite
Quadrature, nAGQ = 7) ['glmerMod']
 Family: binomial  ( logit )
Formula: cbind(ADEs[, i], nADEs[, i]) ~ as.factor(arm) + as.factor(id) +      (-1 + arm | id)

    AIC      BIC   logLik deviance df.resid
   21.4     20.3     -5.7     11.4        1

Scaled residuals:
    Min       1Q   Median       3Q      Max
-0.41013 -0.24558  0.00297  0.23940  0.31422

Random effects:
 Groups Name Variance Std.Dev.
 id     arm  0.09464  0.3076
Number of obs: 6, groups:  id, 3

Fixed effects:
                Estimate Std. Error z value Pr(>|z|)
(Intercept)     -2.56402    0.09429 -27.193   <2e-16 ***
as.factor(arm)1 -0.31886    0.18927  -1.685    0.092 .
as.factor(id)4   0.09965    0.12153   0.820    0.412
as.factor(id)5   0.24415    0.10743   2.273    0.023 *
---
Signif. codes:  0 '***' 0.001 '**' 0.01 '*' 0.05 '.' 0.1 ' ' 1

Correlation of Fixed Effects:
            (Intr) as.()1 as.()4
as.fctr(r)1 -0.157
as.fctr(d)4 -0.801  0.049
as.fctr(d)5 -0.864  0.086  0.693
```

Odds Ratio of Studies for Non-major Relevent Bleeding

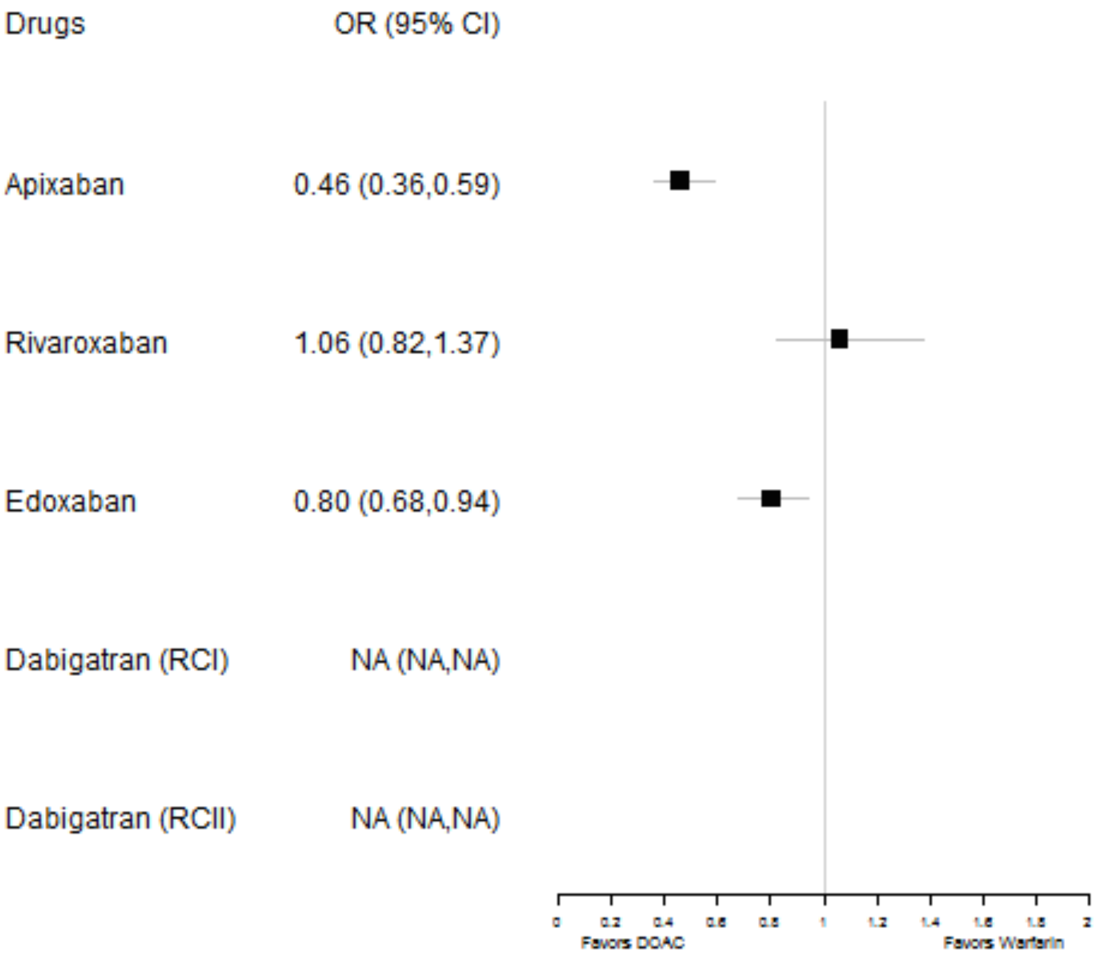

Serious adverse event

```
Generalized linear mixed model fit by maximum likelihood (Adaptive Gauss-Hermite
Quadrature, nAGQ = 7) ['glmerMod']
 Family: binomial  ( logit )
Formula: cbind(ADEs[, i], nADEs[, i]) ~ as.factor(arm) + as.factor(id) +      (-1 + arm | id)

    AIC      BIC   logLik deviance df.resid
   18.7     20.8     -2.4      4.7        3

Scaled residuals:
    Min       1Q   Median       3Q      Max
-0.94906 -0.57324  0.00324  0.57783  0.93815

Random effects:
 Groups Name Variance Std.Dev.
 id     arm  0        0
Number of obs: 10, groups:  id, 5

Fixed effects:
                Estimate Std. Error z value Pr(>|z|)
(Intercept)     -1.93634    0.06332 -30.580  < 2e-16 ***
as.factor(arm)1 -0.03644    0.03969  -0.918 0.358519
as.factor(id)2  -0.03495    0.08546  -0.409 0.682529
as.factor(id)3   0.02282    0.07914   0.288 0.773041
as.factor(id)4   0.25197    0.07109   3.544 0.000394 ***
as.factor(id)5   0.02725    0.06869   0.397 0.691562
---
Signif. codes:  0 ‘***’ 0.001 ‘**’ 0.01 ‘*’ 0.05 ‘.’ 0.1 ‘ ’ 1

Correlation of Fixed Effects:
            (Intr) as.()1 as.()2 as.()3 as.()4
as.fctr(r)1 -0.310
as.fctr(d)2 -0.670  0.001
as.fctr(d)3 -0.723  0.000  0.536
as.fctr(d)4 -0.805  0.001  0.597  0.644
as.fctr(d)5 -0.834  0.001  0.617  0.667  0.742
```

Odds Ratio of Studies for Serious Adverse Event

Drugs	OR (95% CI)
Apixaban	1.03 (0.89,1.19)
Rivaroxaban	0.84 (0.69,1.03)
Edoxaban	0.92 (0.81,1.05)
Dabigatran (RCI)	1.11 (0.88,1.41)
Dabigatran (RCII)	1.03 (0.81,1.31)

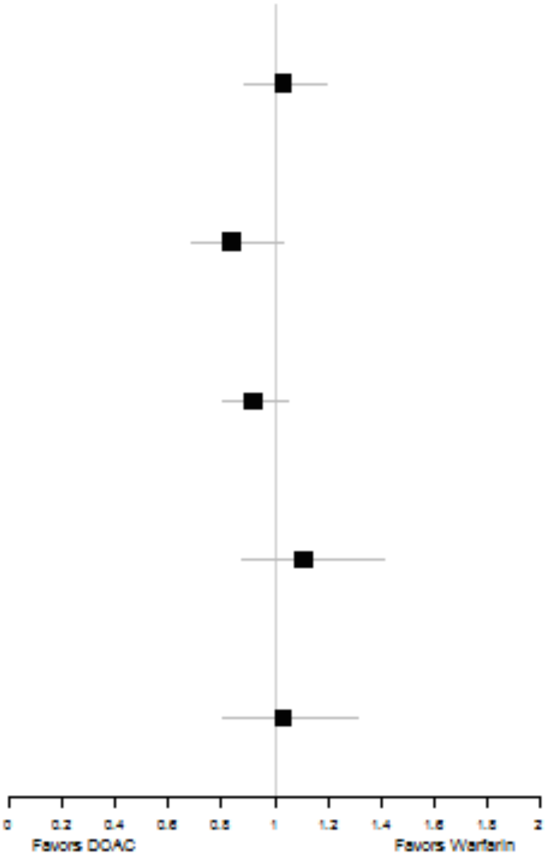

Medication stopped (ADE)

```
Generalized linear mixed model fit by maximum likelihood (Adaptive Gauss-Hermite
Quadrature, nAGQ = 7) ['glmerMod']
 Family: binomial  ( logit )
Formula: cbind(ADEs[, i], nADEs[, i]) ~ as.factor(arm) + as.factor(id) +      (-1 + arm | id)

    AIC      BIC   logLik deviance df.resid
   20.8     22.9     -3.4      6.8        3

Scaled residuals:
    Min       1Q   Median       3Q      Max
-1.27533 -0.57082  0.00149  0.56514  1.28553

Random effects:
 Groups Name Variance Std.Dev.
 id     arm  0        0
Number of obs: 10, groups:  id, 5

Fixed effects:
              Estimate Std. Error z value Pr(>|z|)
(Intercept)     -2.30305    0.07648 -30.112  < 2e-16 ***
as.factor(arm)1 -0.02615    0.06473  -0.404    0.686
as.factor(id)2  -0.13932    0.10084  -1.382    0.167
as.factor(id)3  -0.66234    0.10559  -6.273 3.55e-10 ***
as.factor(id)4  -0.40262    0.08961  -4.493 7.02e-06 ***
as.factor(id)5  -2.16771    0.12574 -17.239  < 2e-16 ***
---
Signif. codes:  0 '***' 0.001 '**' 0.01 '*' 0.05 '.' 0.1 ' ' 1

Correlation of Fixed Effects:
            (Intr) as.()1 as.()2 as.()3 as.()4
as.fctr(r)1 -0.420
as.fctr(d)2 -0.626  0.002
as.fctr(d)3 -0.597  0.001  0.453
as.fctr(d)4 -0.704  0.002  0.533  0.509
as.fctr(d)5 -0.502  0.001  0.380  0.363  0.428
```

Odds Ratio of Studies for Medication Stopped (ADE)

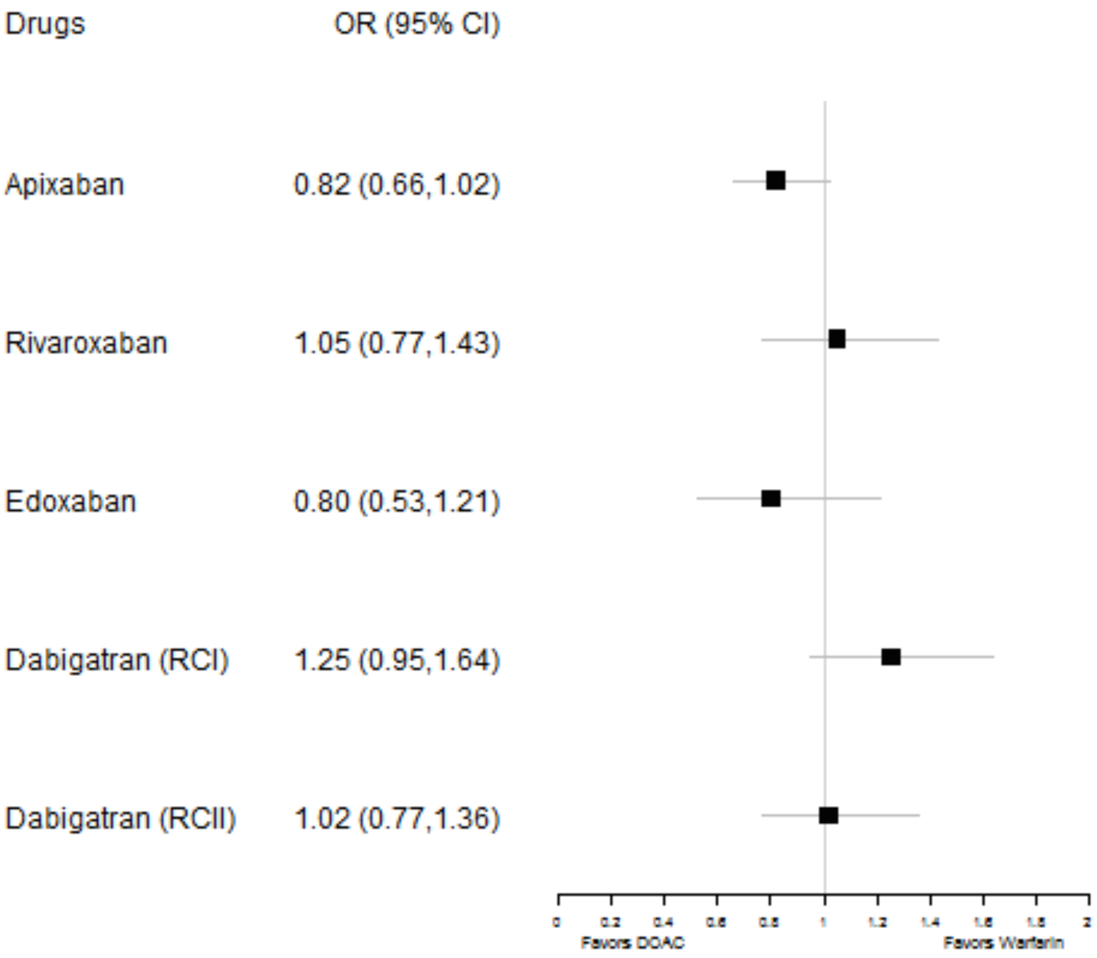

Supplement: S9 File — (PDF) [file pone.0197583.s009.pdf]
