# Supplementary material for: Efficacy and safety of direct oral anticoagulants approved for cardiovascular indications: Systematic review and meta-analysis
Source: PLoS One. 2018 May 24;13(5):e0197583. doi: 10.1371/journal.pone.0197583 (PMC5967718; doi:10.1371/journal.pone.0197583)
Supplement: S10 File — (DOCX) [file pone.0197583.s010.docx]

**Previous Meta-analyses**

Supplement to: Makam RCP, Hoaglin DC, McManus DD, Wang V, Gore JM, Spencer FA, Pradhan R, Tran H, Yu H, Goldberg RJ. Efficacy and safety of direct oral anticoagulants approved for cardiovascular indications: systematic review and meta-analysis.

From published references and other sources, we located 31 previous meta-analyses that included at least one DOAC: 2 for both NVAF and VTE, 16 for NVAF only, 7 for VTE only, and 6 that pooled data for a mixture of indications. (The tables below list the studies in these four categories in reverse chronological order.) Unfortunately, none of those meta-analyses produced results that were relevant, reliable, and adequately documented.

Their relevance is limited for several reasons. Most of them do not include the ENGAGE AF – TIMI 48 trial of edoxaban versus warfarin (Giugliano et al. 2013). Also, some include Phase 2 trials, DOACs at unapproved dosages, experimental indications, or DOACs that have not been approved by the FDA or have been withdrawn from use in clinical practice.

The other major limitation is the use of unreliable or inappropriate methods of analysis. It seems that the authors do not have sufficient expertise in meta-analysis and have not obtained expert advice on methods (a shortcoming that editorial reviews overlooked). Instead, most of them relied on widely available software, such as the Cochrane Collaboration’s Review Manager (RevMan) and the commercial software Comprehensive Meta-Analysis (CMA). That trust, however, was misplaced. Because of the strong possibility of heterogeneity among trials, most of the meta-analyses appropriately used a random-effects method. Unfortunately, the default random-effects method in RevMan and CMA is the method of DerSimonian and Laird (1986) (DL), which “can produce biased estimates with falsely high precision” (Cornell et al. 2014). Although evidence of the shortcomings of DL has been accumulating for many years in the meta-analysis literature, the documentation for those and other software packages has not included warnings.

One source of difficulty in the DL method is its use of “inverse-variance” weights based on estimated variances. A few authors used inverse-variance weights in analytic methods other than DL.

Some authors (Dogliotti et al. 2013, Douxfils et al. 2014, and Holster et al. 2013) used a test based on a statistic known as Cochran’s *Q* to choose between a fixed-effect analysis and a random-effects analysis. Borenstein et al. (2010) explain why that practice is incorrect. They emphasize in their summary: “The strategy of starting with a fixed-effect model and then moving to a random-effects model if the test for heterogeneity is significant relies on a flawed logic and should be strongly discouraged.”

The test based on *Q* has a fundamental problem: it uses an incorrect null distribution. Hoaglin (2016) reviews results on the behavior of *Q*.

Another example involves confusion about the Mantel-Haenszel method. For example, Providência et al. (2014) say, “Data were pooled using random-effects, according to the Mantel-Haenszel model (Review Manager, RevMan, Version 5.1. Copenhagen: The Nordic Cochrane Center, The Cochrane Collaboration, 2011).” And their forest plots, produced by RevMan, contain the misleading label “M-H, Random”. In fact, the Mantel-Haenszel method is only a fixed-effect method. This confusion appears also in van der Hulle et al. (2014) and in the supplementary figures of van Es et al. (2014).

Dentali et al. (2012) make a puzzling statement. The article refers to exact estimates and cites Sweeting et al. (2004), but that paper does not discuss exact estimates.

Over half of the articles use relative risk (RR) as the measure of effect, but it often not clear whether RR is the ratio of rates (i.e., the ratio of the rate of occurrence of the ADE in the treatment arm to the corresponding rate in the control arm) or actually the hazard ratio (from a proportional-hazards model).

Because no trial has studied more than one DOAC, some authors estimate indirect treatment comparisons, “anchored” on a common comparator. For example, if DOAC A and DOAC B have been compared with adjusted-dose warfarin therapy in separate trials, and the warfarin treatments, populations, and designs are nearly the same, an indirect comparison of DOAC A with DOAC B can use the difference between the direct comparison of A with warfarin and the direct comparison of warfarin with B. If the measure of effect is the odds ratio, the indirect estimate usually takes the form of a difference on the log-odds-ratio scale. A standard error for the indirect estimate follows from a basic result in probability: If *X* and *Y* are independent random variables,

$\mathrm{var}(X-Y)=\mathrm{var}(X)+\mathrm{var}(Y)$.

Some authors refer to these calculations for an indirect treatment comparison as “Bucher’s method” (1997). From the formula it is clear that the standard error for the indirect comparison is larger than the standard error for either of the two direct comparisons on which it is based. When each of these direct comparisons comes from only one trial (or perhaps two trials), the indirect comparison is unlikely to be very precise. The situation is more favorable when each direct comparison is the result of a pairwise meta-analysis of several sizable trials.

Schneeweiss et al. (2012) discuss requirements for indirect comparisons. They emphasize “the assumption that the trials or subgroups within trials are sufficiently similar with respect to potential clinical and methodological modifiers of relative treatment effects, such as patient characteristics, intervention characteristics, follow-up time, outcome definitions and ascertainment (clinical modifiers), and randomization and blinding (methodological modifiers).” They point out that Lip et al. (2012) failed to assure comparability of trials in two ways. First, ROCKET-AF required a CHADS_2_ score ≥ 2, whereas the other trials (ARISTOTLE and RE-LY) also included patients with scores of 0 and 1. Second, they contrasted the rates of major bleeding from an intention-to-treat analysis of RE-LY and the results from the on-treatment analyses of ARISTOTLE and ROCKET-AF.

When the indirect comparison is part of a substantial network of evidence that contains enough direct comparisons, methods of network meta-analysis can often estimate indirect comparisons more precisely. (Cooper et al. 2006 analyze a network containing 10 treatments, 19 trials, and a total of 45 arms.) Cohen et al. (2015) report results of a sophisticated Bayesian network meta-analysis; but their otherwise commendable supporting information contains no details of the analysis, some of which are necessary for understanding the results. Castellucci et al. (2014) also carried out a Bayesian network meta-analysis, but they did not give the details of how they obtained estimates of hazard ratios from their Poisson likelihood model (hazard ratios are usually associated with survival analysis, whereas a Poisson distribution is appropriate for events that can occur more than once and would require data on the numbers of events). The network meta-analysis in Roskell et al. (2010) used a mixed log-binomial model with a fixed overall treatment effect, a random trial effect (“to account for outcomes within trial being correlated”—puzzling, because randomization implies lack of correlation between arms), and a fixed effect for mean length of follow-up. They do not mention the constraint required to ensure that the binomial probabilities do not exceed 1.

Sterne et al. (2017) used sophisticated Bayesian methods to analyze networks of evidence for prevention of stroke in AF (a separate network for each of 7 outcomes, with numbers of interventions ranging from 9 to 25, in a total of 23 trials) and for acute treatment of VTE (a separate network for each of 7 outcomes, with numbers of interventions ranging from 5 to 13, in a total of 9 trials). Because each distinct dosage (AF: 2 dosages for apixaban, 4 for dabigatran, 6 for dabigatran + aspirin, 5 for edoxaban, 1 for rivaroxaban, 3 for betrixaban, 2 for antiplatelet, and 2 for warfarin; VTE: 3 for apixaban, 1 for dabigatran, 1 for edoxaban, 7 for rivaroxaban, and 1 for warfarin) was a separate node, the network meta-analyses yielded direct comparisons (with VKAs) for the currently approved dosages of the DOACs. Most of those direct comparisons for AF, however, included data from Phase 2 trials, and the comparison of rivaroxaban with warfarin for VTE included the data from the EINSTEIN PE trial. Because the indirect comparisons between DOACs are anchored on warfarin, they also involve those data. Sterne et al. give a good general description of their Bayesian analyses, but they do not report some key details (e.g., the prior distributions for the parameters in their models).

**References**

Adam SS, McDuffie JR, Ortel TL, Williams JW Jr (2012). Comparative effectiveness of warfarin and new oral anticoagulants for the management of atrial fibrillation and venous thromboembolism: a systematic review. *Annals of Internal Medicine* 157:796-807.

Artang R, Rome E, Nielsen JD, Vidaillet HJ (2013). Meta-analysis of randomized controlled trials on risk of myocardial infarction from the use of oral direct thrombin inhibitors. *American Journal of Cardiology* 112:1973-1979.

Baker WL, Phung OJ (2012). Systematic review and adjusted indirect comparison meta-analysis of oral anticoagulants in atrial fibrillation. *Circulation: Cardiovascular Quality and Outcomes* 5:711-719.

Borenstein M, Hedges LV, Higgins JPT, Rothstein H (2010). A basic introduction to fixed-effect and random-effects models for meta-analysis. *Research Synthesis Methods* 1:97-111.

Bucher HC, Guyatt GH, Griffith LE, Walter SD (1997). The results of direct and indirect treatment comparisons in meta-analysis of randomized controlled trials. *Journal of Clinical Epidemiology* 50:683-691.

Capodanno D, Capranzano P, Giacchi G, Calvi V, Tamburino C (2013). Novel oral anticoagulants versus warfarin in non-valvular atrial fibrillation: a meta-analysis of 50,578 patients. *International Journal of Cardiology* 167:1237-1241.

Castellucci LA, Cameron C, Le Gal G, Rodger MA, Coyle D, Wells PS, Clifford T, Gandara E, Wells G, Carrier M (2014). Clinical and safety outcomes associated with treatment of acute venous thromboembolism: a systematic review and meta-analysis. *JAMA* 312:1122-1135.

Cohen AT, Hamilton M, Mitchell SA, Phatak H, Liu X, Bird A, Tushabe D, Batson S (2015). Comparison of the novel oral anticoagulants apixaban, dabigatran, edoxaban, and rivaroxaban in the initial and long-term treatment and prevention of venous thromboembolism: systematic review and network meta-analysis. *PLoS ONE* 10(12):e0144856.

Cooper NJ, Sutton AJ, Lu G, Khunti K (2006). Mixed comparison of stroke prevention treatments in individuals with nonrheumatic atrial fibrillation. *Archives of Internal Medicine* 166:1269-1275.

Cooper NJ, Sutton AJ, Morris D, Ades AE, Welton NJ (2009). Addressing between-study heterogeneity and inconsistency in mixed treatment comparisons: applications to stroke prevention treatments in individuals with non-rheumatic atrial fibrillation. *Statistics in Medicine* 28:1861-1881.

Cornell JE, Mulrow CD, Localio R, Stack CB, Meibohm AR, Guallar E, Goodman SN (2014). Random-effects meta-analysis of inconsistent effects: a time for change. *Annals of Internal Medicine* 160:267-270.

Dentali F, Riva N, Crowther M, Turpie AGG, Lip GYH, Ageno W (2012). Efficacy and safety of the novel oral anticoagulants in atrial fibrillation: a systematic review and meta-analysis of the literature. *Circulation* 126:2381-2391.

DerSimonian R, Laird N (1986). Meta-analysis in clinical trials. *Controlled Clinical Trials* 7:177-188.

Dogliotti A, Paolasso E, Giugliano RP (2013). Novel oral anticoagulants in atrial fibrillation: a meta-analysis of large, randomized, controlled trials vs warfarin. *Clin*

*Cardiol* 36:61-67.

Douxfils J, Buckinx F, Mullier F, Minet V, Rabenda V, Reginster J-Y, Hainaut P, Bruyère O, Dogné J-M (2014). Dabigatran etexilate and risk of myocardial infarction, other cardiovascular events, major bleeding, and all-cause mortality: a systematic review and meta-analysis of randomized controlled trials. *Journal of the American Heart Association* 3:e000515.

Fleming TR, Emerson SS (2011). Evaluating rivaroxaban for nonvalvular atrial fibrillation— regulatory considerations. *New England Journal of Medicine* 365:1557-1559.

Fox BD, Kahn SR, Langleben D, Eisenberg MJ, Shimony A (2012). Efficacy and safety of novel oral anticoagulants for treatment of acute venous thromboembolism: direct and adjusted indirect meta-analysis of randomised controlled trials. *BMJ* 345:e7498.

Giugliano RP, Ruff CT, Braunwald E, et al. (2013). Edoxaban versus warfarin in patients with atrial fibrillation. *New England Journal of Medicine* 369:2093-2104.

Hart RG, Pearce LA, Aguilar MI (2007). Meta-analysis: antithrombotic therapy to prevent stroke in patients who have nonvalvular atrial fibrillation. *Annals of Internal Medicine* 146:857-867.

Hoaglin DC (2016). Misunderstandings about Q and “Cochran’s Q test” in meta-analysis. *Statistics in Medicine* 35:485-504. With discussion and a reply by the author.

Holster IL, Valkhoff VE, Kuipers EJ, Tjwa ETTL (2013). New oral anticoagulants increase risk for gastrointestinal bleeding: a systematic review and meta-analysis. *Gastroenterology* 145:105-112.

Jia B, Lynn HS, Rong F, Zhang W (2014). Meta-analysis of efficacy and safety of the new anticoagulants versus warfarin in patients with atrial fibrillation. *Journal of Cardiovascular Pharmacology* 64:368-374.

Kang N, Sobieraj DM (2014). Indirect treatment comparison of new oral anticoagulants for the treatment of acute venous thromboembolism. *Thrombosis Research* 133:1145-1151.

Lip GYH, Edwards SJ (2006). Stroke prevention with aspirin, warfarin and ximelagatran in patients with non-valvular atrial fibrillation: a systematic review and meta-analysis. *Thrombosis Research* 118:321-333.

Lip GYH, Larsen TB, Skjøth F, Rasmussen LH (2012). Indirect comparisons of new oral anticoagulant drugs for efficacy and safety when used for stroke prevention in atrial fibrillation. *Journal of the American College of Cardiology* 60:738-746.

Mak K-H (2012). Coronary and mortality risk of novel oral antithrombotic agents: a meta-analysis of large randomised trials. *BMJ Open* 2:e001592.

Mantha S, Ansell J (2015). Indirect comparison of dabigatran, rivaroxaban, apixaban, and edoxaban for the treatment of acute venous thromboembolism. *Journal of Thrombosis and Thrombolysis* 39:155-165.

Miller CS, Grandi SM, Shimony A, Filion KB, Eisenberg MJ (2012). Meta-analysis of efficacy and safety of new oral anticoagulants (*dabigatran*, *rivaroxaban*, *apixaban*) versus warfarin in patients with atrial fibrillation. *American Journal of Cardiology* 110:453-460.

Providência R, Grove EL, Husted S, Barra S, Boveda S, Morais J (2014). A meta-analysis of phase III randomized controlled trials with novel oral anticoagulants in atrial fibrillation: comparisons between direct thrombin inhibitors vs. factor Xa inhibitors and different dosing regimens. *Thrombosis Research* 134:1253-1264.

Rasmussen LH, Larsen TB, Graungaard T, Skjøth F, Lip GYH (2012). Primary and secondary prevention with new oral anticoagulant drugs for stroke prevention in atrial fibrillation: indirect comparison analysis. *BMJ* 345:e7097.

Roskell NS, Lip GYH, Noack H, Clemens A, Plumb JM (2010). Treatments for stroke prevention in atrial fibrillation: a network meta-analysis and indirect comparisons versus dabigatran etexilate. *Thrombosis and Haemostasis* 104:1106-1115.

Ruff CT, Giugliano RP, Braunwald E, Hoffman EB, Deenadayalu N, Exekowitz MD, Camm AJ, Weitz JI, Lewis BS, Parkhomenko A, Yamashita T, Antman EM (2014). Comparison of the efficacy and safety of new oral anticoagulants with warfarin in patients with atrial fibrillation: a meta-analysis of randomized trials. *Lancet* 383:955-962.

Sardar P, Chatterjee S, Lavie CJ, Giri JS, Ghosh J, Mukherjee D, Lip GYH (2015). Risk of major bleeding in different indications for new oral anticoagulants: insights from a meta-analysis of approved dosages from 50 randomized trials. *International Journal of Cardiology* 179:279-287.

Schneeweiss S, Gagne JJ, Patrick AB, Choudhry NK, Avorn J (2012). Comparative efficacy and safety of new oral anticoagulants in patients with atrial fibrillation. *Circulation: Cardiovascular Quality and Outcomes* 5:480-486.

Sterne JAC, Bodalia PN, Bryden PA, Davies PA, et al. (2017). Oral anticoagulants for primary prevention, treatment and secondary prevention of venous thromboembolic disease, and for prevention of stroke in atrial fibrillation: systematic review, network meta-analysis and cost-effectiveness analysis. *Health Technology Assessment* 21:9.

Sweeting MJ, Sutton AJ, Lambert PC (2004). What to add to nothing? Use and avoidance of continuity corrections in meta-analysis of sparse data. *Statistics in Medicine* 23:1351-1375.

Uchino K, Hernandez AV (2012). Dabigatran association with higher risk of acute coronary events. *Archives of Internal Medicine* 172:397-402.

van der Hulle T, Kooiman J, den Exter PL, Dekkers OM, Klok FA, Huisman MV. (2014). Effectiveness and safety of novel oral anticoagulants as compared with vitamin K antagonists in the treatment of acute symptomatic venous thromboembolism: a systematic review and meta-analysis. *Journal of Thrombosis and Haemostasis* 12:320-328.

van Es N, Coppens M, Schulman S, Middeldorp S, Büller HR (2014). Direct oral anticoagulants compared with vitamin K antagonists for acute venous thromboembolism: evidence from phase 3 trials. *Blood* 124:1968-1975.

| Article containing meta-analyses for NVAF and, separately, VTE | | | | | | | | | | |
| --- | --- | --- | --- | --- | --- | --- | --- | --- | --- | --- |
| Article | DOACs:  NVAF | DOACS:  VTE | Other  treatments | Outcomes:  NVAF | Outcomes:  VTE | RCTs:  NVAF | RCTs:  VTE | Effect  measure | Meta-analysis  method | Comments |
| Sterne  2017 | api  dab  edo  riv | api  dab  edo  riv | NVAF:  betrixaban  antiplatelet | Stroke or SE  Isch stroke  MI  Maj bleeding  CR bleeding  IC bleeding  All-cause  mort | Symp VTE  Symp DVT  Symp PE  MI  Major  bleeding  CR bleeding  All-cause  mort | 23 | 9 | OR | Bayesian  NMA, FE  Details not  given | Included  Phase 2  trials  VTE  included  EINSTEIN-PE |
| Adam  2012 | api  dab  riv | dab  riv |  | Death  Hem stroke  Isch stroke | Death  Recur DVT/PE  TE death | 3 | 3 | RR | DL | VTE  included  EINSTEIN-PE |
| Abbreviations: CR, clinically relevant; DVT, deep-vein thrombosis; FE, fixed-effect; IC, intracranial; MI, myocardial infarction; NMA, network meta-analysis; PE, pulmonary embolism; SE, systemic embolism; TE, thromboembolism | | | | | | | | | | |

| Articles containing meta-analyses for NVAF (only): DOACs vs. dose-adjusted warfarin | | | | | | | |
| --- | --- | --- | --- | --- | --- | --- | --- |
| Article | DOACs | Other  treatments | Outcomes | RCTs | Effect  measure | Meta-analysis  method | Comments |
| Jia  2014 | api  dab  edo  riv |  | Stroke and  syst emb  Isch stroke  Hem stroke  MI  All-cause mort  Maj bleeding  IC hem  GI bleeding | 5 | RR | DL | Included  J-ROCKET AF |
| Providência  2014 | api  dab  edo  riv  xim |  | Stroke and  syst emb  Total mort  Cardiovasc mort  Isch stroke  Acute MI  Maj bleeding  IC bleeding  GI bleeding | 7 | RR | RE, M-H | Included  J-ROCKET AF |
| Ruff  2014 | api  dab  edo  riv |  | Stroke or  syst emb  Isch stroke  Hem stroke  MI  All-cause mort  Maj bleeding  IC hem  GI bleeding | 4 | RR | DL | Had access  to clinical  database in  ENGAGE AF  – TIMI 48 |
| Capodanno  2013 | api  dab  riv |  | Stroke or  syst emb  Isch stroke  Hem stroke  All-cause mort  MI  Maj bleeding  IC hem  GI bleeding  ALT or AST > 3×  ULN with conc  bilirubin > 2×ULN | 3 | OR | FE (IVW?),  confirmed by  RE (DL?) |  |
| Dogliotti  2013 | api  dab  riv  xim |  | Stroke or  syst emb  All-cause mort  Isch stroke  Syst embolism  Hem stroke  Maj bleeding | 5 | RR | FE, M-H if  *P* > .10 in test  based on *Q*,  else RE, DL |  |
| Baker  2012 | api  dab  riv |  | Stroke or  syst emb  Any stroke  Isch stroke  Syst embolism  Mortality  Maj bleeding  Hem stroke  GI bleeding | 4 | RR | Pairwise MA  by RE, DL  ITC, IVW  RE meta-  regression on  mean CHADS_2_  score and TTR | Included  PETRO  (Phase 2) |
| Dentali  2012 | api  dab  edo  riv |  | Total mort  Cardiovasc mort  Stroke or SE  Isch stroke  Maj bleeding  IC bleeding  MI | 12  (4 Ph 3,  8 Ph 2) | RR | FE, M-H  compared  with RE, DL  Cites Sweeting  (2004) for  exact estimates | No Phase 3  trial of edo.  Included  J-ROCKET AF |
| Lip  2012 | api  dab  riv |  | Stroke or  syst emb  Any stroke  Isch or uncertain  type of stroke  Hem stroke  Death (any cause)  MI  Maj bleeding  IC bleeding | 3 | HR | ITC | Interpreted  HR in RE-LY  as RR |
| Miller  2012 | api  dab  riv |  | Stroke or  syst emb  Isch or unspec  type of stroke  Hem stroke  All-cause mort  Vascular mort  Maj bleeding  GI bleeding  IC bleeding | 3 | RR | RE, DL |  |
| Rasmussen  2012 | api  dab  riv |  | Stroke or  syst emb  Any stroke  Isch or uncertain  type of stroke  Hem stroke  Disabling or  fatal stroke  Death (any cause)  MI  Maj bleeding  IC bleeding  GI bleeding  Other location  bleeding | 3 | HR | ITC  (no formal  systematic  review and MA) | Primary and  Secondary  prevention |
| Schneeweiss  2012 | api  dab  riv |  | Stroke or  syst emb  All-cause mort  Maj bleeding  2-yr discont rate | 3 | HR | Indirect  comparisons | Noted that  ROCKET-AF  participants  required  CHADS2 ≥ 2.  Also compared  subgroup with  CHADS2 ≥ 3. |
| Roskell  2010 | dab  xim | Aspirin  monother,  Aspirin plus  clopidogrel,  FLD warfarin,  FLD warfarin  plus aspirin,  indobufen,  triflusal,  idraparinux,  placebo | Any stroke  Isch stroke  Syst embolism  All-cause mort  IC hem (excl hem  stroke)  Extracran hem  (major bleeds)  Acute MI | 20 | RR | NMA, mixed log-  binomial model:  fixed trt effect,  random study  effect, fixed  effect for mean  length of  follow-up;  ITC vs. dab | Unusual  model |
| Cooper  2009 | xim | 16 other trts  in evidence  network | Stroke | 25 | Log(OR) | Bayesian NMA | Methods  paper:  extends NMA  to include  study-level  covariates |
| Hart  2007 | dab  xim | 15 other trts | Any stroke  Isch stroke  IC hem  All-cause mort  Major extracran  hem | 29 | Rel risk  reduction  derived  from  combined  OR,  Absolute  risk red | RE, DL:  Pairwise  comparisons of  adj-dose warfarin  vs. placebo or  control, adj-dose  warfarin vs.  antiplatelet  agents, and  antiplatelet  agents vs. placebo  or control | Data on dab  from PETRO  (Phase 2).  Prelim test  based on Q;  lack of homog  precluded  estimation of  overall trt  effect. |
| Lip  2006 | xim | FLD warfarin,  FLD warfarin  plus aspirin,  aspirin,  placebo | Isch stroke  Syst embolism  Mortality  Hemorrhage | 14 | RR | FE, M-H  Sens anal: RE, DL |  |
| Abbreviations: ALT, alanine aminotransferase; AST, aspartate aminotransferase; DL, DerSimonian-Laird;  FE, fixed-effect; FLD, fixed low-dose; GI, gastrointestinal; IC, intracranial; ITC, indirect treatment comparison;  IVW, inverse-variance weights; M-H, Mantel-Haenszel; MI, myocardial infarction; NMA, network meta-analysis; RE, random-effects; TTR, time within therapeutic range; ULN, upper limit of normal | | | | | | | |

| Articles containing meta-analyses for VTE: DOACs vs. conventional therapy | | | | | | | |
| --- | --- | --- | --- | --- | --- | --- | --- |
| Article | DOACs | Other  treatments | Outcomes | RCTs | Effect  measure | Meta-analysis  method | Comments |
| Cohen  2015 | api  edo  dab  riv |  | VTE and VTE-  related death  Maj or CRNM  bleeding  Maj bleeding  CRNM bleeding  All-cause mort | 6 | Relative  risk | Bayesian NMA,  FE  Details not  given | Initial and  long-term  trt and  prevention  Used pooled  data from  EINSTEIN DVT  and EINSTEIN PE |
| Mantha  2015 | api  dab  edo  riv |  | Recurrent VTE  Maj bleeding  Mortality  Maj or CRNM  bleeding | 6 | HR  If not avail,  used RR or  OR | ITC  IVW for the 2  trials of dab  and the 2  trials of riv |  |
| Castellucci  2014 | api  dab+LMWH  edo+LMWH  riv | UFH+VKA  fondaparinux  +VKA  LMWH alone | Recurrent VTE  Maj bleeding  Fatal recurrent  VTE  Fatal bleeding | 45 | HR | Bayesian RE  NMA using  Poisson  likelihood  model  Frequentist  pairwise MAs  Details not  reported |  |
| Kang  2014 | api  dab  edo  riv |  | Mortality  Recurrent VTE  Recurrent PE  Recurrent DVT  Maj bleeding | 6 | RR | ITC  Traditional  RE MA for  the 2 trials  of dab and the  2 trials of riv |  |
| van der Hulle  2014 | api  dab  edo  riv |  | Recurrent VTE  Fatal PE  Overall mort  Maj bleeding  Non-fatal maj  bleeding at a  critical site  CRNM bleeding  Non-fatal IC  bleeding  Maj GI bleeding  Fatal bleeding  during anticoag  trt | 5 | RR | RE, M-H  FE NMA IVW  with riv as  comparator | RECOVER II not  yet published  Included  EINSTEIN PE |
| van Es  2014 | api  dab  edo  riv |  | Recurrent VTE  or VTE-related  death  Maj bleeding  IC bleeding  Fatal bleeding  Maj GI bleeding  CRNM bleeding  All-cause mort  Net clinical  benefit | 6 | Relative  risk  (calculated) | RE, M-H and DL | Included  EINSTEIN PE |
| Fox  2012 | api  dab  riv  xim |  | Recurrent VTE  Maj bleeding  All-cause mort | 9 | Risk ratio | RE, DL  ITC | Had only a Ph 2  trial for api  For dab, had  RECOVER II only  in abstract  Included  EINSTEIN PE and  2 Ph 2 trials for  riv |
| Abbreviations: CRNM, clinically relevant non-major; DL, DerSimonian-Laird; DVT, deep-vein thrombosis;  FE, fixed-effect; GI, gastrointestinal; HR, hazard ratio; IC, intracranial; ITC, indirect treatment comparison;  IVW, inverse-variance weights; LMWH, low-molecular-weight heparin; M-H, Mantel-Haenszel;  NMA, network meta-analysis; PE, pulmonary embolism;  RE, random-effects; UFH, unfractionated heparin; VKA, vitamin K antagonist | | | | | | | |

| Articles containing meta-analyses for a mixture of indications | | | | | | | | |
| --- | --- | --- | --- | --- | --- | --- | --- | --- |
| Article | DOACs | Other  treatments | Indications | Outcomes | RCTs | Effect  measure | Meta-analysis  method | Comments |
| Sardar  2015 | api  dab  dar  edo  riv | Aspirin  Dalteparin  Enoxaparin  Placebo | NVAF  Acute VTE  Extended trt  of VTE  Thrombopro-  phylaxis after  hip surgery  Thrombopro-  phylaxis after  knee surgery  Acute coronary  syndrome  Thrombopro-  phylaxis in  medically ill  patients | Maj bleeding | 50 | OR | RE, DL  Sens anal:  FE, M-H  ITC | NVAF: The 10  RCTs included  J-ROCKET AF,  a Ph 3 trial of  api vs. aspirin,  and 5 Ph 2  trials  Acute VTE: The  8 RCTs included  EINSTEIN PE  and 3 Ph 2  trials, but not  RECOVER II |
| Douxfils  2014 | dab | Comparator  regimens:  Adj-dose  warfarin,  Enoxaparin,  Placebo,  Warfarin | NVAF  Acute VTE  Prevent VTE  recurrence  Prophylaxis in  hip repl  Prophylaxis in  knee repl  Acute coronary  syndrome  Patients under-  going or having  undergone  implantation of  mechanical valve | MI  Other cardio-  vasc events  Maj bleeding  All-cause mort | 14 | OR | FE, Peto  RE, DL if *Q* had  *P* < .10 or if  *I*^2^ > 50% | MAs stratified  by control  regimen, but  not by indication |
| Artang  2013 | api  dab  riv  xim  AZD0837 | Aspirin  Clopidogrel  Idraparinux | NVAF  Acute VTE  VTE  Mech valves  Thrombopro-  phylaxis after  knee repl | MI | 15 | OR | FE, M-H  RE, DL if *I*^2^ >  50% | Combined dab  150 and 110 mg  results from  RE-LY |
| Holster  2013 | api  dab  edo  riv | Standard  of care | NVAF  Acute VTE  Prevent VTE  after orthopedic  surgery  Prevent VTE in  medically ill  Treat ACS | GI bleeding  Clin relevant  bleeding | 43 | OR | RE, DL if *Q* had  *P* < .10 and  *I*^2^ > 50% | NVAF: The 8  RCTs included  ARISTOTLE-J,  a Ph 3 trial of  api vs. aspirin,  and 3 Ph 2  trials  VTE: The 7 RCTs included  EINSTEIN PE,  EINSTEIN EXTENSION  and 3 Ph 2  trials, but not  AMPLIFY or  Hokusai-VTE |
| Mak  2012 | api  dab  riv  xim | Comparator  regimens:  Aspirin  Enoxaparin  Placebo | NVAF  Acute VTE  Extended VTE  therapy  Prophylaxis in  hip surgery  Prophylaxis in  knee surgery  Trt of acute  coronary  syndrome | MI or ACS  Maj bleeding  All-cause mortality | 28 | OR | RE, DL | Each MA compares a single DOAC vs. “control” (a mixture of warfarin,  enoxaparin, aspirin, and  placebo) by combining  various indications.  Comparisons among  DOACs are informal.  Abst says “Design: Mixed  treatment comparison meta-analysis,” but  article reports none. |
| Uchino  2012 | dab | Comparator  regimens:  Adj-dose  Warfarin  Enoxaparin  Placebo | NVAF  Acute DVT  Prophylaxis in  hip repl  Prophylaxis in  knee repl  Acute  coronary  syndrome | MI  ACS if study did not report  MI  Overall mort | 7 | OR  Sens  anal:  RR  Risk diff | FE, M-H  Sens  anal:  Peto  IVW  RE  RE meta-  regr on  risk in  control  groups | Two Ph 2 trials  The 3 studies of prophy-  laxis in joint repl were  short-term |
| Abbreviations: ACS, acute coronary syndrome; dar, darexaban; DL, DerSimonian-Laird; DVT, deep-vein thrombosis;  FE, fixed-effect; GI, gastrointestinal; ITC, indirect treatment comparison; IVW, inverse-variance weights;  M-H, Mantel-Haenszel; MI, myocardial infarction; RE, random-effects | | | | | | | | |
